# Characterisation of mouse epididymosomes reveals a complex profile of microRNAs and a potential mechanism for modification of the sperm epigenome

**DOI:** 10.1038/srep31794

**Published:** 2016-08-23

**Authors:** Jackson N. Reilly, Eileen A. McLaughlin, Simone J. Stanger, Amanda L. Anderson, Kate Hutcheon, Kiralee Church, Bettina P. Mihalas, Sonika Tyagi, Janet E. Holt, Andrew L. Eamens, Brett Nixon

**Affiliations:** 1 Priority Research Centre for Reproduction, School of Environmental and Life Sciences, Discipline of Biological Sciences, University of Newcastle, Callaghan, New South Wales 2308, Australia; 2 School of Biological Sciences, University of Auckland, Auckland 1142, New Zealand; 3 School of Environmental and Life Sciences, Discipline of Biological Sciences, University of Newcastle, Callaghan, New South Wales 2308, Australia; 4 Australian Genome Research Facility Ltd, The Walter and Eliza Hall Institute, Parkville, Victoria 3052, Australia; 5 School of Biomedical Sciences and Pharmacy, Faculty of Health and Medicine, University of Newcastle, Callaghan, New South Wales 2308, Australia

## Abstract

Recent evidence has shown that the sperm epigenome is vulnerable to dynamic modifications arising from a variety of paternal environment exposures and that this legacy can serve as an important determinant of intergenerational inheritance. It has been postulated that such exchange is communicated to maturing spermatozoa via the transfer of small non-protein-coding RNAs (sRNAs) in a mechanism mediated by epididymosomes; small membrane bound vesicles released by the soma of the male reproductive tract (epididymis). Here we confirm that mouse epididymosomes encapsulate an impressive cargo of >350 microRNAs (miRNAs), a developmentally important sRNA class, the majority (~60%) of which are also represented by the miRNA signature of spermatozoa. This includes >50 miRNAs that were found exclusively in epididymal sperm and epididymosomes, but not in the surrounding soma. We also documented substantial changes in the epididymosome miRNA cargo, including significant fold changes in almost half of the miRNAs along the length of the epididymis. Finally, we provide the first direct evidence for the transfer of several prominent miRNA species between mouse epididymosomes and spermatozoa to afford novel insight into a mechanism of intercellular communication by which the sRNA payload of sperm can be selectively modified during their post-testicular maturation.

Spermatozoa released from the germinal epithelium of the testes are functionally immature, lacking both motility and the potential to fertilise an ovum^1^. These attributes are progressively acquired as they traverse the several meters of the epididymal tubule, a highly specialised region of the extragonadal male reproductive tract^2^. Since spermatozoa are both transcriptionally and translationally quiescent, this functional transformation is driven exclusively by the luminal microenvironment that they encounter during their prolonged residence within this ductal system^3^. This environment is, in turn, created by the combined secretory and absorptive of the lining epithelium and characterised by considerable segment-segment variation^4^. A central component of epididymal soma-spermatozoa intercellular communication are epididymosomes, a heterogeneous population of small membrane bound vesicles that are released from the epididymal epithelium via an apocrine secretory mechanism^5^,^6^,^7^,^8^. Similar to the exosome population documented in other somatic tissues and bodily fluids, epididymosomes are able to relay a complex macromolecular cargo to recipient cells^9^,^10^,^11^. As such, they have been implicated as holding major roles in the promotion of sperm maturation by virtue of their ability to exert paracrine control of the epididymal epithelium^11^ and via the direct contact they have with the maturing sperm population^5^,^6^,^7^,^8^. Traditional paradigms have held that the key elements of this transfer are proteins that contribute to the acquisition of various functional attributes necessary to reach the site of fertilisation and engage in interactions with the oocyte^6^,^7^. However, recent evidence suggests that such exchange may extend to additional molecules such as small non-protein-coding RNAs (sRNAs)^12^.

These data are of considerable interest in view of the potential role sRNAs play in altering the sperm epigenome, and further, manipulation of this specific cargo may mediate direct consequences in offspring if the paternal linage encounters environmental insult(s)^13^,^14^. In this context, a growing body of recent evidence has shown that the sperm epigenome is modified by exposure to a wide range of environmental stressors, including chronic stress^15^,^16^, paternal diet^12^,^17^,^18^ and cigarette smoke^19^. Since these epigenome perturbations are inheritable, they are capable of influencing the developmental trajectory and/or health of offspring. Such findings encourage detailed spatial and temporal documentation of these mechanism(s), to identify where and when such epigenetic information is relayed to developing gametes. In this regard, many studies have put forward the hypothesis that such alterations coincide with sperm passage through the epididymis^14^,^20^, a time in which spermatozoa are known to be particularly vulnerable to a range of insults having left the relative protection afforded by the testicular germinal epithelium^21^. This agrees with our own evidence, that is; the sperm miRNA profile is dynamically modified as the cells migrate through the epididymis^22^. Furthermore, and in keeping with established paradigms of intercellular communication in other tissues^23^,^24^, it has been repeatedly suggested that epididymosomes may serve as a vehicle to mediate soma-spermatozoa intercellular communication^14^,^20^. Notwithstanding the growing acceptance of this mechanism, and the elegant demonstration that bovine epididymosomes can deliver miRNA cargo to epididymal epithelial cells during *in vitro* co-culture^11^, direct transfer of miRNAs from epididymosomes to spermatozoa has not yet been substantiated by conclusive experimental evidence.

While epididymosomes are beginning to emerge as attractive candidate vectors to facilitate the transfer of epigenetic information to spermatozoa, there remain fundamental challenges to this field of extracellular vesicle research. Not the least, is the development of robust and reproducible methods for epididymosome isolation and characterisation, particularly in the context of established laboratory models such as the rodents, where the scale of epididymal fluid recovery remains a particular challenge. Here, in an effort to address this limitation, we report the validation of a simple method of epididymosome isolation from differing segments of the mouse epididymis and the profiling of the miRNA content of these specialised extracellular vesicles. Additionally, we provide the first direct evidence for the selective transfer of miRNA cargo between epididymosomes and mouse spermatozoa.

## Results

### Isolation of epididymosomes from the mouse epididymis

Our initial studies focused on evaluating the suitability of a number of protocols for purification of epididymosomes, including: (i) ultracentrifugation, (ii) OptiPrep density-based separation, and (iii) a commercial total exosome isolation kit. Among these, the greatest consistency and highest recovery of enriched populations of epididymosomes was achieved using the OptiPrep density-based separation. This technique had the added advantage that it was scalable, thus enabling us to generate sufficient material for detailed endpoint analysis of epididymosome cargo. This technique was therefore used for all subsequent analyses to establish the miRNA cargo of epididymosomes derived from the different segments of the mouse epididymis.

**Figure 1:**
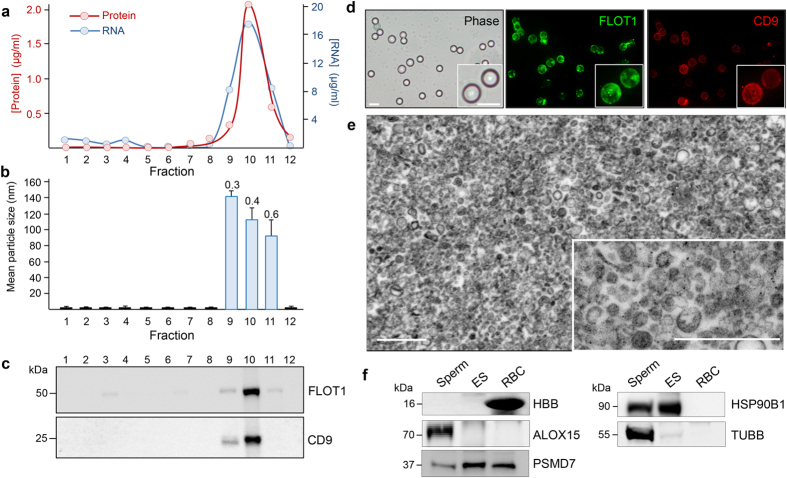
Assessment of epididymosome purity. A suite of assays were employed to assess the purity of epididymosomes isolated by OptiPrep density gradient fractionation. Briefly, twelve equal fractions were recovered after ultracentrifugation of the gradient and an aliquot of each prepared for (**a**) quantitative assessment of both protein and RNA content and (**b**) size heterogeneity. The latter was accomplished via measurement of mean particle size using dynamic light scattering. These data are reported as particle size (columns) and a polydispersity index value (numbers above columns), whereby the lower the value the more homogenous the preparation. (**c**) Immunoblot analyses were performed to determine distribution of the epididymosome markers flotillin 1 (FLOT1) and CD9 within each fraction. (**d**) The same markers were also used to dual-label epididymosomes bound to aldehyde/sulphate latex beads (FLOT1 green, CD9 red). Scale bar = 5 μm. (**e**) Epididymosome preparations were also assessed via transmission electron microscopy to confirm the size and heterogeneity of the isolated populations. Scale bar = 500 nm. (**f**) Epididymosome (ES) preparations (pooled fractions 9 and 10) were resolved by SDS-PAGE alongside cell lysates prepared from spermatozoa (sperm) and red blood cells (RBC) and immunoblotted with either anti-haemoglobin (HBB) or anti-arachidonate 15-lipoxygenase (ALOX15) antibodies to control for blood and/or cytoplasmic droplet contamination, respectively. Immunoblots were also probed with antibodies against known epididymosome protein cargo (26S proteasome non-ATPase regulatory subunit 7, PSMD7; heat shock protein 90kDa beta member 1, HSP90B1; beta tubulin, TUBB).

A suite of confirmation assays were employed to assess the enrichment and purity of the epididymosome isolation via the OptiPrep protocol. The relatively low-density epididymosomes readily partitioned away from other contaminants in a translucent layer corresponding to fractions 9–11 of the OptiPrep density gradient. Quantitative analysis of these fractions confirmed they possessed the highest concentration of both total protein and RNA of each of the twelve fractions (Fig. 1a). Importantly, a consistent profile of protein and RNA enrichment (both peaking in fraction 10) was obtained irrespective of the epididymal segment from which the epididymosomes were isolated. Furthermore, we failed to observe any significant variation in the physical properties of the epididymosomes in fractions 9–11. In this context, sizing analysis confirmed the purity of these preparations revealing a heterogeneous population of particles of approximately 50 to 150 nm (Fig. 1b). Immunoblot assessment of the abundance of extracellular vesicle markers, FLOT1 and CD9, confirmed that both assessed markers partitioned almost exclusively into fractions 9 and 10 (Fig. 1c). Epididymosomes from these fractions also readily adhered to aldehyde/sulphate latex beads permitting their visualisation via labelling with both anti-FLOT1 and anti-CD9 antibodies (Fig. 1d). In contrast, no such labelling was detected in beads incubated with either anti-PLZF (an irrelevant protein control) or secondary antibody only negative controls (Supplementary Figure S1). Finally, ultrastructural analysis confirmed both the purity and sizing of the epididymosomes preparations (Fig. 1e). Taken together, these analyses confirm the successful isolation of epididymosomes from the intraluminal milieu of the mouse epididymis. Owing to the highest expression of extracellular vesicle markers (Fig. 1c) and the lowest size heterogeneity (Fig. 1b), fractions 9 and 10 were selected as the focus of our remaining studies. These fractions were pooled and, prior to next generation sequencing, each biological replicate was assessed via immunoblotting for a variety of markers to ensure: (i) minimal contamination with either blood (haemoglobin, HBB) and/or cytoplasmic droplets (arachidonate 15-lipoxygenase, ALOX15)^25^, and (ii) enrichment of known epididymosome protein cargo (26S proteasome non-ATPase regulatory subunit 7, PSMD7; heat shock protein 90kDa beta member 1, HSP90B1; beta tubulin, TUBB)^10^. As shown in Fig. 1f, neither HBB nor ALOX15 were detected within the epididymosome preparations. In contrast, the epididymosome preparations did contain PSMD7, HSP90B1, and TUBB.

#### Characterisation of the miRNA signature of mouse epididymosomes

**Figure 2:**
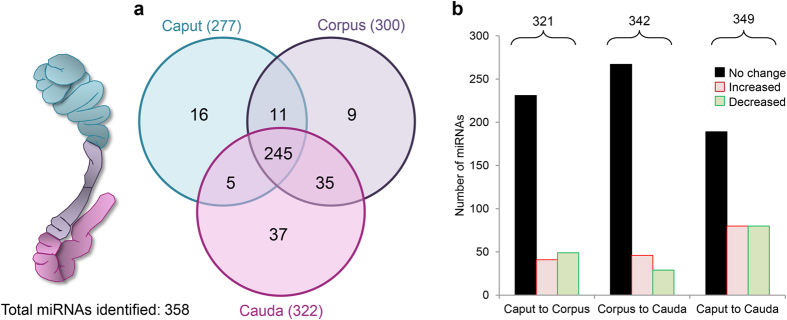
Evaluation of epididymosome miRNA signatures throughout the epididymis. (**a**) Venn diagram illustrating the total number of mature miRNAs identified in adult mouse epididymosomes by next generation sequencing and their distribution throughout the caput, corpus and caudal epididymal segments. (**b**) Graphical representation of the proportion of miRNAs identifying those that were either present at equivalent levels (unchanged) or alternatively, were significantly up- or down-regulated (increased or decreased, respectively) (threshold = ±≥2-fold change and false discovery rate of <0.05) across different epididymal segments. Post normalisation average counts of ≥10 reads / million across each of three biological replicates (n = 9–12 mice/replicate) were used as a detection sensitivity threshold for the positive identification of epididymosome miRNAs reported in this study.

Next generation sequencing was employed to elucidate the miRNA cargo present in mouse epididymosomes. This approach identified a total of 358 miRNAs across the three epididymal segments surveyed (Supplementary Table S1). Among the 358 miRNAs detected, the highest read scores were returned for miRNAs: *miR-10b-5p, miR-10a-5p, miR-143*-*3p, miR-141-3p* and *miR-30a-5p*. Via normalisation based on total read counts for each library, a gradient of increasing miRNA profile complexity was noted between epididymosomes sampled from the proximal (caput) versus the distal (cauda) epididymal segments. Specifically, the overall number of epididymosome-borne miRNAs increased from 277 in the caput, to 322 in the cauda epididymis (Fig. 2a). Of these, a majority (~68%) were detected in each epididymosome fraction derived from the three epididymal segments examined. Further, profiling also revealed that only ~17% of detected miRNAs were unique to any one epididymal segment (Fig. 2a). Notwithstanding this striking conservation of epididymosome miRNA cargo, the profile for this sRNA species was far from static with substantial variations recorded in the relative abundance of numerous miRNAs between epididymal segments. For instance, of the 321 miRNAs present in both the caput and corpus epididymosomes, 28% exhibited significant differences in abundance (13% up- and 15% down-regulated) between the two segments (fold change of ≥±2; FDR <0.05; Fig. 2b and Supplementary Table S2). A similar number of differentially accumulating miRNAs were identified among the 342 miRNAs detected in corpus and cauda epididymosome fractions (13% up- and 9% down-regulated; Fig. 2b and Supplementary Table S2). This trend was even more pronounced when considered across the entire length of the tract with almost half (~46%) of the 349 miRNAs identified in the caput and cauda segments showing significant fold changes (Fig. 2b and Supplementary Table S2).

**Figure 3:**
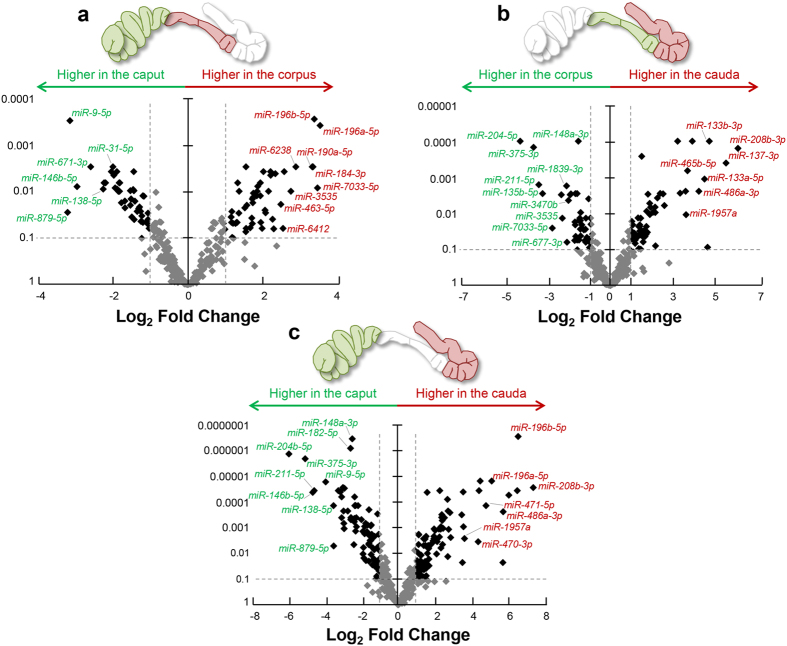
Volcano plots depicting fold changes associated with differentially accumulated epididymosome miRNAs. Volcano plots were constructed to demonstrate the fold change (x-axis) and false discovery rate (y-axis) of miRNAs that were determined to be differentially accumulated in epididymosomes isolated from the (**a**) caput/corpus, (**b**) corpus/cauda, and (**c**) caput/cauda epididymis. Thresholds denoting significant increases or decreases in miRNA accumulation are depicted by dotted lines (threshold = ±≥2 fold change and false discovery rate of <0.05).

Illustrative of the magnitude of these variations, several miRNAs were determined to have accumulation differences of greater than 64-fold between the different epididymal segments examined (Fig. 3 and Supplementary Table S2). Among the numerous examples of these, miRNAs *miR-208b-3p* and *miR-196b-5p* appeared to be selectively accumulating into cauda epididymosomes (compared to caput epididymosomes), such that these two miRNAs were 147 and 84 fold more abundant, respectively (Fig. 3c). Conversely, miRNAs *miR-204b-5p* and *miR-375-3p* returned an opposing accumulation profile for epididymosomes sampled from the same two epididymal segments (i.e. caput and cauda) with their respective levels reduced ~55 and 32 fold, respectively (Fig. 3c).

**Figure 4:**
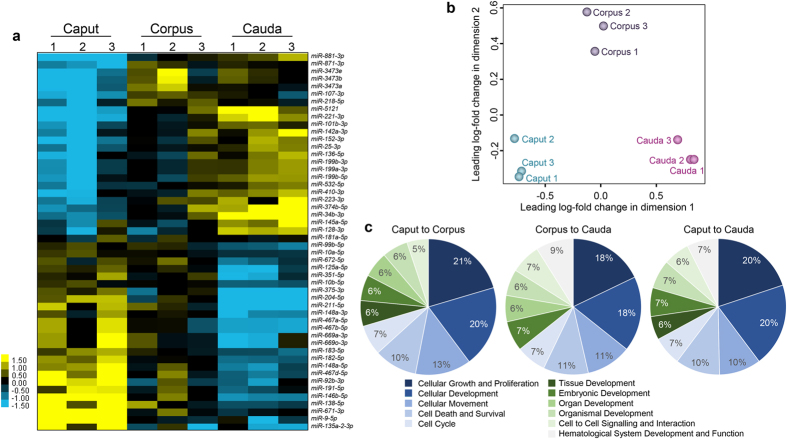
Profiling of variability in epididymosome miRNA abundance across biological replicates and determination of the putative biological functions regulated by differentially accumulating miRNAs. (**a**) Hierarchical clustering of the 50 miRNAs that exhibited the highest fold changes between epididymal segments was performed to assess consistency among the three biological replicates subjected to next generation sequencing. Cells within the matrix depict the relative abundance of a single miRNA, with yellow and blue colouring representing the accumulation (log_2_ fold change), above and below the median accumulation of this miRNA, in all biological replicates respectively. (**b**) Multi-dimension scaling analysis of normalised miRNA data based on leading log fold changes of the miRNAs showing the relationship between biological replicates. Three distinct populations are shown, corresponding to epididymosomes sampled from each epididymal segment. (**c**) Biological functions of differentially accumulating epididymosome miRNAs were predicted through interrogation of Ingenuity Pathway Analysis software. A majority of the experimentally validated target genes mapped to the broad categories of regulating cellular growth and proliferation, cell development, and cell death and survival.

Notably, we recorded consistent results across all biological replicates, both in terms of the overall number of miRNA reads that were detected by next generation sequencing (Supplementary Table S1) and the relative fold change between segments as reported by hierarchal clustering analysis (Fig. 4a), and multi-dimensional scaling (MDS; Fig. 4b). MDS clearly illustrated tight clustering of the biological replicates representing epididymosomes sampled from the three epididymis segments analysed, thus enabling clear differentiation of each population of epididymosomes on the basis of their epididymal segment of origin (Fig. 4b). Despite this clustering, an *in silico* analysis of the key biological pathways potentially targeted by differentially accumulating miRNAs revealed considerable conservation, with a majority centred on regulation of the broad categories of cellular growth and proliferation, cellular development, and cell death and survival (Fig. 4c). Such categories accord with those previously documented for the miRNA cargo identified in mouse epididymal spermatozoa^22^. In this context, it was also notable that 6–7% of all differentially accumulated epididymosome miRNAs mapped to the category of embryonic development (Fig. 4c).

### Validation of epididymosome miRNA abundance

**Figure 5:**
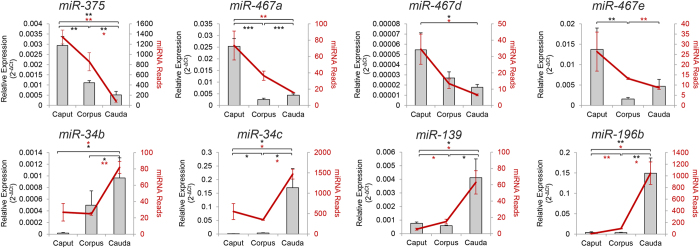
RT-qPCR validation of the abundance of differentially accumulating epididymosome miRNAs. Next generation sequence data were validated via the targeted RT-qPCR amplification of differentially accumulating miRNAs. Candidate miRNAs included representatives with the highest abundance (according to sequencing data) in epididymosomes from the proximal (caput: *miR-375, miR-467a, miR-467d* and *miR-467e*) or distal epididymis (cauda: *miR-34b, miR-34c, miR-139* and *miR-196b*). cDNA generation and RT-qPCR validations were performed in triplicate using three pools of biological samples (n = 6–9 mice per sample) differing from those used for next generation sequence analyses. Expression levels of target miRNAs were normalised against the U6 small nuclear RNA control (determined to uniformly accumulate across samples by sequencing). Values are shown as an average ± SEM. *P < 0.05, **P < 0.01, ***P < 0.001. NGS reads are represented as dark red line graphs while the relative abundance (2^−ΔCt^) of each miRNA assessed by RT-qPCR is represented by the grey columns.

To confirm the next generation sequencing data, the levels of eight miRNAs were quantified by RT-qPCR. The selected miRNAs fall into one of two groupings: i) high accumulation in the caput (*miR-375, miR -467a, miR-467d* and *miR-467e*), or ii) high accumulation in the cauda epididymis (*miR-34b, miR-34c, miR-139* and *miR-196b*). These analyses confirmed the sequencing data with each of the eight assessed miRNAs returning differential accumulation profiles across the three analysed segments of the epididymis from which epididymosomes were harvested (Fig. 5). Both assessment strategies were also highly suggestive that miRNA incorporation into epididymosomes is selective. For instance, *miR-34c* and *miR-467e* were found to be predominantly accumulated into epididymosomes in the cauda and caput epididymis, respectively. In contrast, the expression of *miR-34c* was some 2-fold higher in the epithelium of the caput epididymis versus that of the cauda, and in the case of *miR-467e*, we failed to detect this miRNA in the epithelium of any segment of the epididymis (Supplementary Table S3)^26^. We did however note close similarities in the trends of miRNA abundance within both epididymosomes and spermatozoa sampled from equivalent segments (Supplementary Table S3)^22^. In this context, *miR34c* and *miR-467e* were again most abundant in cauda and caput spermatozoa, respectively^22^.

### Comparison of the miRNA profile of epididymal epithelial cells, spermatozoa and epididymosomes

To build on these observations, we profiled two previously generated sequencing datasets^22^,^26^ to compare the miRNA signatures of mouse epididymal epithelial cells (accession number GSE70197), spermatozoa (accession number GSE70198), and epididymosomes (this study). This analysis revealed a number of striking results, including; only ~46% (190) of the 412 miRNAs identified across the three individual experiments performed were represented in each dataset (Supplementary Table S3). Further, 164 of the total number of miRNAs detected in epididymosomes (n = 358) were either absent, or below the established detection sensitivity, in the epididymal soma. These included several highly abundant epididymosome miRNAs, namely *miR-21a-5p* (20,283 reads) and *miR-6240* (2,427 reads; Supplementary Table S1). Such data accord with independent evidence that a portion of miRNAs are selectively packaged into epididymosomes thus precluding their accumulation in the parent epithelial cells from which they originate^11^. In contrast to the epithelial cell-epididymosome comparison, this approach also revealed substantial overlap in the miRNA profiles of spermatozoa and epididymosomes (Supplementary Table S3). More specifically, spermatozoa share 242 (82%) of their total pool (n = 295) of miRNAs with that of epididymosomes. In addition, 52 miRNAs detected in both our spermatozoa/epididymosome datasets were further determined to be absent in our epididymal epithelial cell miRNA profiles.

**Figure 6:**
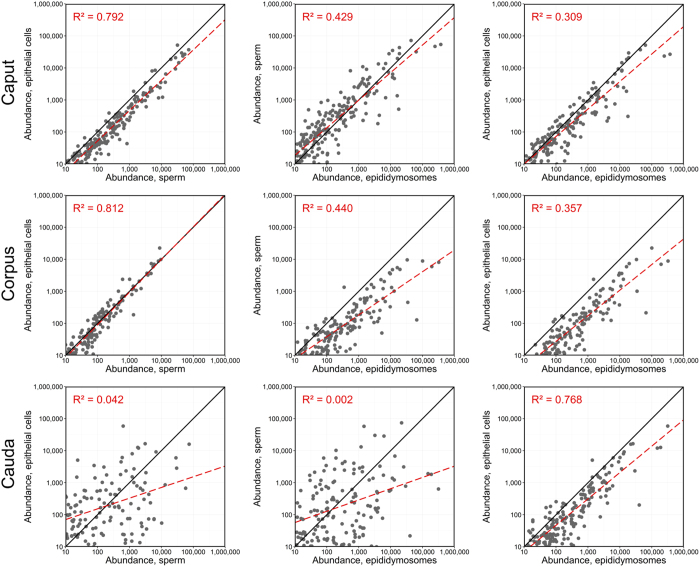
Linear regression modelling of miRNA accumulation in sperm, epithelial cell and epididymosome populations. Scatterplots of genome-wide miRNA accumulation between epididymal sperm, epithelial cell and epididymosome datasets revealed substantial correlation in the miRNA signature between cell types. Linear regression modelling (red dashed line) and calculation of R^2^ values was performed in R.

Linear regression analysis was also undertaken to gauge similarities in the relative abundance of miRNAs that were detected in mouse epididymal epithelial cell, spermatozoa and epididymosome datasets (Fig. 6). A strongest positive correlation was detected in comparisons of the abundance of miRNAs in caput-corpus epithelial cells and spermatozoa (R^2^ = 0.792 and 0.812, respectively). Similar positive correlations were also observed when comparing miRNA abundance between sperm-epididymosomes and between epithelial cells-epididymosomes however, the correlation coefficient was lower among these datasets. A notable exception to this trend was that the miRNA profile of cauda sperm was markedly different to that of cauda epithelial cells and epididymosomes (R^2^ = 0.042 and 0.002, respectively), while comparison of miRNA abundance between cauda epithelial cells-epididymosomes revealed a strong positive correlation (R^2^ = 0.768). These observations parallel our previous analyses, which demonstrated that the miRNA signature of cauda spermatozoa is highly dissimilar to that of caput and corpus spermatozoa^22^. Further, comparison of miRNA abundance between epithelial cells-epididymosomes revealed that epididymosomes are substantially enriched in multiple miRNAs, particularly in the corpus and cauda epididymal segments. The observed miRNA enrichment within epididymosomes relative to parent epididymal cells provides ancillary evidence for the highly selective compartmentalisation and export of epithelium-generated miRNAs.

### miRNAs are directly transferred from epididymosomes to mouse spermatozoa

**Figure 7:**
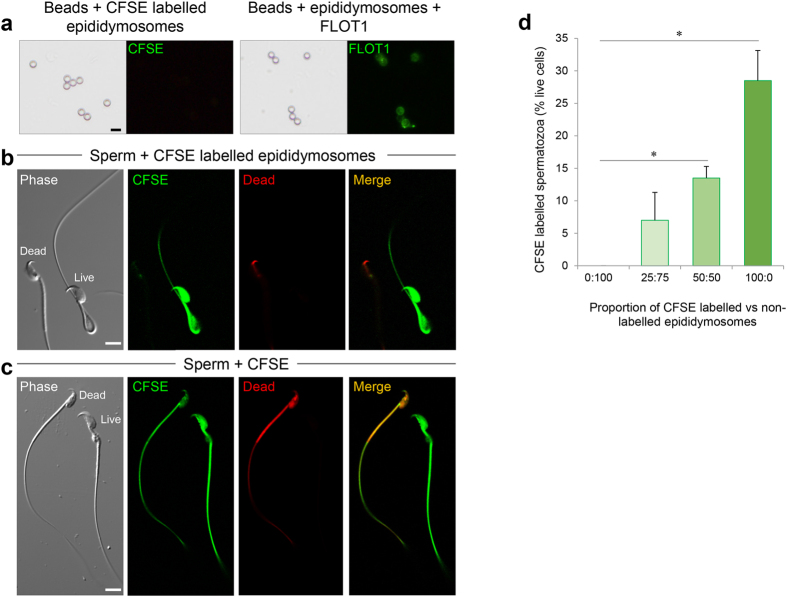
Assessment of sperm-epididymosome interaction. The ability of epididymosomes to interact and deliver encapsulated cargo to spermatozoa was investigated after co-incubation. Prior to analysis, preparations of caput and corpus epididymosomes were pooled and labelled with carboxyfluorescein diacetate succinimidyl ester (CFSE). (**a**) CFSE labelled epididymosomes were concentrated by binding to sulphate/aldehyde latex beads and examined by fluorescence microscopy, revealing no labelling. The efficacy of epididymosome bead binding was assessed by subsequent labelling with FLOT1. (**b**) CFSE labelled epididymosomes were washed prior to co-incubation with caput spermatozoa. After washing, spermatozoa were subsequently counterstained with live/dead stain (red = dead cells) and assessed via confocal microscopy. (**c**) Controls included caput sperm incubated directly with CFSE, caput sperm co-incubated with unlabelled epididymosomes and caput sperm incubated in media alone. The latter two treatments yielded no fluorescence labelling and consequently are not shown. Scale bars = 5 μm. (**d**) An additional competition assay was also performed as outlined for (**b**) with the exception that spermatozoa were co-incubated with varying proportions of non-labelled versus CFSE labelled epididymosomes. The efficacy of CFSE dye internalisation was subsequently recorded and are plotted as a percentage of live CFSE labelled spermatozoa.

We next sought to investigate the potential for epididymosome-mediated transfer of miRNAs to spermatozoa. Although such a mode of intercellular trafficking has been confirmed for protein cargo^27^, it remains to be proven as a mechanism for transfer of miRNAs to maturing mouse spermatozoa. For the purpose of this study, we employed a co-incubation strategy originally developed for investigating of the delivery of epididymosome protein cargo to spermatozoa in the bovine model^27^. The utility of this protocol was initially assessed by labelling of epididymosomes with carboxyfluorescein diacetate succinimidyl ester (CFSE). As anticipated based on an absence of esterase activity, the labelled epididymosomes did not yield any fluorescent signal following adherence to aldehyde/sulphate latex beads (Fig. 7a). In contrast, approximately 30% of the live sperm population were strongly labelled following co-incubation with CFSE loaded epididymosomes (Fig. 7b). Notably, this labelling appeared highly selective such that it was exclusively detected in the head and mid-piece of the flagellum and further appeared restricted to live spermatozoa, with no staining observed in any of the dead cells. To control for the possibility of non-specific labelling due to the presence of unbound CFSE, spermatozoa were also directly labelled with CFSE, revealing a distinct pattern of labelling that was present in all cells. Specifically, the dye readily labelled the entire sperm cell, including both the head and mid/principal piece of the flagellum (Fig. 7c). In contrast to epididymosome-mediated labelling, direct labelling of the sperm population also yielded CFSE fluorescence, albeit less intense, in dead cells (Fig. 7c). The selectivity of CFSE internalisation was further evidenced via the use of a competition assay whereby spermatozoa were incubated with varying proportions of CFSE labelled (0%, 25%, 50%, 100%) versus non-labelled (100%, 75%, 50%, 0%) epididymosomes (Fig. 7d). As shown, this experiment established a strong dose-response relationship, whereby the more CFSE labelled epididymosomes present in the co-incubation suspension, the greater the percentage of spermatozoa that stained with CFSE.

**Figure 8:**
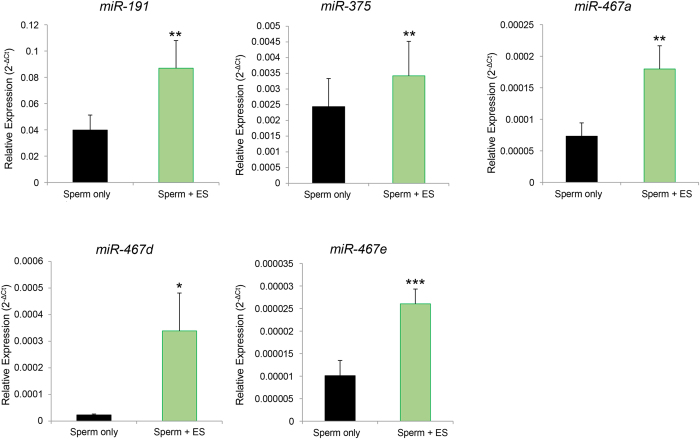
Examination of miRNA transfer to sperm after co-incubation with epididymosomes. The ability of sperm-epididymosome interaction to facilitate transfer of miRNA cargo to sperm was directly assessed by RT-qPCR amplification of candidate miRNAs (*miR-191, miR-375, miR-467a, miR-467d*, and *miR-467e*) from sperm that were incubated in either media alone (sperm only) or epididymosomes (sperm + ES). Analyses were performed in triplicate using three biological samples (n = 3 mice/sample). Values are shown as average abundance ± SEM. *P < 0.05, **P < 0.01, ***P < 0.001.

Having established the suitability of a co-incubation protocol to track spermatozoa-epididymosome interactions, we next investigated whether such interactions facilitated the transfer of miRNA cargo to spermatozoa. Five miRNAs (*miR-191, miR-375, miR-467a, miR-467d, miR-467e*) were selected for inclusion in this analysis based on their high abundance in caput epididymosomes. As illustrated in Fig. 8, this strategy proved effective in demonstrating significant accumulation of each of the five target miRNAs into spermatozoa. Since, only ~30% of the live sperm population were able to consistently internalise the CSFE dye during epididymosome co-incubation (Fig. 7d), it is considered likely that miRNA transfer efficacy would be considerably higher if this analysis was restricted to the live cell only population. Nevertheless, the data presented in Fig. 8 provide the first evidence that identifies mouse spermatozoa as recipients of epididymosome-mediated transfer of miRNA cargo.

## Discussion

This study extends on the previous work that has identified the miRNA class of sRNA as an additional and potentially developmentally important tier of regulation in the male reproductive tract^11^,^22^,^26^,^28^,^29^,^30^. Through development of a tractable protocol for the isolation of mouse epididymosomes, here we provide novel insight into the complexity of the segment specific miRNA profiles of these extracellular vesicles as well as exploring their capacity to deliver this important regulatory cargo to maturing spermatozoa. Our data indicate that, in addition to the more widely reported role as transporters of protein^7^,^31^,^32^ and lipid cargo^9^, epididymosomes are also carriers of miRNA, a developmentally important class of regulatory RNA. Further, many of the profiled miRNA were determined to be at considerably enriched levels compared to parent cells (the epididymal epithelial cells). Indeed, almost a third of the miRNAs detected in the epididymosome fraction were not present in our equivalent profiling of epididymal epithelial cell miRNAs^26^. While we cannot entirely discount the possibility that such differences may, in part, reflect either: (i) greater depth of sequence coverage achieved in our current analysis, (ii) profiling of a subset of epididymosomes originating from a non-surveyed epithelial cell population upstream of the caput epididymis, or (iii) contamination of our samples with vesicles released from ruptured cytoplasmic droplets; the data presented here does nevertheless accord with independent evidence that the epididymosome miRNA signature diverges from that of the epithelial cells from which they originate^11^. Taken together, the observations made to date present strong evidence that the packaging of the molecular cargo into epididymosomes is a highly selective, rather than stochastic process. Such a model draws support from a wealth of extracellular vesicle based studies^33^,^34^,^35^, which have shown that abundant RNA species in extracellular vesicles can remain virtually undetectable in the parent cell^36^. However, the precise sorting mechanism responsible for discriminating the molecular payload of these vesicles from that of their parent cells remains poorly understood. While the presence of consensus exomotifs (e.g. GGAG and CCCU)^37^ have been reported within the 3′ half of the mature sRNA sequence of miRNAs selectively incorporated into some exosome populations, similar motifs were only detected in the 3′ half of a small portion (25/358; ~7%) of the epididymosome-borne miRNAs identified here (Supplementary Figure S2). Such findings do not preclude the possibility that alternative exomotifs may be present among these miRNAs or that non canonical pathways^38^ may be adopted for the extracellular export of miRNA in the epididymis.

The epididymis may represent an interesting model to address the question of selective vesicle packaging considering the substantive segment-segment variation in epididymosome miRNA profiles reported here. Indeed, we identified marked changes in epididymosome miRNA profiles between epididymal segments, including an apparent gradient of increasing profile complexity between the proximal and distal epididymal segments. This finding mirrors that of the sperm miRNA profile, that is; progressive modification of the miRNA profile as the sperm descend through the proximal epididymal segments (caput and corpus) before undergoing extensive changes coincident with their prolonged residence in the distal (cauda) epididymis^22^. These miRNA profiles do however, contrast with well established paradigms of epididymal sperm maturation that attribute the majority of functional changes to the proximal segments (distal caput/proximal corpus)^39^. From these data we infer that the modification of the sperm miRNA profile is not strictly tied to the functional maturation of these cells. Irrespective, they identify the epididymis as an important site in establishment of the sperm epigenome, and since these cells are incapable of *de novo* transcription, they also firmly implicate epididymosomes as a conduit for the transfer of such developmentally important regulatory information. Consistent with this hypothesis, we identified substantial overlap in the miRNA signature of both spermatozoa and epididymosomes with as many as 82% of sperm-borne miRNAs also being detected in epididymosomes. Prominent among these were members of the *let7, miR-30, miR-465, miR-466, miR-467*, and *miR-669* clusters.

We further exploited a co-incubation strategy originally developed in the bovine model^27^ to provide proof-of-concept that mouse epididymosomes can directly interact with homologous spermatozoa. Moreover, we demonstrate that this is a productive interaction leading to an apparent uptake, and significant accumulation, of several prominent epididymosome miRNAs. While we have yet to explore the full extent of miRNA transfer facilitated during this interaction, we did make the striking observation that it was highly selective. In this context, an encapsulated tracer dye (CFSE) was exclusively delivered from epididymosomes to the sperm head and mid-piece of the flagellum. Given the transcriptionally inert state of the mature spermatozoon, it is considered unlikely the uptake of miRNA into these intracellular domains would have any direct impact on the functional profile of these cells. Such restricted deposition would however, ideally position the miRNAs for entry into the oocyte cytosol at the time of fertilisation, thus enhancing the prospect that these miRNAs could serve as mediators of the epigenetic regulation of the resultant embryo. However, proof of an epigenetic regulatory role for epididymosome delivered miRNAs requires further evidence demonstrating that sperm-borne miRNAs control transcription homeostasis in fertilised oocytes, zygotes and two-cell embryos^40^. It is also notable that the sperm domains labelled with CFSE here perfectly align with the distribution of proteins trafficked to bovine spermatozoa via epididymosomes, which are also found to preferentially localise to the acrosomal and mid-piece domains^27^. The mechanism(s) by which such selective recognition and uptake of epididymosome cargo may be conferred have yet to be fully elucidated, but could conceivably involve complementary ligands/receptors furnished on the surface of the epididymosomes and the recipient spermatozoa^41^. In this context, previous work has shown that epididymosomes contain a variety of candidate adhesion molecules, including tetraspanins (CD9), integrins, and milk fat globule-epidermal growth factor 8 protein^42^. A similar repertoire of ligands have been documented on a variety of extracellular vesicles suggesting that they may be a universal feature to help target these entities and ensure selectivity in their uptake among the hundreds of cell types that they may encounter^35^.

As an additional tier of specificity, it was also noted that epididymosomes appeared to exclusively interact with live cells; we consistently failed to detect any fluorescent dye labelling of dead or moribund cells. Importantly, such selectivity was not artefactual as illustrated by the strong fluorescent signal generated throughout the entire spermatozoon (both live and dead cells) upon direct incubation with free CSFC. An important precedent for these findings has been provided by the work of Sullivan and colleagues who have shown that epididymosomes constitute a heterogeneous pool that can be subdivided into at least two populations on the basis of size and the presence/absence of CD9^32^,^43^. The smaller of these (~10 to 100 nm) bear the CD9 antigen and bind preferentially to live spermatozoa, whereas the larger CD9 negative sub-population possess higher affinity for dead cells^6^. While the scale of epididymosome recovery from mice precluded the possibility of exploring such heterogeneity, the characteristics of the epididymosomes isolated here (i.e. CD9 positive, diameter of 50 to 150 nm) suggest that our isolation protocol may have been biased toward the former population. In any case, the ability of epididymosomes to actively bind live cells supports the concept that this interaction is tightly regulated and raises the intriguing possibility that the epididymis is able to discriminate cell quality and restrict its investment to the processing of viable cells^6^.

Taken together, this work substantiates the growing consensus that the epididymis serves as a key staging point for establishment of the sperm epigenome^14^. Importantly, this epigenome may be altered by a range of environmental insults^44^. The relatively high degree of overlap documented among reported epididymosome miRNA profiles (Supplementary Tables S4 and S5)^11^,^45^ strongly suggests that this mode of intercellular communication may be highly conserved across mammalian species. Indeed, notwithstanding limitations imposed by the use of different methodology for miRNA identification (next generation sequencing versus microarray approaches), our mouse epididymosome dataset comprised as many as 88% of the miRNAs that have previously been documented in bovine epididymosomes^11^ (Supplementary Table S5). Further, and while beyond the scope of the present study, it is likely that epididymosome function may also extend to the horizontal transfer of additional species of regulatory non-protein-coding RNA, including (but not limited to) transfer RNA fragments (tRFs), piwi-interacting RNAs (piRNAs), and other subclasses of small-interfering RNA (siRNA), thus potentially contributing to pronounced epigenetic alterations (such as metabolic/reproductive disruption and adverse behavioural symptoms) on subsequent generations^15^,^16^,^46^. Indeed, the recent studies of Rando and colleagues (2016) present compelling evidence that dietary perturbations can alter the profile of tRFs delivered to sperm via epididymosomes^12^. Further exploration of this pathway for information transfer is thus likely to prove a productive avenue for future research, particularly in the context of addressing pertinent questions, such as: how do epididymal soma respond to environmental cues to alter the molecular cargo of epididymosomes^14^? This is particularly perplexing in view of the fact that many of the stressors linked to changes in the sperm epigenome^15^,^17^,^19^ occur at sites distal from the male reproductive tract and that this tissue apparently lacks the innervation^3^ required for conveying extrinsic stress-induced neuronal factors directly to the sperm. This has encouraged speculation that the heterogeneous population of epididymosomes that sperm encounter may include contributions, albeit minor, from somatic cells that lay beyond that of the male reproductive tract^13^. While the validity of such a model awaits further scrutiny, it is notable that genetic markers originating from distal somatic cells have been detected in epididymal mouse spermatozoa and crude preparations of plasma extracellular vesicles^47^.

Notably, although a focus for our work has been epididymosome-sperm interactions, this does not discount the possibility that these extracellular vesicles hold a fundamental role in relaying regulatory information to enforce the strict control of epididymal epithelial cell function. Certainly, extracellular vesicles are replete in most biological fluids and have been conclusively shown to convey miRNA cargo to recipient cells where they act to initiate RNA regulatory pathways^48^. Further support for this form of paracrine regulation has been afforded by the elegant study of Sullivan and colleagues who have shown that epididymosomes can bind, and subsequently transfer miRNAs, directly to cultured epididymal epithelial cells^11^. Such a mechanism could underpin the control of at least a portion of the >17,000 genes that are known to be expressed in the mouse epididymis^49^ and conceivably account (at least partially) for the segment-specific patterns of gene expression and/or protein abundance that characterise this ductal system^3^. Indeed, comprehensive transcriptomic analyses have led to the demarcation of 6 unique transcriptional units within the mouse epididymis^49^. Thus, in dividing the epididymis into three broad anatomical segments, we may have inadvertently overlooked some of the subtlety associated with epididymosome miRNA profiles. Despite this, an analysis of the key biological pathways potentially targeted by differentially accumulating miRNAs revealed a majority centred on regulation of cellular growth and proliferation, cellular development, and cell death and survival, as might be expected of molecules involved in the maintenance of epididymal homeostasis. Given that epididymosomes also feature among the constituents of seminal fluid that are delivered to the female tract at the time of ejaculation, it must also be considered that they could exert similar regulatory control within the female reproductive with potential implications for conditioning of the periconceptual environment^50^,^51^.

In summary, this study reports the comprehensive mapping of the miRNA profile of mouse epididymosomes under normal physiological conditions. In so doing, we have revealed a complex profile that is discrete from that of their parent cells. These data support the selective processing and packaging of the macromolecular cargo of epididymosomes, and demonstrate that this selective packaging further extends to their downstream interactions with spermatozoa. The significance of such findings lie in their validation of a widely promulgated model of intercellular communication between the epididymal soma and maturing germ cells. In addition to potential implications for epigenetic mechanisms of inheritance, these data identify epididymosomes as a potential conduit for modulating the environments of both the male and female reproductive tracts through the delivery of RNA silencing substrates. Further research is now warranted to explore the extent of the role epididymosomes play in such phenomena.

## Methods

### Reagents

All reagents used were of research grade and, unless specified, were obtained from Sigma Aldrich (St. Louis, Mo, USA) or ThermoFisher Scientific (Waltham, MA, USA).

### Ethics statement

All experimental procedures were carried out with the approval of the University of Newcastle’s Animal Care and Ethics Committee (approval number A-2013-322), in accordance with relevant national and international guidelines. Inbred Swiss mice were housed under a controlled lighting regime (16L: 8D) at 21–22 °C and supplied with food and water *ad libitum*. Prior to dissection, animals were euthanized via CO_2_ inhalation.

### Epididymosome isolation

Immediately after adult male mice (8 weeks old) were euthanised, their vasculature was perfused with pre-warmed PBS to minimise the possibility of blood contamination. The epididymides were then removed, carefully separated from fat and overlying connective tissue and dissected into three anatomical segments corresponding to the caput, corpus and cauda. Luminal fluid was aspirated from each segment by placing the tissue in a 500 μl droplet of modified Biggers, Whitten, and Whittingham media (BWW; pH 7.4, osmolarity 300 mOsm/kg^52^,^53^) and making multiple incisions with a razor blade. The tissue was then subjected to mild agitation and the medium subsequently filtered through 70 μm membranes. This suspension was then divided into three equal aliquots and prepared for epididymosome isolation using either differential centrifugation^54^, a commercial exosome isolation protocol (Total Exosome Isolation kit, Invitrogen) or OptiPrep (Sigma-Aldrich) density gradients^55^. Of these, the OptiPrep density gradients proved most suitable and were therefore adopted for use in all subsequent experiments. This protocol involved sequential centrifugation of the epididymal fluid suspensions with increasing velocity (500 × g, 2,000 × g, 4,000 × g, 8,000 × g, 17,000 × g) to eliminate all cellular debris before layering the supernatant onto a discontinuous OptiPrep gradient (40%, 20%, 10%, and 5%), created by diluting 60% OptiPrep with a solution of 0.25 M sucrose, 10 mM Tris. The gradient was ultracentrifuged (100,000 × g, 18 h, 4 °C), after which twelve equivalent fractions were collected, diluted in PBS and subjected to a final ultracentrifugation spin (100,000 × g, 3 h, 4 °C). Notably, approximately 30–40% of the sperm cells recovered during the initial centrifugation step (500 × g), had shed their cytoplasmic droplet. While, it is considered unlikely that these relatively large entities (0.5 to 2.0 μm in diameter) would be co-purified with epididymosomes (~50 to 150 nm in diameter), it is acknowledged that they contain spherical vesicles (~60 to 100 nm in diameter)^56^ that could be released if cytoplasmic droplets were ruptured. Thus, to control for this possibility, all isolated epididymosome preparations were immunoblotted with the antibodies against the cytoplasmic droplet marker, ALOX15^25^.

### Epididymosome characterisation

Isolated epididymosome fractions were characterised on the basis of their purity, particle size and overall homogeneity. Each sample was initially analysed on a Zetasizer Nano ZS (Malvern Instruments, Malvern, United Kingdom) to determine mean particle size in addition to the amount of variation within a sample. The latter is reported in the form of a polydispersity index (PDI), whereby low PDI values reflect highly monodisperse preparations, and values >1 indicate that the sample returned a varied size distribution. Each sample was analysed a minimum of 10 times, with 10 cycles per analysis. Epididymosome size and purity was further assessed via conventional transmission electron microscopy, whereby pelleted epididymosomes were sequentially fixed in 2.5% glutaraldehyde and 2% osmium tetroxide before being embedded in Spurr’s resin as previously described^57^. Embedded resin blocks were sectioned with a diamond knife and micrographs were captured on a transmission electron microscope at 80 kV. Epididymosomes were further visualised via binding to 4 μm aldehyde/sulphate latex beads (ThermoFisher Scientific) and fluorescent labelling of recognised extracellular vesicle surface markers, including CD9 and flotillin 1 (FLOT1)^58^ using established protocols for extracellular vesicle analysis^59^. As a final complementary validation strategy, isolated populations of epididymosomes were prepared for immunoblotting with a suite of antibodies recommended for experimental validation of extracellular vesicles, including anti-CD9, anti-FLOT1, anti-PSMD7, anti-HSP90B1, and anti-TUBB antibodies.

### RNA extraction and next-generation sequencing of the small RNA fraction

Total RNA was extracted from purified epididymosomes using a Direct-zol RNA MiniPrep Kit (Zymo Research Corporation, Irvine, CA, USA) according to manufacturers’ instructions before being incubated with 1% DNase (Promega) to eliminate genomic DNA contamination^53^. Total RNA from each epididymal segment (caput, corpus, cauda) was pooled from a minimum of nine animals to generate a single biological replicate. Three such biological replicates were subjected to Illumina TruSeq small RNA sample preparation protocol as per the manufacturers’ instructions (Illumina Inc. San Diego, CA, USA) at the Australian Genome Research Facility (AGRF, Melbourne, VIC, Australia). The miRNA libraries generated from the three biological replicates were analysed in triplicate and sequenced using an Illumina Hiseq-2000 RNA-seq platform as 50 base-pair (bp) single end chemistry at AGRF as previously described^22^,^26^,^53^. Briefly, the sequence reads from all samples were analysed for quality control on an Agilent 2100 Bioanalyser (Agilent Technologies, Santa Clara, CA, USA) and screened for the presence of contaminants by matching against the contaminant database (containing PhiX, ChrM, rDNA and Illumina small RNA adaptor sequences) using cutadapt^60^ and bowtie aligner. The cleaned sequence reads were then processed through the quantification modules miRDEEP2 ver2.0.0.7 pipeline for known miRNA expression profiling^61^.

miRNA read counts were normalised as per library size, and a normalised count value of >10 counts per million (CPM) was used as the detection threshold for miRNA presence per library. The edgeR^62^ and limma Bioconductor package were used to perform sample diagnostics and differential expression analysis with a data filter set to ≥2-fold difference and false discovery rate (FDR) of 0.05. A multi dimension scaling (MDS) plot^63^ was generated to visualise the relationship between the set of samples in each biological replicate. For this purpose, the leading log-fold change was plotted for dimensions 1 and 2 using all miRNA counts, with samples displaying similar expression profiles clustering together. All data discussed in this publication have been deposited in NCBI’s Gene Expression Omnibus and are accessible through GEO accession number GSE79500 (http://www.ncbi.nlm.nih.gov/geo/query/acc.cgi?acc=GSE79500).

### Real time PCR validation of miRNA read data

Validation of the next generation sequencing generated miRNA profiles was conducted using quantitative real-time PCR (RT-qPCR) with (non-locked nucleic acid modified) TaqMan miRNA assay reagents to detect and amplify only mature forms of each miRNA under analysis in accordance with the manufacturer’s instructions (ThermoFisher Scientific). The miRNAs selected for analysis were *miR-375* (assay ID 000564), *miR-139-5p* (assay ID 002689), *miR-191-5p* (assay ID 002299), *miR*-*196b-5p* (assay ID 002215), *miR-151* (assay ID 001190), *miR-34b-5p* (assay ID 002617), *miR-34c-5p* (assay ID 000428), *miR-467a-5p* (assay ID 001826), *miR467d* (assay ID 002518) and *miR-467e* (assay ID 002568). RT-qPCR was performed using a Light Cycler 96 SW 1.1 (Roche, Castle Hill, Australia). RT-qPCR data was normalised against the U6 small nuclear RNA (snRNA; assay ID 001973) as this endogenous snRNA was identified as evenly abundant across each epididymis segment, and relative abundance was calculated using the 2^−ΔCt^ method^64^. All sRNA RT-qPCR analyses were performed in triplicate using pooled biological samples (6–9 mice/sample). However, due to limitations in generating the volume of epididymosome material required for total RNA extraction for sRNA sequencing, the cDNA used for these RT-qPCR analyses was synthesised from a separate pool of animals to that used for sequencing.

### Transfer of epididymosome miRNA cargo to spermatozoa

Epididymal spermatozoa were isolated as previously described^22^ prior to co-incubation with purified epididymosomes using methodology optimised for the *in vitro* transfer of proteins between bovine epididymosomes and spermatozoa^27^. The suitability of this protocol was initially assessed by preloading epididymosomes with carboxyfluorescein diacetate succinimidyl ester (CFSE), a non-fluorescent membrane permeant dye. Upon entry into a cell, the acetate groups of CFSE are rapidly removed by intracellular esterases yielding a highly fluorescent, non-membrane permeant carboxyfluorescein label that is capable of forming stable conjugates with primary amines. For this study, freshly isolated epididymosomes were pooled from the caput epididymal segment of six animals and resuspended in modified BWW (pH 6.5) supplemented with 1 mM ZnCl_2_ (Zn-BWW)^27^ before being split into two equal samples. The samples were either labelled with CFSE (1 μM) for 30 min at 37 °C, or treated with an equivalent volume of DMSO prior to being washed in PBS and subjected to ultracentrifugation (100,000 × g, 3 h, 4 °C). The resultant epididymosome pellets were resuspended in Zn-BWW and co-incubated with caput spermatozoa (2 × 10^6^) for 3 h at 37 °C in 5% CO_2_ with gentle agitation^27^. Following incubation, spermatozoa were pelleted by centrifugation (400 × g, 3 min), resuspended in Zn-BWW, and their viability assessed by co-labelling with Live/Dead fixable far red dead cell stain (ThermoFisher Scientific) for 15 min at 37 °C. The sperm suspensions were then washed three times by centrifugation (400 × g, 3 min) in Zn-BWW before mounting and visualisation by confocal microscopy. Additional controls for this experiment included incubation of labelled epididymosomes with aldehyde/sulphate beads and direct CFSE (1 μM) labelling of populations of caput spermatozoa (2 × 10^6^) for 3 h at 37 °C in 5% CO_2_. In addition, a competition experiment was performed whereby spermatozoa were incubated with varying proportions of non-labelled versus CFSE labelled epididymosomes in order to assess the dye internalisation efficacy achieved in this assay.

To assess epididymosome-mediated transfer of miRNAs to spermatozoa, freshly purified caput and corpus epididymosomes were pooled from three animals and resuspended with 2 × 10^6^ caput spermatozoa in 250 μl of Zn-BWW or an equivalent volume of Zn-BWW medium only (control). Epididymosomes and spermatozoa were then co-incubated as described above before the spermatozoa were pelleted by centrifugation (400 × g, 3 min). The cells were then resuspended in pre-warmed PBS and washed three times to remove any unbound or peripherally adherent epididymosomes, before being processed for total RNA extraction. The relative level of candidate miRNAs was subsequently quantified by RT-qPCR to determine the efficacy of miRNA transfer.

### *In silico* analysis of identified miRNAs and target prediction

*In silico* analysis of miRNA profiles was undertaken using a suite of techniques^53^. Briefly, miRNAs were log transformed, subject to hierarchical median gene clustering (Cluster3, Stanford University, Palo Alto, CA, USA) and examined using heatmaps (Java Treeview, Stanford University) to ensure consistency among biological replicates, and via volcano plots to visualise trends associated with differentially accumulating miRNA in the epididymosomes of each epididymal segment. Ingenuity Pathway Analysis (IPA) software (v8.8, Ingenuity Systems, Redwood City, CA, USA) was utilised to further investigate miRNAs determined to have significant fold changes in accumulation between epididymal segments. To identify biological pathways that may be influenced by differentially accumulating miRNAs, gene targets of these miRNAs were predicted using experimentally validated filters.

### Statistical analysis

JMP Software (v12.2.0) was used to perform multivariate correlation analyses and Student T-tests to determine statistical significance with a significance threshold of *P* < 0.05. Linear regression modelling was performed in R. All experiments were performed in triplicate and all data are expressed as mean ± SEM.

## Additional Information

****Accession codes:**** All data discussed in this publication have been deposited in NCBI’s Gene Expression Omnibus and are accessible through GEO accession number GSE79500 (http://www.ncbi.nlm.nih.gov/geo/query/acc.cgi?acc=GSE79500).

**How to cite this article**: Reilly, J. N. *et al*. Characterisation of mouse epididymosomes reveals a complex profile of microRNAs and a potential mechanism for modification of the sperm epigenome. *Sci. Rep.* **6**, 31794; doi: 10.1038/srep31794 (2016).

## Supplementary Material

Supplementary Information
