# Supplementary material for: Characterisation of mouse epididymosomes reveals a complex profile of microRNAs and a potential mechanism for modification of the sperm epigenome
Source: Sci Rep. 2016 Aug 23;6:31794. doi: 10.1038/srep31794 (PMC4994100; doi:10.1038/srep31794)
Supplement: Supplementary Information [file srep31794-s1.pdf]

Manuscript Title: Characterisation of mouse epididymosomes reveals a complex profile of microRNAs and a potential mechanism for modification of the sperm epigenome

Authors: Jackson N. Reilly, Eileen A. McLaughlin, Simone J. Stanger, Amanda L. Anderson, Kate Hutcheon, Kiralee Church, Bettina P. Mihalas, Sonika Tyagi, Janet E. Holt, Andrew L. Eamens, Brett Nixon

SUPPLEMENTARY INFORMATION FILES

Supplementary Figure S1: Assessment of the specificity of epididymosome binding to aldehyde/sulphate latex beads

Supplementary Figure S2: Identification of the presence of consensus exomotifs among epididymosome miRNAs

Table S1: Normalised abundance reads of miRNAs identified by next generation sequencing within mouse epididymosomes

Table S2: Fold changes of epididymosome miRNA abundance between epididymal segments

Table S3: Comparison of miRNAs identified in mouse epididymal spermatozoa, epithelial cells and epididymosomes

Table S4: Comparison of mouse epididymal miRNA profile reported by Nixon et al., 2015a,b and Reilly et al., 2016 versus Sharma et al., 2016

Table S5: Comparison of miRNAs identified in the epididymosomes of mice, humans and bulls

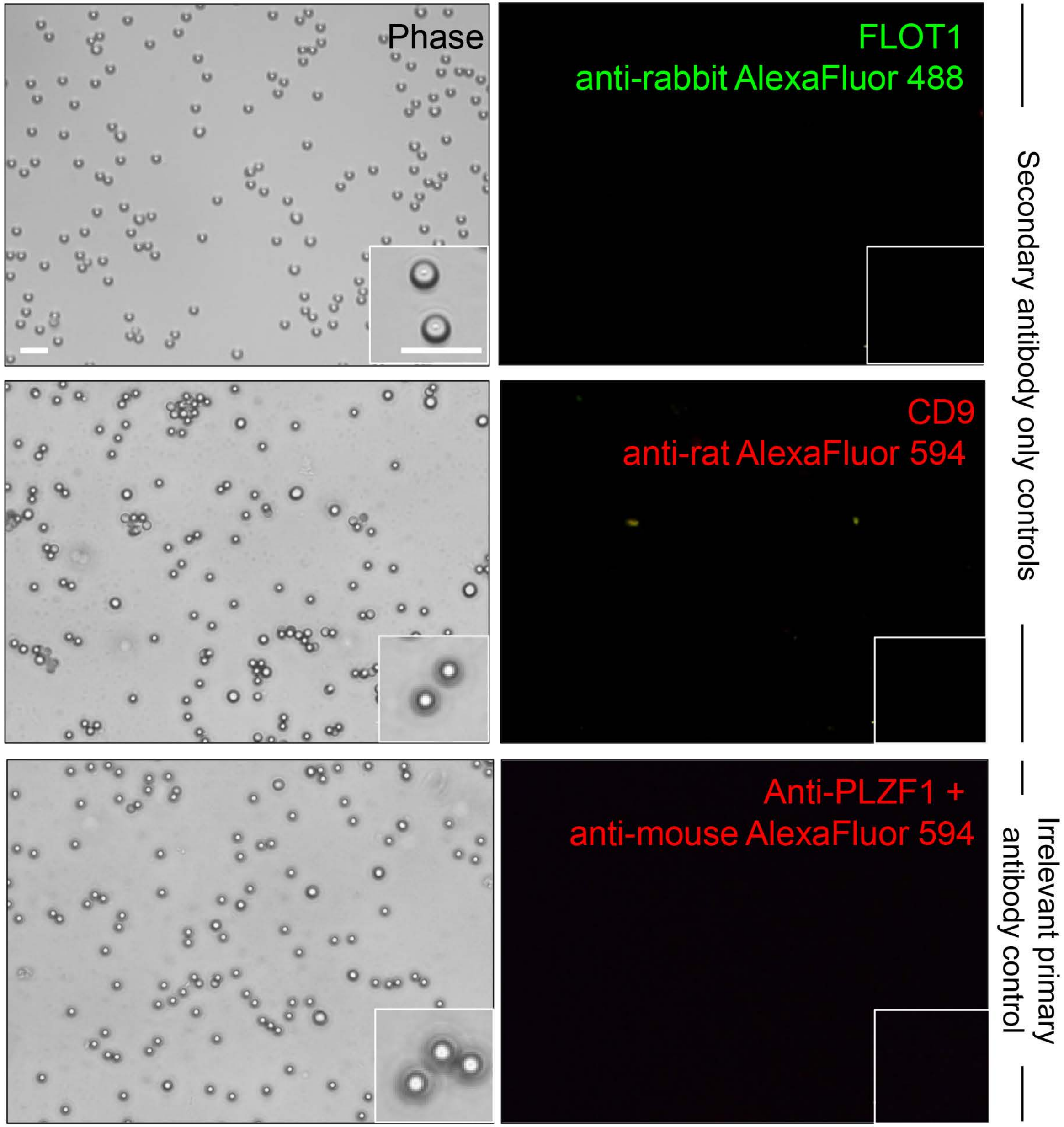

Supplementary Figure S1: Assessment of the specificity of epididymosome binding to aldehyde/sulphate latex beads. Epididymosomes were visualised via binding to 4 µm aldehyde/sulphate latex beads and fluorescent labelling of the recognised extracellular vesicle surface markers, CD9 and FLOT1 (see Fig. 1d). The specificity of labelling was established by incubation of epididymosome bound beads with either anti-rabbit AlexaFluor 488 alone (secondary antibody control for FLOT1), anti-rat AlexaFluor 594 alone (secondary antibody control for CD9), or a combination of anti-PLZF1 (irrelevant primary antibody control) and anti-mouse AlexaFluor 594.

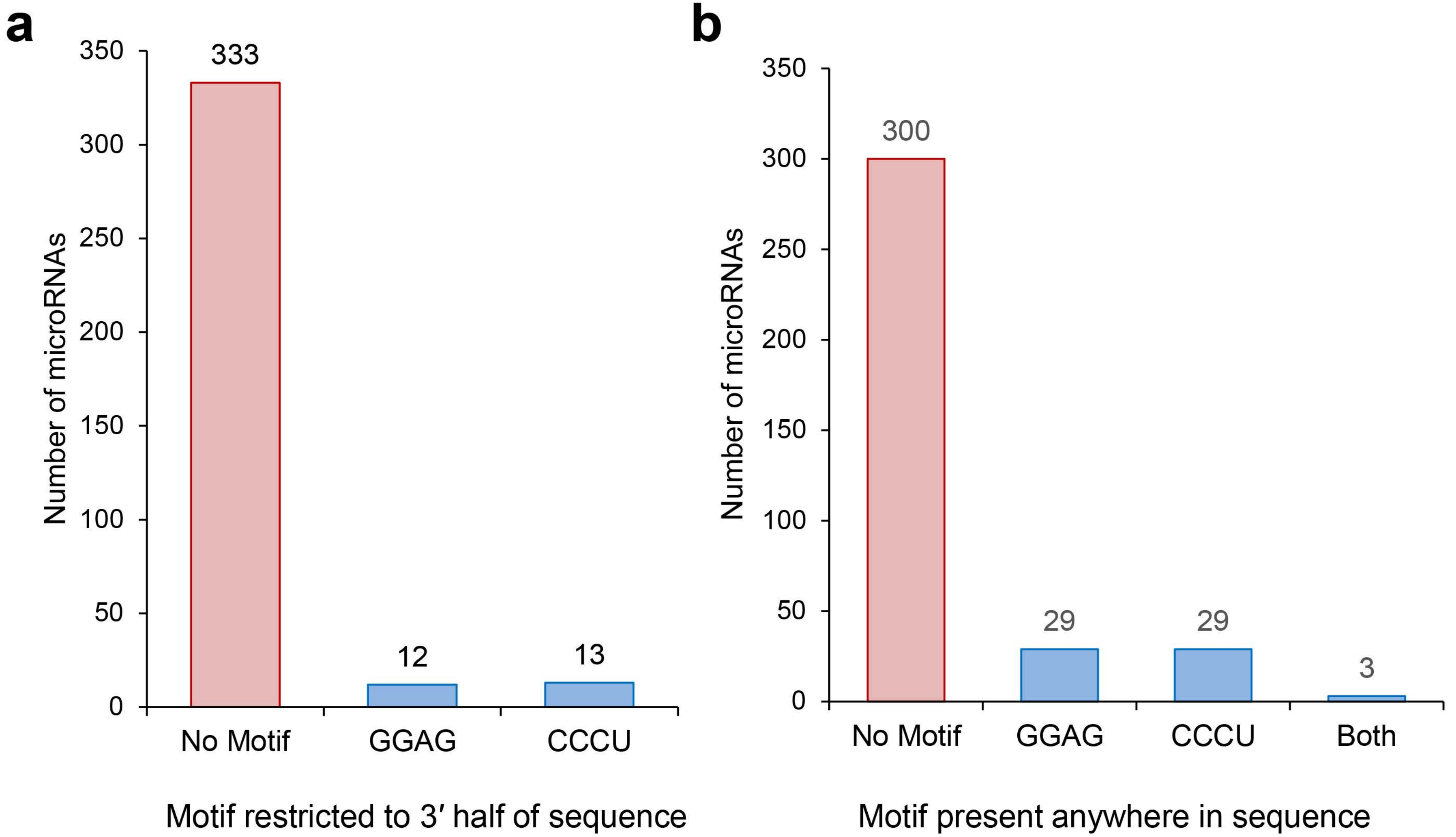

Supplementary Figure S2: Identification of the presence of consensus exomotifs among epididymosome miRNAs. The list of 358 unique miRNAs identified in this study were screened for the presence of the consensus exomotifs 'GGAG' and 'CCCU' reported by Villarroya-Beltri et al. [37] as playing a key role in the sorting of miRNAs into exosomes. (a) This analysis revealed that only 7% (25/358) of the miRNAs possessed either exomotif in the 3' half of their sequence. (b) Upon relaxation of the criterion for the exomotif to reside in the 3' half of the miRNA sequence, the number of miRNAs possessing either motif extended to 17% (61/358).

Table S1 : Normalised abundance reads of miRNAs identified by next generation sequencing within mouse epididymosomes

Total miRNAs identified in mouse epididymosomes = 358

miRNA Family (mmu)	Mature miRNA (mmu)	Caput			Corpus			Cauda			Average Normalised Reads [1,2]		
		Replicate 1	Replicate 2	Replicate 3	Replicate 1	Replicate 2	Replicate 3	Replicate 1	Replicate 2	Replicate 3	Caput	Corpus	Cauda
let-7	*let-7a-1-3p*	44.48727094	22.59976861	75.3544736	19.22354953	15.66547935	35.51638565	27.4850756	65.4587468	57.99806944	47.4805	23.46847	50.31396
	let-7a-5p	4197.81181	2339.74075	5880.133154	4846.596075	5588.659759	4452.463255	3861.403258	4981.273305	5045.323287	4139.229	4962.573	4629.333
	let-7b-3p	28.9211483	39.3985271	75.8276571	16.47732817	15.66547935	18.72682152	8.495387005	14.1903577	25.09857976	48.04911	16.95654	15.92811
	let-7b-5p	1660.887596	1307.281803	2751.088914	1385.549448	1991.8657	1321.209546	1199.348754	1124.242532	1007.165305	1906.419	1566.208	1110.252
	let-7c-2-3p	44.48727094	22.59976861	75.3544736	19.22354953	15.66547935	35.51638565	27.4850756	65.4587468	57.82848445	47.4805	23.46847	50.25744
	let-7c-5p	11285.17358	7358.943907	15850.70114	8382.598391	10589.08077	8746.071403	9060.080376	9064.892048	9274.264391	11498.27	9239.25	9133.079
	let-7d-3p	76.76928662	99.46315277	164.1946772	84.00206516	136.6813074	123.9844735	82.45522681	91.09294135	175.0117183	113.4757	114.8893	116.1866
	let-7d-5p	517.2198021	301.5316721	670.7376221	788.327073	883.5330356	586.3432394	905.0085803	882.5486981	741.2560279	496.4964	752.7344	842.9378
	let-7e-5p	655.1922527	369.4518323	977.3605351	519.682007	662.2581397	453.3182313	287.3439722	545.6421412	430.9154809	667.3349	545.0861	421.3005
	let-7f-2-3p	17.7772196	6.284427635	28.03612283	10.98488544	10.57419856	10.33203946	10.49430159	7.78180906	8.818419916	17.36592	10.63037	9.03151
	let-7f-5p	11333.64082	4080.64806	11235.15091	10951.6077	12711.36158	10580.65416	10214.45355	11381.58238	8649.343672	8883.147	11414.54	10081.79
	let-7g-5p	1903.046936	1044.786094	2337.644824	2504.230796	2764.957106	2221.388484	2233.287325	2461.798184	1691.779944	1761.826	2496.859	2128.955
	let-7i-3p	10.79015319	12.56885527	15.02357637	43.77799934	35.63896553	39.39090044	22.48778913	32.04274319	27.13359974	12.79419	39.60262	27.22138
	let-7i-5p	1678.31104	776.126813	1701.567894	2757.206246	3021.870967	3083.468027	3750.463498	4296.931862	3047.612006	1385.335	2954.182	3698.336
	let-7j	349.7955399	146.9589232	348.1447658	388.9941787	439.4166959	438.4659246	458.7508983	569.4453218	534.8710849	281.6331	422.2923	521.0224
miR-1	*miR-1a-3p*	3.537755144	1.208543776	5.678202092	3.553933526	2.349821903	5.16601973	10.49430159	25.17644108	10.1750999	3.474834	3.689925	15.28195
miR-7	*miR-7a-1-3p*	5.129744959	4.350757594	7.097752615	9.046376248	8.616013645	14.85230672	6.496472415	7.78180906	13.90596987	5.526085	10.83823	9.39475
miR-9	*miR-9-5p*	309.9957945	155.6604384	282.4905541	44.10108421	37.59715045	33.57912825	47.97395015	15.56361812	32.05156469	249.3823	38.42579	31.86304
miR-10	*miR-10a-3p*	71.37421003	43.38672156	106.3479933	53.47054532	72.06120503	61.99223676	76.95821169	59.50795164	73.2607193	73.70297	62.508	69.90896
	miR-10a-5p	248449.9105	279021.6983	207739.1529	208592.6359	197760.2281	177842.8122	149690.7179	150121.1673	145772.5512	245070.3	194731.9	148528.1
	miR-10b-3p	46.60992402	64.65709202	69.91286326	43.93954178	72.844479	52.30594977	55.46987986	43.02882657	33.40824468	60.39329	56.36332	43.96898
	miR-10b-5p	383539.7098	433973.93	296815.1201	323430.8899	315643.3519	325114.4112	181848.7561	183793.9707	199088.7181	371442.9	321396.2	188243.8
miR-15	*miR-15a-5p*	93.30829192	24.17087552	132.1364945	113.5643304	75.97757487	129.1504933	103.9435586	208.7355842	177.2163233	83.20522	106.2308	163.2985
	miR-15b-3p	6.721734774	5.921864503	6.50627323	7.269409485	10.96583555	11.62354439	8.495387005	16.02137159	14.75389486	6.383291	9.95293	13.09022
	miR-15b-5p	52.0934445	31.3012838	87.18406129	48.9473572	30.54768474	57.4719695	26.48561831	69.57852807	88.52336915	56.8596	45.65567	61.52917
miR-16	*miR-16-5p*	1506.553028	781.4444056	1770.179502	2093.913017	1609.628004	1757.092461	2857.448406	3210.682867	3597.406571	1352.726	1820.211	3221.846
miR-17	*miR-17-5p*	92.42385314	37.82742019	135.4487791	122.9337915	81.06885566	116.8811964	41.97720638	59.05019816	38.83496463	88.56668	106.9613	46.62079
miR-18	*miR-18a-5p*	11.76303585	7.130408279	17.50778978	67.68627943	22.71494506	98.15437488	62.46608092	190.8831987	179.7600983	12.13374	62.85187	144.3698
miR-19	*miR-19a-3p*	23.34918395	6.163573258	25.90679704	23.74673765	17.23202729	25.18434619	70.46173928	186.7634174	81.23121422	18.47318	22.05437	112.8188
	miR-19b-3p	268.6040593	85.8066081	280.2429324	253.4600774	209.5257864	341.6030547	445.7579534	1082.586966	568.4489146	211.5512	268.1963	698.9313
miR-20	*miR-20a-5p*	68.98622531	21.75378797	93.92692627	90.14067761	58.74554758	78.78180089	49.47313609	52.64164952	44.43126957	61.55565	75.88934	48.84869
miR-21	*miR-21a-3p*	43.24905664	31.42213818	94.40010978	56.86293641	74.41102693	78.13604842	39.47856314	59.50795164	74.61739929	56.3571	69.80334	57.86797
miR-21	*miR-21a-5p*	14550.78691	7233.738772	16003.06623	12809.50722	11794.93104	15738.27911	19568.8741	22358.51069	19223.47708	12595.86	13447.57	20383.62
miR-22	*miR-22-3p*	23594.08505	9332.375039	23369.46878	17955.60297	16608.54121	13921.77742	22514.77448	24206.91922	19482.77254	18765.31	16161.97	22068.16
	miR-22-5p	20.16520432	8.459806432	38.80104763	26.49295901	27.02295189	20.01832645	38.97883449	65.00099333	34.42575467	22.47535	24.51141	46.13519
miR-23	*miR-23a-3p*	299.1171974	254.6401736	454.0195756	382.5324813	392.0286208	344.1860645	657.6428999	927.866292	739.0514229	335.9256	372.9157	774.8535
	miR-23b-3p	438.3278623	488.1308311	762.180335	445.0494029	518.5273666	383.576965	676.6325885	1219.455255	1068.04632	562.8797	449.0512	988.0447
miR-24	*miR-24-2-5p*	106.5748737	54.62617868	103.9820758	106.2949209	106.1336226	82.01056322	55.9696085	43.48658004	142.6209836	88.39438	98.14637	80.69239
	miR-24-3p	301.0629628	258.9909312	485.0130954	592.6991867	541.2423117	545.660834	585.182246	796.9487984	509.7725051	348.3557	559.8674	630.6345
miR-25	*miR-25-3p*	383.227326	258.8700768	454.4927591	739.056631	647.7675713	780.7147317	869.5278464	1442.83895	1244.923473	365.5301	722.513	1185.763
miR-26	*miR-26a-5p*	15953.8606	11294.56671	21807.01684	18639.57363	21876.05864	18360.03412	20544.84415	27176.36601	29210.33763	16351.81	19625.22	25643.85
	miR-26b-5p	1779.31395	850.0896921	2295.886379	2471.114598	2560.130963	2406.073689	2217.296008	2289.225124	2396.236027	1641.763	2479.106	2300.919
miR-27	*miR-27a-3p*	736.2068455	380.3287263	887.2190769	861.3442527	827.1373099	615.4021004	1210.342784	1277.589946	968.8390957	667.9182	767.9612	1152.257
	miR-27b-3p	11931.07922	9057.673038	13764.43505	12388.20456	15246.42778	11038.49266	15695.97708	12780.93475	10479.1658	11584.4	12891.04	12985.36
	miR-27b-5p	11.05548483	6.04271888	11.82958769	12.27722491	15.27384237	10.97779193	28.4845329	18.31013896	18.82393482	9.642597	12.84295	21.87287
miR-28	*miR-28a-3p*	12.64747464	14.74423407	23.42258363	19.22354953	22.71494506	29.70461345	58.96798039	48.97962173	67.66441435	16.9381	23.88104	58.53734
	miR-28a-5p	63.06048544	28.0382156	81.74245095	58.80144561	71.27793106	61.3464843	95.94790029	105.7410525	75.29573928	57.61372	63.80862	92.32823
miR-29	*miR-29a-3p*	2197.29972	1118.748973	2832.94966	3393.844975	3916.369838	3343.060518	2912.918285	4337.214167	3895.536998	2049.666	3551.092	3715.223
	miR-29a-5p	8.932831739	1.450252531	12.18447532	9.69254598	7.441102693	5.16601973	12.99294483	13.27485075	16.44974484	7.52252	7.433223	14.23918
	miR-29b-3p	120.1067871	36.98143955	166.3240029	171.7196063	134.3314855	139.4825327	143.9218504	194.087473	155.5094435	107.8041	148.5112	164.5063
	miR-29c-3p	301.593626	110.2191924	374.0515628	269.2912358	323.1005117	220.201591	277.3493993	330.4980083	286.2594773	261.9548	270.8644	298.0356
miR-30	*miR-30a-3p*	133.8155883	141.3996218	214.9436084	225.1901516	283.5451763	317.0644609	594.6770903	638.5660964	569.8055945	163.3863	275.2666	601.0163
	miR-30a-5p	16682.46127	11360.43235	20163.05904	36429.91869	32325.71665	34580.69032	23266.86609	30122.92512	35281.98933	16068.65	34445.44	29557.26
	miR-30b-5p	1094.31611	469.7609658	1506.024809	1277.47756	900.7650628	1204.32835	954.9814451	1131.108835	1896.129867	1023.367	1127.524	1327.407
	miR-30c-2-3p	22.19941353	12.32714652	25.31531766	14.86190384	12.53238348	14.85230672	16.49104536	27.00745497	21.0285398	19.94729	14.0822	21.50901
	miR-30c-5p	2142.552959	1061.584853	2657.753467	2165.476314	1515.243491	1926.279607	2114.851636	2598.208719	3736.635854	1953.964	1869	2816.565
	miR-30d-3p	34.49311265	15.59021471	42.11333218	49.75506936	40.33860934	56.18046457	43.47639232	41.19781267	46.80545955	30.73222	48.75805	43.82655
	miR-30d-5p	7132.291258	5211.240762	8599.755364	9602.082217	10606.3128	8809.355145	4225.205713	6428.689791	7209.058281	6981.096	9672.583	5954.318
	miR-30e-3p	86.49811327	67.67845146	132.1364945	81.2558438	123.7572869	119.4642063	139.9240213	136.4105353	193.4964831	95.43769	108.1591	156.6103
	miR-30e-5p	4376.733776	2105.404112	5459.35472	5507.466168	5320.388426	5120.171305	3049.344206	4273.128681	4929.327148	3980.498	5316.009	4083.933
miR-31	*miR-31-3p*	3.537755144	2.296233175	3.312284554	1.292339464	1.174910952	8.394782062	15.49158807	30.66948277	23.23314478	3.048758	3.620677	23.13141
	miR-31-5p	70.22443961	34.80606075	81.26926744	21.96977089	21.54003411	18.72682152	111.4394884	88.80417398	53.92802948	62.09992	20.74554	84.7239
miR-32	*miR-32-5p*	76.0617356	20.54524419	81.86074683	115.8259245	75.97757487	101.3831372	36.9799199	56.30367732	37.47828464	59.48924	97.72888	43.58729
miR-34	*miR-34a-5p*	59.16895478	29.60932251	75.47276947	142.8035108	144.1224101	61.3464843	138.4248353	140.98807	73.0911343	54.75035	116.0908	117.5013
	miR-34b-3p	20.96119923	17.64473913	24.72383828	39.25481122	36.4222395	57.4719695	130.9289056	111.2340942	343.2400367	21.10993	44.38301	195.1343
	miR-34b-5p	37.14642901	5.438446992	38.09127237	24.06982252	22.32330808	28.41310852	81.45576952	69.12077459	94.79801409	26.89205	24.93541	81.79152
	miR-34c-3p	7.075510288	4.109048839	6.742864984	11.63105518	9.399287612	15.49805919	7.995658358	14.1903577	65.62939437	5.975808	12.17613	29.2718
	miR-34c-5p	797.5868972	163.878536	689.4283707	327.7695966	380.6711483	342.2488071	1355.264092	1709.709226	1341.247752	550.2979	350.2299	1468.74
miR-92	*miR-92a-3p*	164.0633948	107.4395417	182.2939463	894.6219939	1086.400993	301.5664018	152.4172374	181.2703758	164.1582784	151.2656	760.8631	165.9486
	miR-92b-3p	68.89778143	57.88924687	73.46173956	13.89264924	34.07241759	16.78956412	13.99240213	9.612822957	23.91148477	66.74959	21.58488	15.8389
miR-93	*miR-93-5p*	465.568577	223.2180354	555.7540297	688.6553919	847.89407	601.1955461	299.3374598	332.7867757	481.7909804	414.8469	712.5817	371.3051
miR-96	*miR-96-5p*	228.5389823	90.76163758	138.406176	226.3209486	95.95106104	74.26153362	169.9077401	152.8896604	88.35378415	152.5689	132.1778	137.0504
miR-98	*miR-98-5p*	517.5735776	337.4254223	556.9369885	708.3635687	646.5926603	622.5053775	408.7780335	950.2962123	791.2836024	470.6453	659.1539	716.7859
miR-99	*miR-99a-3p*	20.16520432	13.29398154	40.69378166	32.3084866	34.07241759	39.39090044	28.98426155	33.87375708	43.58334458	24.71766	35.25727	35.48045
	miR-99a-5p	719.8447279	885.7417335	843.80449	1324.486408	1229.740129	1076.469361	1717.067632	1356.781297	1279.010058	816.4637	1210.232	1450.953
	miR-99b-3p	19.54609717	17.16132162	33.71432492	24.06982252	46.21316409	31.64187085	15.99131672	22.88767371	25.60733475	23.47391	33.97495	21.49544
	miR-99b-5p	6881.464418	8117.305126	5918.342722	7289.440747	6899.468744	4746.92638	3614.037578	3461.074018	2853.267598	6972.371	6311.945	3309.46
miR-100	*miR-100-5p*	2322.71314	1568.206404	1383.351985	2247.216785	1393.052752	927.9462941	1570.647139	1504.177916	1048.71363	1758.091	1522.739	1374.513
miR-101	*miR-101a-3p*	1788.069894	613.9402382	2708.029214	4082.177282	3120.563487	4531.245056	3290.713143	3927.982561	3693.05251	1703.346	3911.329	3637.249
	miR-101a-5p	7.783061317	1.812815664	7.334344369	16.47732817	6.657828725	14.20655426	11.49375889	18.31013896	13.56679987	5.643407	12.44724	14.4569
	miR-101b-3p	235.7029365	87.01515188	365.8891473	549.5673571	478.1887573	584.405982	824.5522681	849.1326945	629.329929	229.5357	537.3874	767.6716
	miR-101c	18.04255123	3.263068195	17.03460628	44.58571151	54.04590377	80.71905829	75.9587544	108.0298199	27.64235474	12.78008	59.78356	70.54364
miR-103	*miR-103-3p*	830.5764639	354.1033264	985.8778382	1727.857863	1584.954874	1362.537704	1425.226102	1521.114795	950.3543309	723.5192	1558.45	1298.898
miR-106	*miR-106b-3p*	10.3479338	8.338952055	16.79801452	32.47002903	34.85569156	25.18434619	27.98480425	45.77534741	49.34923453	11.8283	30.83669	41.03646
	miR-106b-5p	36.17354635	12.08543776	50.27574769	89.81759275	52.87099282	63.2837417	71.96092522	79.19135102	72.5823793	32.84491	68.65744	74.57822
miR-107	*miR-107-3p*	212.6190842	107.9229592	248.4213415	724.517812	823.612577	696.7669111	623.6613519	558.9169919	335.0999568	189.6545	748.2991	505.8928
miR-125	*miR-125a-5p*	3694.389253	3206.629201	4232.389884	2813.90764	3338.705287	2679.872735	1010.451325	955.789254	1996.524186	3711.136	2944.162	1320.922
	miR-125b-1-3p	19.19232166	16.07363222	33.35943729	19.70817683	16.84039031	12.91504933	32.48236208	32.95825014	19.67185981	22.87513	16.48787	28.37082
	miR-125b-2-3p	175.7379868	194.5755479	331.1101595	276.7221877	286.6782722	278.9650654	285.3450576	346.9771334	414.126566	233.8079	280.7885	348.8163
	miR-125b-5p	2231.704389	1568.93153	2853.178255	2579.671113	2453.214067	2120.005347	1438.219047	1645.165986	2758.978339	2217.938	2384.297	1947.454
miR-126	*miR-126a-3p*	537.1196747	129.0724753	455.9123096	778.4729846	314.876135	342.2488071	682.6293323	774.5188782	449.7394157	374.0348	478.5326	635.6292
	miR-126a-5p	1327.719506	707.7232353	2215.208591	2330.249596	1405.976772	1972.773785	2364.216231	2682.893112	2234.112769	1416.884	1903	2427.074
miR-127	*miR-127-3p*	1086.267717	1291.449879	3119.107387	1613.970448	1830.119626	1884.951449	3267.225896	3099.448773	3103.914225	1832.275	1776.347	3156.863
	miR-127-5p	5.129744959	3.867340083	12.18447532	7.915579217	12.1407465	15.49805919	19.98914589	24.7186876	22.04604979	7.06052	11.85146	22.25129
miR-128	*miR-128-3p*	22.55318904	11.36031149	35.01557957	15.02344627	25.45640395	31.64187085	72.46065386	64.54323985	70.88652932	22.97636	24.04057	69.29681
miR-130	*miR-130a-3p*	378.5398004	215.3625009	538.24624	565.8831428	680.6650779	721.9512573	658.1426286	807.4771283	725.1454531	377.3828	656.1665	730.2551
	miR-130b-3p	7.959949074	6.284427635	13.01254646	6.946324619	10.57419856	7.749029595	6.996201063	14.1903577	14.58430986	9.085641	8.423184	11.92362
miR-132	*miR-132-3p*	7.340841924	8.58066081	13.72232172	16.1542433	23.88985601	19.37257399	15.99131672	21.51441328	20.18061481	9.881275	19.80556	19.22878
miR-133	*miR-133a-3p*	125.1480882	51.00054735	178.1535906	152.8191416	121.799102	107.1949094	2458.165216	3643.717654	1211.176058	118.1007	127.2711	2437.686
	miR-133a-5p	0.088443879	0	0.118295877	0.484627299	0.391636984	0	10.99403024	19.22564591	9.157589912	0.068913	0.292088	13.12576
	miR-133b-3p	6.633290895	3.02135944	12.65765883	10.17717328	6.657828725	7.749029595	236.8713788	440.8165956	153.6440085	7.437436	8.194677	277.1107
miR-134	*miR-134-5p*	3.714642901	4.109048839	11.35640418	6.300154887	9.007650628	14.85230672	9.994572947	13.73260422	21.70687979	6.393365	10.05337	15.14469
miR-135	*miR-135a-2-3p*	26.79849522	11.72287463	31.11181563	6.138612454	11.74910952	3.228762331	9.4948443	3.662027793	3.561284966	23.21106	7.038828	5.572719
	miR-135a-5p	288.503932	114.2073868	422.3162806	177.212049	139.4227662	138.8367803	85.4535987	123.1356845	93.7805041	275.0092	151.8239	100.7899
	miR-135b-5p	8.490612346	7.613825789	6.033089723	16.31578573	28.19786284	14.85230672	1.499185942	2.288767371	2.882944972	7.379176	19.78865	2.223633
miR-136	*miR-136-3p*	100.1184706	54.62617868	199.0919608	159.4423814	141.7725882	229.887878	203.8892881	259.5462198	308.135942	117.9455	177.0343	257.1905
	miR-136-5p	56.33875067	21.63293359	154.6127111	136.3418134	103.3921637	239.574165	159.9131672	252.6799177	327.9773869	77.52813	159.7694	246.8568
miR-137	*miR-137-3p*	0.265331636	0	0.236591754	0.646169732	0.391636984	0	34.48127667	37.53578488	18.31517982	0.167308	0.345936	30.11075
miR-138	*miR-138-5p*	113.5619401	23.80831239	124.3289666	33.7623685	17.23202729	14.85230672	10.99403024	10.98608338	12.37970488	87.23307	21.9489	11.45327
miR-139	*miR-139-5p*	5.306632716	3.504776951	7.807527876	11.79259761	12.1407465	20.66407892	72.96038251	34.78926403	81.06162922	5.539646	14.86581	62.93709
miR-140	*miR-140-3p*	172.9962265	121.8212126	262.7351426	235.3673249	270.6211558	282.1938278	285.3450576	240.7783274	379.5312264	185.8509	262.7274	301.8849
	miR-140-5p	12.02836749	2.054524419	15.85164751	11.46951274	7.441102693	7.103277129	25.48616101	19.68339939	11.19260989	9.97818	8.671298	18.78739
miR-141	*miR-141-3p*	19488.34332	15427.30301	18729.78619	66248.55177	58422.44707	72410.1613	32900.63496	38546.50455	49858.66786	17881.81	65693.72	40435.27
	miR-141-5p	72.08176106	21.27037046	69.6762715	99.02551143	83.81031454	74.26153362	47.4742215	91.09294135	54.94553947	54.3428	85.69912	64.50423
miR-142	*miR-142a-3p*	20.43053596	7.372117034	36.67172184	42.00103258	37.20551347	72.32427622	44.47584961	77.8180906	160.4274085	21.49146	50.51027	94.24045
	miR-142a-5p	169.9891347	177.6559351	402.3242774	366.5397805	585.1056539	944.0901057	158.9137099	293.4199769	1165.896864	249.9898	631.9118	539.4102
miR-143	*miR-143-3p*	51019.02826	49777.25934	88071.99014	91269.37459	87929.55234	120674.3464	352105.8065	306624.7914	287083.6603	62956.09	99957.76	315271.4
	miR-143-5p	13.17813791	3.263068195	17.62608566	10.17717328	10.18256158	4.520267264	50.97232203	19.22564591	24.58982476	11.35576	8.293334	31.59593
miR-144	*miR-144-3p*	1.415102058	0.604271888	11.59299594	5.815527588	5.482917774	13.56080179	10.49430159	18.76789244	10.1750999	4.537457	8.286416	13.14576
miR-145	*miR-145a-3p*	273.910692	105.5058716	208.3190392	341.0160761	164.0958962	229.2421255	1720.565733	1591.60883	716.4966181	195.9119	244.7847	1342.89
	miR-145a-5p	371.7296218	228.6564824	697.2358985	448.9264213	319.1841418	503.6869237	908.5066809	791.4557568	3850.766558	432.5407	423.9325	1850.243
	miR-145b	2.034209208	2.296233175	5.914793846	4.361645691	3.916369838	1.937257399	10.99403024	5.493041689	20.35019981	3.415079	3.405091	12.27909
miR-146	*miR-146a-5p*	124.7943127	436.0425944	179.4548453	297.3996191	687.3229067	473.9823103	180.9017703	195.9184869	218.0863079	246.7639	486.2349	198.3022
	miR-146b-5p	2566.376025	7031.670252	3679.35666	617.2536365	1066.819144	629.6086546	273.3515701	298.4552651	306.4400921	4425.801	771.2271	292.749
miR-148	*miR-148a-3p*	44275.27096	26327.15933	60668.15877	31852.7754	34939.11024	32236.60887	10850.60812	14811.52916	15082.04183	43756.86	33009.5	13581.39
	miR-148a-5p	178.9219664	195.0589655	348.9728369	93.04844141	150.7802388	103.9661471	50.97232203	43.48658004	72.07362431	240.9846	115.9316	55.51084

	miR-148b-3p	526.5064093	516.7733186	1080.869427	453.4496094	449.2076205	735.5120591	395.7850887	709.5178849	746.6827478	708.0497	546.0564	617.3286
	miR-148b-5p	17.7772196	7.613825789	19.75541144	19.86971926	16.05711634	16.78956412	14.99185942	18.31013896	16.95849984	15.04882	17.57213	16.7535
miR-149	*miR-149-5p*	106.5748737	36.98143955	130.8352399	36.83167472	24.281493	23.89284125	55.9696085	44.40208699	54.43678448	91.46385	28.33534	51.60283
miR-150	*miR-150-5p*	40.77262803	24.65429303	50.98552295	49.9166118	50.12953393	54.88895963	64.46499551	27.00745497	100.733489	38.80415	51.64504	64.06865
miR-151	*miR-151-3p*	529.8672767	785.3117457	1017.817725	611.7611938	893.7155971	756.8218905	744.0959559	875.2246425	812.1425572	777.6656	754.0996	810.4877
	miR-151-5p	617.2498288	636.4191525	828.6626178	712.0790447	753.1179199	479.7940825	728.6043678	698.074048	687.6671684	694.1105	648.3303	704.7819
miR-152	*miR-152-3p*	51.56278122	43.26586718	98.42216959	194.3355469	142.9474991	267.341521	191.3960719	393.6679877	316.954362	64.41694	201.5415	300.6728
	miR-152-5p	37.58864841	24.65429303	60.68578486	41.83949015	43.47170521	26.47585112	57.96852309	71.86729544	43.92251458	40.97624	37.26235	57.91944
miR-153	*miR-153-3p*	8.579056224	4.592466349	13.8406176	20.35434656	14.4905684	9.040534528	4.997286473	6.408548638	4.917964953	9.004047	14.62848	5.441267
miR-155	*miR-155-5p*	3.095535751	0.845980643	2.957396923	3.392391093	2.349821903	4.520267264	13.49267348	15.10586465	10.3446849	2.299638	3.420827	12.98107
miR-181	*miR-181a-1-3p*	26.88693909	15.46936033	29.69226511	31.339232	30.93932172	35.51638565	50.47259338	38.9090453	43.92251458	24.01619	32.59831	44.43472
	miR-181a-5p	5650.325628	4040.88697	8339.741027	4458.571151	7248.417297	5702.64003	5692.908751	4976.695771	6071.482112	6010.318	5803.209	5580.362
	miR-181b-5p	297.5252076	119.2832707	354.2961514	228.0979154	342.6823609	238.9284125	281.8469571	362.998505	218.4254779	257.0349	269.9029	287.757
	miR-181c-3p	265.0663042	132.0938347	284.1466964	210.9744175	197.7766768	159.5008592	149.9185942	157.4671951	172.1287734	227.1023	189.4173	159.8382
	miR-181c-5p	8962.72577	4700.026745	11799.42224	9939.867445	12745.434	9471.251423	12145.90477	10463.78666	11374.23543	8487.392	10718.85	11327.98
	miR-181d-5p	506.1643172	272.6474759	622.5912002	581.7143012	707.6880298	503.0411712	556.6977131	763.0750414	582.3548844	467.1343	597.4812	634.0425
miR-182	*miR-182-5p*	12436.53599	10055.20507	16734.37134	6450.227807	7036.541689	6819.791796	3645.020754	3790.198766	4164.837975	13075.37	6768.854	3866.686
miR-183	*miR-183-3p*	14.94701548	10.75603961	22.59451249	13.89264924	19.97348618	11.62354439	10.49430159	7.324055586	6.783399935	16.09919	15.16323	8.200586
	miR-183-5p	1416.870935	1992.284415	2268.205144	1294.277973	1116.948678	955.0678976	730.6032824	727.3702704	596.2608543	1892.453	1122.098	684.7448
miR-184	*miR-184-3p*	3.979974537	4.471611971	2.957396923	58.47836074	65.79501329	29.70461345	66.4639101	184.4746501	34.76492467	3.802994	51.326	95.23449
miR-185	*miR-185-5p*	13.08969403	5.438446992	7.925823753	26.16987415	37.59715045	13.56080179	10.99403024	16.02137159	4.917964953	8.817988	25.77594	10.64446
miR-186	*miR-186-5p*	572.9394456	424.4405742	762.8901102	1015.617276	1328.041012	1290.213428	560.1958137	796.9487984	968.1607557	586.7567	1211.291	775.1018
miR-187	*miR-187-3p*	106.0442104	65.86563579	132.4913821	156.3730751	197.3850399	175.6446708	50.47259338	89.71968093	60.88101442	101.4671	176.4676	67.02443
miR-190	*miR-190a-5p*	20.07676044	2.53794193	13.13084234	189.6508163	95.95106104	118.8184538	93.9489857	128.6287262	45.78794956	11.91518	134.8068	89.45522
miR-191	*miR-191-5p*	11004.54115	23291.05565	22333.0786	7493.145755	13121.01387	8689.245186	4814.385789	5629.909978	8123.799762	18876.23	9767.802	6189.365
miR-192	*miR-192-5p*	1155.43083	1009.254907	1213.124218	416.7794771	443.7247027	426.1966278	747.5940564	717.2996939	686.4800734	1125.937	428.9003	717.1246
miR-193	*miR-193a-3p*	4.510637809	1.329398154	13.36743409	12.60030977	4.699643806	10.33203946	21.48833184	30.21172929	26.28567475	6.40249	9.210664	25.99525
	miR-193b-3p	4.156862294	2.779650685	10.29174129	8.238664083	11.35747253	17.43531659	36.48019126	32.04274319	33.57782968	5.742751	12.34382	34.03359
miR-194	*miR-194-5p*	53.3316588	30.81786629	64.35295704	34.57008066	27.80622585	34.87063318	44.97557826	37.53578488	69.52984933	49.50083	32.41565	50.6804
miR-195	*miR-195a-5p*	233.3149517	207.386112	362.5768627	469.6038527	263.1800531	565.6791605	507.7243057	794.2022776	603.2138392	267.7593	432.821	635.0468
miR-196	*miR-196a-5p*	7.252398045	2.417087552	6.387977353	97.8947144	58.35391059	91.05109775	433.2647372	397.3300155	154.8311035	5.352488	82.43324	328.4753
	miR-196b-5p	7.60617356	2.900505063	10.7649248	100.4793933	83.02704058	98.15437488	1280.304794	1194.278814	663.0773436	7.090534	93.88694	1045.887
miR-199	*miR-199a-3p*	1338.332771	667.4787275	2120.098706	2982.719483	2382.327773	3713.722434	3676.503659	5021.097858	5624.286471	1375.303	3026.257	4773.963
	miR-199a-5p	303.4509475	203.3979175	502.7574769	665.7163664	618.3947975	626.3798923	392.7867168	571.7340892	645.6100888	336.5354	636.8304	536.7103
	miR-199b-3p	1321.793766	660.3483192	2098.213969	2960.749712	2365.487382	3693.058355	3659.512885	4989.512868	5579.855202	1360.119	3006.432	4742.96
	miR-199b-5p	45.19482196	20.30353544	95.93795618	106.6180058	69.31974614	138.8367803	157.4145239	200.0382682	197.5665231	53.8121	104.9248	185.0064
miR-200	*miR-200a-3p*	2527.72605	1146.787189	2786.932564	4565.027614	4429.805924	4888.991922	3308.703374	3153.463683	4033.748771	2153.815	4627.942	3498.639
	miR-200a-5p	37.94242392	28.40077874	63.64318178	39.90098095	50.52117092	58.76347443	55.9696085	39.36679877	69.52984933	43.32879	49.72854	54.95542
	miR-200b-3p	4675.939418	1861.761687	4465.19617	2661.088499	2238.597	1632.462235	2734.515158	2669.160508	1870.692117	3667.632	2177.383	2424.789
	miR-200b-5p	22.99540844	21.27037046	30.52033624	15.99270087	17.23202729	14.85230672	31.98263343	19.22564591	23.23314478	24.92871	16.02568	24.81381
	miR-200c-3p	1212.034912	866.0424699	1711.268155	1814.121523	2252.304294	1439.382247	1254.818633	1482.663503	1586.298075	1263.115	1835.269	1441.26
miR-203	*miR-203-3p*	358.1977083	239.0499589	582.8437856	350.5470796	424.9261275	429.4253901	195.3939011	165.2490042	652.2239038	393.3638	401.6329	337.6223
miR-204	*miR-204-5p*	8465.494284	12950.39254	13049.45477	4027.252855	7369.433125	5934.465165	208.8865746	259.0884664	631.025779	11488.45	5777.05	366.3336
miR-205	*miR-205-5p*	1828.754078	874.5022764	1784.375007	1484.898044	1897.872824	1111.985747	1451.211992	1579.249486	1085.852745	1495.877	1498.252	1372.105
miR-208	*miR-208b-3p*	0	0.241708755	0.118295877	0.161542433	0.391636984	0.645752466	29.48399019	36.62027793	27.64235474	0.120002	0.399644	31.24887
miR-210	*miR-210-3p*	453.2748778	212.0994327	642.2283158	782.8346303	1101.283199	684.4976143	363.8024553	348.3503938	300.6742021	435.8675	856.2051	337.609
	miR-210-5p	15.12390324	8.459806432	22.23962486	10.98488544	23.49821903	19.37257399	2.998371884	5.950795164	12.37970488	15.27444	17.95189	7.109624
miR-211	*miR-211-5p*	30.86691363	13.41483591	28.62760221	20.51588899	15.66547935	10.97779193	3.498100531	1.373260422	1.356679987	24.30312	15.71972	2.076014
miR-214	*miR-214-3p*	41.39173518	35.89375015	106.4662892	104.0333269	75.97757487	105.9034045	43.97612097	72.78280239	101.920584	61.25059	95.30477	72.89317
	miR-214-5p	18.57321451	7.130408279	26.97145994	40.38560825	34.07241759	21.95558385	13.49267348	20.59890634	33.06907468	17.55836	32.13787	22.38688
miR-218	*miR-218-5p*	43.42594439	13.53569029	65.77250757	127.6185221	87.72668438	116.8811964	79.45685493	84.22663924	67.49482935	40.91138	110.7421	77.05944
miR-221	*miR-221-3p*	326.8001314	183.698654	405.5182661	1077.488028	866.3010083	1213.368884	2544.618272	3089.378197	1914.445047	305.339	1052.386	2516.147
	miR-221-5p	18.30788287	9.184932698	15.85164751	17.6081252	7.832739677	4.520267264	17.49050266	21.97216676	12.21011988	14.44815	9.987044	17.22426
miR-222	*miR-222-3p*	150.2661497	98.37546337	201.2212866	265.252675	233.0240054	192.434235	972.4719477	585.0089399	542.8415798	149.9543	230.237	700.1075
miR-223	*miR-223-3p*	27.77137788	20.06182668	37.02660947	52.98591802	76.76084883	72.97002869	110.9397597	54.47266342	163.3103534	28.2866	67.57227	109.5743
miR-296	*miR-296-5p*	6.721734774	5.317592615	18.09926917	4.84627299	5.09128079	5.811772197	0.999457295	5.035288215	7.122569932	10.0462	5.249775	4.385772
miR-298	*miR-298-5p*	11.1439287	7.130408279	22.94940012	6.784782186	7.049465709	1.937257399	3.997829179	5.950795164	3.900454963	13.74125	5.257168	4.61636
miR-299	*miR-299a-3p*	4.245306173	2.054524419	10.7649248	5.653985155	8.224376661	10.33203946	9.4948443	19.22564591	16.95849984	5.688252	8.070134	15.22633
	miR-299a-5p	2.653316358	2.53794193	4.850130954	3.553933526	1.566547935	4.520267264	16.99077401	6.866302112	11.53177989	3.34713	3.213583	11.79629
miR-300	*miR-300-3p*	40.59574028	28.52163311	91.32441698	57.50910615	81.46049264	92.34260268	58.96798039	84.68439271	166.3628834	53.4806	77.10407	103.3384
miR-301	*miR-301a-3p*	144.6941854	45.3203916	205.2433464	173.1734882	158.2213415	187.9139677	172.4063833	186.7634174	164.6670334	131.7526	173.1029	174.6123
miR-320	*miR-320-3p*	97.99581749	67.67845146	167.5069617	130.3647434	206.3926905	211.1610565	116.4367748	94.75496914	108.703984	111.0604	182.6395	106.6319
miR-322	*miR-322-3p*	19.72298493	11.36031149	33.59602904	26.81604388	21.14839713	58.76347443	4.497557826	14.64811117	19.33268981	21.55978	35.57597	12.82612
	miR-322-5p	163.2673999	53.65934366	188.9185154	90.30222005	63.83682837	97.50862241	60.96689498	62.71222595	78.34826925	135.2818	83.88256	67.34246
miR-324	*miR-324-3p*	5.837295988	4.834175104	15.26016812	19.22354953	27.80622585	11.62354439	7.995658358	5.493041689	7.461739929	8.64388	19.55111	6.98348
	miR-324-5p	13.53191343	10.87689398	27.79953108	24.71599225	27.80622585	15.49805919	11.99348754	24.7186876	19.67185981	17.40278	22.67343	18.79468
miR-326	*miR-326-3p*	5.483520473	3.988194461	16.32483101	11.14642788	9.399287612	13.56080179	10.99403024	37.53578488	27.30318474	8.598849	11.36884	25.27767
miR-328	*miR-328-3p*	24.14517886	24.41258428	55.95394978	35.21625039	63.44519138	35.51638565	53.97069391	45.77534741	55.96304946	34.83724	44.72594	51.90303
miR-329	*miR-329-3p*	1.768877572	2.296233175	3.312284554	3.715475959	3.133095871	5.16601973	16.49104536	10.98608338	11.70136489	2.459132	4.004864	13.0595
	miR-329-5p	3.891530658	1.691961286	6.624569107	4.200103258	11.35747253	5.811772197	8.995115652	12.3593438	11.53177989	4.069354	7.123116	10.96208
miR-330	*miR-330-5p*	41.65706682	16.43619535	46.01709612	44.74725394	50.9128079	39.39090044	11.99348754	16.47912507	14.92347986	34.70345	45.01699	14.46536
miR-331	*miR-331-3p*	22.28785741	24.41258428	60.21260135	66.87856726	54.43754075	75.55303856	10.99403024	19.22564591	63.93354439	35.63768	65.62305	31.38441
miR-335	*miR-335-3p*	9.905714403	7.613825789	31.93988677	62.03229427	43.86334219	29.70461345	96.44762894	206.4468168	123.7970488	16.48648	45.20008	142.2305
	miR-335-5p	19.54609717	7.130408279	27.20805169	93.53306871	52.87099282	23.24708879	27.98480425	65.91650027	123.6274638	17.96152	56.55038	72.50959
miR-337	*miR-337-3p*	1.591989815	0.966835021	3.548876307	4.361645691	3.133095871	3.874514798	5.497015121	16.02137159	9.835929906	2.0359	3.789752	10.45144
	miR-337-5p	5.129744959	2.296233175	11.00151655	5.815527588	4.308006822	9.040534528	11.99348754	14.64811117	13.22762987	6.142498	6.388023	13.28974
miR-338	*miR-338-3p*	24.23362274	8.459806432	28.50930634	29.56226524	20.36512316	9.040534528	57.96852309	69.12077459	50.53632952	20.40091	19.65597	59.20854
	miR-338-5p	1.768877572	0.48341751	1.301254646	1.292339464	1.174910952	2.583009865	26.98534696	31.58498971	12.54928988	1.184517	1.68342	23.70654
miR-339	*miR-339-3p*	19.54609717	9.426641453	27.20805169	13.08493707	14.88220539	12.26929686	7.995658358	10.98608338	16.78891484	18.72693	13.41215	11.92355
	miR-339-5p	27.41760237	23.2040405	54.06121575	42.48565988	35.63896553	30.99611838	43.47639232	43.94433352	76.99158926	34.89429	36.37358	54.80411
miR-340	*miR-340-3p*	21.66875026	9.910058964	22.35792074	14.7003614	10.18256158	8.394782062	8.995115652	19.68339939	14.58430986	17.97891	11.09257	14.42094
	miR-340-5p	857.6402908	352.4113651	1641.473588	798.8273312	756.2510158	1009.956857	1040.934772	1056.495018	1109.255474	950.5084	855.0117	1068.895
miR-341	*miR-341-3p*	6.544847016	5.921864503	20.93837021	14.53881897	15.27384237	14.85230672	26.48561831	27.46520845	31.7123947	11.13503	14.88832	28.55441
miR-342	*miR-342-3p*	29.89403097	19.21584604	27.91782695	52.33974829	21.54003411	52.95170224	93.9489857	92.0084483	119.7270089	25.6759	42.27716	101.8948
miR-345	*miR-345-3p*	20.43053596	12.68970965	36.79001772	31.98540173	45.42989013	23.24708879	43.97612097	36.16252446	41.8874946	23.30342	33.55413	40.67538
	miR-345-5p	77.38839378	72.63348094	148.934509	230.0364246	235.7654643	143.3570475	195.8936298	248.5601364	234.7056378	99.65213	203.053	226.3865
miR-350	*miR-350-3p*	8.313724588	5.317592615	10.29174129	8.884833815	8.616013645	27.12160358	54.47042256	63.6277329	93.9500891	7.974353	14.87415	70.68275
miR-351	*miR-351-5p*	202.7133698	178.8644789	342.4665637	154.9191932	173.4951838	223.4303533	55.46987986	66.37425375	136.5159237	241.3481	183.9482	86.12002
miR-361	*miR-361-3p*	18.66165838	12.56885527	22.94940012	26.00833171	39.16369838	30.35036592	16.49104536	12.3593438	27.47276974	18.05997	31.8408	18.77439
	miR-361-5p	72.52398045	76.74252978	85.52791901	69.62478862	83.02704058	79.42755335	77.95766899	99.33250388	73.0911343	78.26481	77.35979	83.46044
miR-362	*miR-362-3p*	9.905714403	3.867340083	21.29325784	17.6081252	17.23202729	22.60133632	73.95983981	45.31759394	43.75292958	11.68877	19.14716	54.34345
miR-365	*miR-365-3p*	10.43637767	5.921864503	19.16393206	17.44658276	9.399287612	19.37257399	50.47259338	46.23310089	39.17413462	11.84072	15.40615	45.29328
miR-369	*miR-369-5p*	4.068418416	2.900505063	5.323314461	5.007815423	4.699643806	3.874514798	12.99294483	10.5283299	7.631324927	4.097413	4.527325	10.3842
miR-374	*miR-374b-5p*	20.60742371	26.10454556	32.05818264	47.65501773	40.33860934	97.50862241	93.9489857	213.3131189	234.7056378	26.25672	61.83408	180.6559
miR-375	*miR-375-3p*	1368.315246	1092.886137	1561.268984	507.404782	973.2179049	1089.384411	43.47639232	63.6277329	133.1242237	1340.823	856.669	80.07612
miR-376	*miR-376b-3p*	3.18397963	2.054524419	6.1513856	5.815527588	5.482917774	4.520267264	11.99348754	18.76789244	17.63683983	3.79663	5.272904	16.13274
miR-378	*miR-378a-3p*	376.2402596	241.7087552	747.5116462	406.279219	426.4926754	375.1821829	1120.391627	1174.595415	752.4486378	455.1536	402.6514	1015.812
	miR-378a-5p	3.007091872	3.504776951	7.097752615	4.84627299	6.266191742	2.583009865	10.49430159	25.17644108	15.77140485	4.53654	4.565158	17.14738
	miR-378c	98.17270525	86.65258874	210.8032527	97.73317196	109.6583555	99.44587981	261.8578112	305.7793207	176.8771533	131.8762	102.2791	248.1714
	miR-378d	10.25948992	5.196738237	13.95891348	13.24647951	10.18256158	10.33203946	92.44979976	76.90258365	36.79994465	9.805047	11.25369	68.71744
miR-379	*miR-379-5p*	4.687525566	2.53794193	10.7649248	10.33871571	9.399287612	10.97779193	31.48290478	26.09194802	21.0285398	5.996797	10.2386	26.20113
miR-381	*miR-381-3p*	24.41051049	21.0286617	74.05321895	44.10108421	54.43754075	94.92561254	52.97123662	85.14214619	110.9085889	39.8308	64.48808	83.00732
miR-409	*miR-409-3p*	11.49770422	9.910058964	22.12132898	15.34653113	15.66547935	20.01832645	36.48019126	36.62027793	36.46077465	14.5097	17.01011	36.52041
	miR-409-5p	6.279515381	3.142213818	6.033089723	10.50025814	4.699643806	6.457524663	16.99077401	37.53578488	6.783399935	5.151606	7.219142	20.43665
miR-410	*miR-410-3p*	26.26783194	33.83922573	94.16351802	84.80977732	77.5441228	156.9178493	139.9240213	164.7912507	205.028263	51.42353	106.4239	169.9145
miR-411	*miR-411-5p*	445.3149288	306.9701191	1025.506957	673.793488	637.1933727	743.9068412	819.5549816	1346.710721	1237.631318	592.5973	684.9646	1134.632
miR-421	*miR-421-3p*	59.5227303	28.88419625	50.27574769	72.04792512	99.08415691	60.70073183	35.98046261	74.61381628	96.15469408	46.22756	77.2776	68.91632
miR-423	*miR-423-3p*	124.8827566	61.87744133	185.9611185	80.7712165	129.2402047	65.2209991	60.46716633	70.03628154	87.33627416	124.2404	91.74414	72.61324
	miR-423-5p	73.49686312	45.80380911	96.17454793	56.70139398	84.20195153	54.24320717	37.9793772	42.11331962	38.49579463	71.82507	65.04885	39.5295
miR-425	*miR-425-3p*	12.64747464	12.20629214	13.01254646	15.02344627	10.96583555	12.26929686	8.495387005	11.90159033	8.30966492	12.6221	12.75286	9.568881
	miR-425-5p	370.2260758	222.6137635	419.1222919	418.7179863	531.0597501	387.4514798	290.3423441	313.1033763	470.4287855	337.3207	445.7431	357.9582
miR-429	*miR-429-3p*	3891.530658	1687.973092	3612.164602	4560.181341	4629.540786	3837.706907	4353.136247	3603.893102	3657.77883	3063.889	4342.476	3871.603
miR-431	*miR-431-5p*	4.687525566	3.142213818	13.36743409	6.46169732	5.874554758	6.457524663	18.48995995	10.98608338	21.3677098	7.065724	6.264592	16.94792
miR-434	*miR-434-3p*	175.9148745	111.6694449	436.0386023	252.4908228	190.3355741	406.8240538	726.1057246	1091.284282	1319.880042	241.2076	283.2168	1045.757
	miR-434-5p	57.22318945	51.48396486	124.8021501	89.00988058	121.799102	120.1099587	54.47042256	56.30367732	241.1498677	77.83643	110.3063	117.308
miR-449	*miR-449a-5p*	16.5390053	8.218097677	23.89576714	14.05419167	25.84804093	27.12160358	35.48073396	47.60636131	50.36674452	16.21762	22.34128	44.48461
miR-450	*miR-450a-5p*	23.87984722	8.701515188	29.33737748	25.03907711	16.84039031	46.49417757	14.49213077	31.12723624	10.5142699	20.63958	29.45788	18.71121
	miR-450b-5p	11.32081646	3.263068195	17.50778978	6.623239753	2.741458887	9.040534528	3.997829179	8.239562534	3.052529971	10.69722	6.135078	5.096641
miR-451	*miR-451a*	19.54609717	21.51207921	76.06424886	72.20946755	157.0464305	362.9128861	51.97177932	379.9353835	156.8661235	39.04081	197.3896	196.2578
miR-455	*miR-455-3p*	6.721734774	12.56885527	29.69226511	8.723291382	31.33095871	10.97779193	5.996743768	10.07057643	15.60181985	16.32762	17.01068	10.55638
	miR-455-5p	10.43637767	2.779650685	21.76644135	17.28504033	15.66547935	27.76735605	12.49321618	28.83846887	30.18612971	11.66082	20.23929	23.83927
miR-463	*miR-463-5p*	3.449311265	1.450252531	0.828071138	11.30797031	12.53238348	12.26929686	13.49267348	14.64811117	32.89948968	1.909212	12.03655	20.34676
miR-465	*miR-465a-3p*	7.517729681	6.163573258	6.861160861	19.70817683	35.24732855	18.08106906	31.98263343	27.00745497	54.43678448	6.847488	24.34552	37.80896
	miR-465a-5p	3.714642901	3.142213818	3.430580431	17.44658276	14.09893142	14.85230672	23.98697507	25.63419455	40.36122961	3.429146	15.46594	29.99413
	miR-465b-3p	7.517729681	6.163573258	6.861160861	19.70817683	35.24732855	18.08106906	31.98263343	27.00745497	54.43678448	6.847488	24.34552	37.80896
	miR-465b-5p	2.211096965	2.175378797	5.205018584	12.76185221	8.616013645	1.937257399	84.95387005	81.48011839	155.6790285	3.197165	7.771708	107.371
	miR-465c-3p	7.517729681	6.163573258	6.742864984	19.70817683	35.24732855	18.08106906	31.98263343	26.5497015	54.26719948	6.808056	24.34552	37.59984
	miR-465c-5p	28.9211483	14.50252531	18.09926917	115.0182123	121.015828	157.5636018	127.4308051	171.6575528	228.7701628	20.50765	131.1992	175.9528
miR-466	*miR-466a-3p*	12.73591852	5.921864503	19.04563618	4.684730557	3.133095871	7.103277129	2.998371884	2.746520845	4.239624959	12.56781	4.973701	3.328173

	miR-466b-3p	13.88568894	6.04271888	20.70177846	5.169357856	3.133095871	5.16601973	2.998371884	2.746520845	4.748379955	13.5434	4.489491	3.497758
	miR-466c-3p	13.88568894	6.04271888	20.70177846	5.169357856	3.133095871	7.103277129	2.998371884	2.746520845	4.748379955	13.5434	5.135244	3.497758
	miR-466e-3p	13.88568894	6.04271888	20.82007434	5.169357856	3.133095871	7.103277129	2.998371884	2.746520845	4.748379955	13.58283	5.135244	3.497758
	miR-466i-5p	0.795994907	6.767845146	20.22859495	11.79259761	17.62366427	37.45364304	24.98643237	21.05665981	29.67737472	9.264145	22.28997	25.24016
	miR-466p-3p	13.88568894	6.04271888	20.70177846	5.169357856	3.133095871	7.103277129	2.998371884	2.746520845	4.748379955	13.5434	5.135244	3.497758
miR-467	*miR-467a-3p*	9.021275617	8.338952055	26.85316406	9.046376248	4.699643806	4.520267264	2.498643237	5.035288215	14.41472486	14.7378	6.088762	7.316219
	miR-467a-5p	79.7763785	40.12365336	100.4331995	41.83949015	42.29679426	25.18434619	15.99131672	16.02137159	15.43223485	73.44441	36.44021	15.81497
	miR-467b-5p	79.7763785	40.12365336	100.4331995	41.83949015	42.29679426	25.18434619	15.99131672	16.02137159	15.43223485	73.44441	36.44021	15.81497
	miR-467c-5p	21.93408189	7.130408279	32.64966203	8.400206516	10.18256158	3.228762331	1.998914589	4.577534741	5.426719948	20.57138	7.27051	4.001056
	miR-467d-3p	9.021275617	8.338952055	26.85316406	9.046376248	4.699643806	4.520267264	2.498643237	5.035288215	14.41472486	14.7378	6.088762	7.316219
	miR-467d-5p	36.08510247	17.76559351	49.6842683	11.14642788	19.19021221	9.686286994	6.496472415	5.493041689	7.461739929	34.51165	13.34098	6.483751
	miR-467e-5p	25.73716867	10.15176772	43.29629095	14.2157341	12.53238348	12.91504933	9.994572947	7.324055586	8.648834917	26.39508	13.22106	8.655821
miR-470	*miR-470-3p*	0.530663272	0	0.236591754	1.776966763	1.174910952	1.291504933	23.48724643	3.204274319	7.800909925	0.255752	1.414461	11.49748
	miR-470-5p	51.38589347	74.56715098	33.24114141	169.9426395	277.2789846	170.4786511	174.9050266	153.8051673	253.0208176	53.06473	205.9001	193.9103
miR-471	*miR-471-5p*	0.61910715	0.120854378	0.354887631	2.584678928	1.566547935	3.874514798	16.99077401	10.07057643	26.96401474	0.36495	2.675247	18.00846
miR-484	*miR-484*	48.73257711	39.88194461	74.76299421	57.83219101	64.22846535	43.91116771	32.48236208	23.80318065	47.14462955	54.45917	55.32394	34.47672
miR-486	*miR-486a-3p*	0.088443879	0.120854378	0.118295877	0.161542433	1.174910952	1.937257399	16.99077401	13.27485075	10.5142699	0.109198	1.091237	13.5933
	miR-486a-5p	101.268241	209.9240539	380.4395402	155.8884478	470.7476546	280.9023228	2145.335083	1672.631194	1428.075271	230.5439	302.5128	1748.681
	miR-486b-5p	101.0913532	209.4406364	379.611469	155.565363	471.1392916	280.9023228	2139.838068	1666.222646	1424.005231	230.0478	302.5357	1743.355
miR-496	*miR-496a-3p*	2.741760237	1.691961286	2.839101046	3.392391093	1.958184919	4.520267264	23.48724643	15.56361812	12.04053488	2.424274	3.290281	17.03047
miR-497	*miR-497a-5p*	223.7630129	46.64978976	297.2775387	203.3819231	106.5252596	118.8184538	198.8920016	252.6799177	162.4624284	189.2301	142.9085	204.6781
miR-500	*miR-500-3p*	7.340841924	10.03091334	19.16393206	14.05419167	17.23202729	12.91504933	25.48616101	33.87375708	36.46077465	12.17856	14.73376	31.94023
miR-501	*miR-501-3p*	58.37295988	41.33219714	36.43513009	70.10941592	40.73024632	54.88895963	116.4367748	125.4244519	75.46532428	45.3801	55.24287	105.7755
miR-511	*miR-511-3p*	4.333750051	1.933670042	9.227078399	17.44658276	12.1407465	20.66407892	9.4948443	17.39463202	26.96401474	5.164833	16.75047	17.95116
miR-532	*miR-532-3p*	8.755943981	5.801010125	13.01254646	8.238664083	8.616013645	10.97779193	5.996743768	9.155069482	22.72438978	9.189834	9.27749	12.6254
	miR-532-5p	73.93908251	47.85833353	110.488349	108.7180574	147.2555059	154.9805919	164.410725	225.6724627	246.9157576	77.42859	136.9847	212.333
miR-541	*miR-541-5p*	75.53107232	80.12645235	155.9139658	77.37882541	103.0005268	77.49029595	167.4090969	206.4468168	162.9711834	103.8572	85.95655	178.9424
miR-542	*miR-542-3p*	9.728826646	4.109048839	14.55039286	8.723291382	7.441102693	18.08106906	6.496472415	7.78180906	5.426719948	9.462756	11.41515	6.568334
miR-574	*miR-574-3p*	28.74426055	32.99324509	53.35144049	73.98643431	66.57828725	78.13604842	62.96580957	73.24055586	129.3933538	38.36298	72.90026	88.53324
	miR-574-5p	18.75010226	13.41483591	26.02509292	42.00103258	42.29679426	29.05886098	38.97883449	52.18389605	27.30318474	19.39668	37.78556	39.48864
miR-582	*miR-582-3p*	40.33040864	33.11409946	96.88432319	14.7003614	27.02295189	22.60133632	15.99131672	11.44383685	33.91699968	56.77628	21.44155	20.45072
	miR-582-5p	18.21943899	8.58066081	32.53136615	11.79259761	5.09128079	5.811772197	18.9896886	14.1903577	13.39721487	19.77716	7.565217	15.52575
miR-598	*miR-598-3p*	9.905714403	7.492971412	11.11981243	26.16987415	25.84804093	21.30983139	6.496472415	6.866302112	9.157589912	9.506166	24.44258	7.506788
miR-615	*miR-615-3p*	12.91280628	13.05227278	21.17496197	30.04689254	41.51352029	26.47585112	17.49050266	18.76789244	34.25616967	15.71335	32.67875	23.50485
miR-652	*miR-652-3p*	183.3441603	143.0915831	255.5190941	228.2594578	313.3095871	235.6996502	246.8659518	249.9333969	218.4254779	193.9849	259.0896	238.4083
miR-664	*miR-664-3p*	7.60617356	13.41483591	15.85164751	14.05419167	17.23202729	29.05886098	12.49321618	26.09194802	39.85247462	12.29089	20.11503	26.14588
miR-669	*miR-669a-3p*	32.28201569	17.16132162	70.26775089	24.39290738	15.66547935	14.85230672	2.498643237	6.408548638	19.50227481	39.9037	18.30356	9.469822
	miR-669a-5p	25.2065054	13.41483591	25.55190941	12.27722491	14.88220539	7.103277129	14.49213077	12.81709728	10.6838549	21.39108	11.4209	12.66436
	miR-669c-5p	17.95410736	10.63518523	25.19702178	5.977070021	7.441102693	4.520267264	4.997286473	2.746520845	3.561284966	17.92877	5.97948	3.768364
	miR-669o-3p	32.28201569	17.16132162	70.26775089	24.39290738	15.66547935	14.85230672	2.498643237	6.408548638	19.50227481	39.9037	18.30356	9.469822
	miR-669p-5p	25.2065054	13.41483591	25.55190941	12.27722491	14.88220539	7.103277129	14.49213077	12.81709728	10.6838549	21.39108	11.4209	12.66436
miR-671	*miR-671-3p*	19.28076553	13.17312716	29.33737748	3.715475959	6.657828725	3.874514798	2.998371884	3.662027793	4.070039961	20.59709	4.749273	3.576813
miR-672	*miR-672-5p*	224.2052323	129.1933297	216.9546383	171.5580638	251.4309436	158.8551067	79.45685493	93.38170872	121.9316138	190.1177	193.948	98.25673
miR-674	*miR-674-3p*	14.0625767	5.921864503	20.22859495	6.300154887	7.049465709	7.749029595	4.997286473	12.3593438	14.41472486	13.40435	7.032883	10.59045
miR-676	*miR-676-3p*	55.98497515	38.3108377	80.4411963	82.54818326	81.85212962	141.4197901	145.920765	148.7698791	176.7075683	58.24567	101.94	157.1327
	miR-676-5p	9.286607253	3.383922573	8.635599015	10.17717328	15.66547935	20.01832645	23.98697507	22.88767371	27.30318474	7.102043	15.28699	24.72594
miR-677	*miR-677-3p*	3.095535751	5.196738237	5.796497969	8.561748949	27.80622585	29.70461345	0.999457295	6.866302112	9.327174911	4.696257	22.0242	5.730978
	miR-677-5p	41.48017906	25.74198243	73.22514781	87.07137139	146.8638689	294.4631246	62.46608092	78.27584407	91.06714413	46.81577	176.1328	77.26969
miR-690	*miR-690*	272.4071461	382.6249595	668.3717046	779.1191543	1339.006848	891.1384035	391.2875309	449.9716651	735.9988929	441.1346	1003.088	525.7527
miR-708	*miR-708-3p*	3.80308678	1.450252531	6.387977353	20.83897386	11.74910952	10.97779193	20.98860319	27.00745497	24.58982476	3.880439	14.52196	24.19529
	miR-708-5p	5.925739866	1.571106909	7.334344369	26.81604388	25.84804093	14.20655426	35.98046261	58.59244469	23.23314478	4.94373	22.29021	39.26868
miR-709	*miR-709*	1.326658179	4.471611971	12.3027712	15.99270087	47.38807505	15.49805919	18.48995995	19.22564591	14.41472486	6.03368	26.29295	17.37678
miR-714	*miR-714*	1.061326543	13.17312716	33.95091667	4.361645691	30.15604776	3.228762331	7.995658358	5.493041689	3.900454963	16.06179	12.58215	5.796385
miR-741	*miR-741-3p*	30.07091872	13.17312716	7.925823753	106.2949209	50.12953393	32.93337578	68.96255333	71.40954196	56.47180446	17.05662	63.11928	65.61463
miR-743	*miR-743a-3p*	2.564872479	1.329398154	1.774438154	12.11568247	8.224376661	14.85230672	4.497557826	19.22564591	22.38521979	1.88957	11.73079	15.36947
	miR-743b-3p	25.47183704	13.05227278	17.15290215	98.05625683	84.59358851	92.34260268	46.4747642	67.74751417	173.3158683	18.559	91.66415	95.84605
miR-744	*miR-744-5p*	283.2857432	155.6604384	462.5368787	141.3496289	216.9668891	92.98835515	80.95604087	130.9174936	91.91506912	300.4944	150.435	101.2629
miR-871	*miR-871-3p*	72.25864882	63.20683949	38.80104763	224.7055243	182.1111975	131.7335031	172.906112	154.7206743	148.5564586	58.08885	179.5167	158.7277
	miR-871-5p	7.163954167	3.504776951	4.850130954	17.76966763	21.14839713	21.95558385	27.98480425	31.12723624	65.79897937	5.172954	20.29122	41.63701
miR-872	*miR-872-3p*	60.84938848	24.65429303	80.91437981	56.37830912	37.98878743	45.84842511	66.4639101	77.36033713	77.66992926	55.47269	46.73851	73.83139
	miR-872-5p	298.2327586	100.4299878	269.8328952	332.1312422	309.0015803	241.5114224	261.8578112	435.3235539	229.7876728	222.8319	294.2147	308.9897
miR-874	*miR-874-3p*	15.300791	18.12815664	21.41155372	16.47732817	19.19021221	12.91504933	17.49050266	19.22564591	15.09306486	18.28017	16.1942	17.26974
miR-878	*miR-878-5p*	4.864413323	2.779650685	3.785468061	22.77748305	24.281493	34.22488071	25.48616101	22.88767371	63.42478939	3.809844	27.09462	37.26621
miR-879	*miR-879-5p*	9.994158282	2.175378797	30.756928	1.776966763	0.391636984	2.583009865	0.999457295	4.577534741	0.847924992	14.30882	1.583871	2.141639
miR-880	*miR-880-3p*	1.415102058	0.845980643	1.301254646	9.046376248	9.399287612	10.97779193	7.995658358	5.035288215	23.06355978	1.187446	9.807819	12.0315
miR-881	*miR-881-3p*	34.13933714	24.05002114	27.20805169	148.1344111	195.8184919	219.5558385	242.8681226	271.9055636	409.0390161	28.4658	187.8362	307.9376
miR-883	*miR-883a-3p*	4.599081687	1.208543776	3.430580431	12.92339464	15.27384237	10.97779193	9.4948443	9.155069482	24.25065477	3.079402	13.05834	14.30019
miR-1195	*miR-1195*	5.395076595	15.46936033	33.8326208	83.19435299	135.5063964	36.16213811	61.96635227	63.6277329	42.2266646	18.23235	84.9543	55.94025
miR-1198	*miR-1198-5p*	22.46474516	16.43619535	29.57396923	27.13912874	39.16369838	36.80789058	19.48941725	38.9090453	29.84695971	22.82497	34.37024	29.41514
miR-1247	*miR-1247-5p*	27.85982176	30.33444878	61.15896837	32.14694417	61.09536948	31.64187085	17.9902313	26.09194802	76.31324927	39.78441	41.62806	40.13181
miR-1249	*miR-1249-3p*	7.429285802	12.81056403	24.25065477	8.884833815	10.18256158	16.14381166	9.994572947	17.39463202	23.06355978	14.83017	11.73707	16.81759
miR-1251	*miR-1251-5p*	19.81142881	7.855534544	32.88625378	20.51588899	17.62366427	29.05886098	5.996743768	15.56361812	20.35019981	20.18441	22.39947	13.97019
miR-1291	*miR-1291*	2.299540844	1.087689398	5.559906215	4.84627299	3.133095871	2.583009865	18.9896886	14.1903577	2.543774976	2.982379	3.520793	11.90794
miR-1839	*miR-1839-3p*	28.21359727	21.39122484	32.64966203	76.73265567	83.41867756	51.6601973	19.98914589	20.14115286	18.82393482	27.41816	70.60384	19.65141
	miR-1839-5p	336.7058458	150.4637001	329.2174255	442.9493513	547.1168664	413.2815784	480.7389587	434.8658004	304.7442421	272.129	467.7826	406.783
miR-1843	*miR-1843a-5p*	78.98038359	46.16637225	125.9851089	79.64041947	89.29323232	61.99223676	61.46662362	50.35288215	53.41927449	83.71062	76.9753	55.07959
	miR-1843b-3p	8.313724588	6.646990768	16.67971865	5.653985155	12.53238348	7.103277129	2.498643237	8.239562534	10.0055149	10.54681	8.429882	6.914574
	miR-1843b-5p	91.62785823	70.21639339	155.4407823	119.0567731	118.2743691	108.4864143	96.44762894	83.31113229	92.08465412	105.7617	115.2725	90.61447
miR-1957	*miR-1957a*	3.272423508	4.350757594	30.756928	18.41583736	16.44875332	3.874514798	71.96092522	100.7057643	453.8094557	12.79337	12.91304	208.8254
miR-1981	*miR-1981-3p*	9.994158282	7.492971412	8.635599015	16.1542433	13.31565745	5.811772197	5.996743768	6.408548638	10.3446849	8.707576	11.76056	7.583326
	miR-1981-5p	5.748852109	5.196738237	8.990486646	14.37727654	22.32330808	18.72682152	17.49050266	17.39463202	22.04604979	6.645359	18.4758	18.97706
miR-3068	*miR-3068-3p*	95.16561337	47.25406164	166.2057071	70.27095835	87.3350474	27.76735605	22.48778913	25.63419455	27.30318474	102.8751	61.79112	25.14172
miR-3470	*miR-3470a*	84.90612346	117.7121638	407.8841836	800.7658404	739.0189885	741.9695838	263.3569972	335.5332965	357.4851766	203.5008	760.5848	318.7918
	miR-3470b	99.23403179	194.9381111	481.9374026	992.032081	1155.720739	1031.912441	280.8474998	302.5750464	353.4151366	258.7032	1059.888	312.2792
miR-3473	*miR-3473a*	14.68168385	13.65654467	41.28526104	111.3027363	159.3962524	72.97002869	74.45956845	69.12077459	61.38976941	23.20783	114.5563	68.32337
	miR-3473b	14.8585716	35.28947826	76.65572824	187.8738496	358.3478402	111.7151767	194.8941725	156.5516881	132.6154687	42.26793	219.3123	161.3538
	miR-3473e	13.08969403	30.2135944	64.23466117	167.8425879	341.8990869	84.59357308	182.9006849	146.0233582	121.7620288	35.84598	198.1117	150.2287
	miR-3473f	0.795994907	4.592466349	8.517303138	37.47784446	61.09536948	2.583009865	30.48344749	24.26093413	13.39721487	4.635255	33.71874	22.71387
miR-3535	*miR-3535*	22.37630129	32.50982758	32.05818264	128.1031494	286.2866352	633.4831694	59.96743768	88.34642051	52.2321795	28.98144	349.291	66.84868
miR-5099	*miR-5099*	96.05005216	66.59076206	266.6389066	116.7951791	113.9663623	64.57524663	210.3857605	181.2703758	92.93257911	143.0932	98.4456	161.5296
miR-5100	*miR-5100*	1.149770422	4.955029482	29.69226511	7.754036784	8.224376661	9.040534528	16.49104536	10.07057643	95.47635409	11.93236	8.339649	40.67933
miR-5119	*miR-5119*	0.176887757	0.845980643	4.850130954	0.969254598	1.174910952	1.937257399	1.998914589	2.288767371	73.93905929	1.957666	1.360474	26.07558
miR-5121	*miR-5121*	26.71005134	17.88644789	66.71887458	161.8655179	159.3962524	256.3637291	610.1686784	490.2539708	233.1793728	37.10512	192.5418	444.534
miR-5126	*miR-5126*	1.591989815	9.668350208	26.1433888	4.523188124	19.97348618	7.749029595	6.496472415	4.119781267	7.631324927	12.46791	10.74857	6.082526
miR-5128	*miR-5128*	1.680433693	3.625631328	8.517303138	9.854088413	29.76441077	16.14381166	15.49158807	13.73260422	12.04053488	4.607789	18.58744	13.75491
miR-5621	*miR-5621-5p*	2.122653086	0.604271888	7.452640246	29.23918037	51.69608187	0	13.49267348	8.697316008	2.204604979	3.393188	26.97842	8.131531
miR-6238	*miR-6238*	5.129744959	5.317592615	8.872190769	77.54036784	37.20551347	91.05109775	146.4204937	162.0447298	18.48476482	6.439843	68.59899	108.9833
miR-6240	*miR-6240*	481.7538067	8577.276887	3303.648955	1795.705685	6403.264686	1509.769266	2446.171729	1398.894617	741.5951979	4120.893	3236.247	1528.887
miR-6412	*miR-6412*	0.176887757	0.241708755	1.892734031	7.107867052	4.699643806	0.645752466	5.996743768	2.746520845	35.27367966	0.770444	4.151088	14.67231
miR-6538	*miR-6538*	1.768877572	21.39122484	55.3624704	6.946324619	46.60480108	35.51638565	5.996743768	12.81709728	16.11057485	26.17419	29.68917	11.64147
miR-6937	*miR-6937-5p*	4.42219393	5.680155747	41.75844455	10.50025814	24.67312998	0	8.995115652	12.81709728	62.5768644	17.28693	11.72446	28.12969
miR-7033	*miR-7033-5p*	7.783061317	17.52388475	48.61960541	121.9645369	718.6538654	328.6880053	28.4845329	29.29622234	127.6975038	24.64218	389.7688	61.82609
miR-7087	*miR-7087-3p*	0	0.120854378	0	0	0.783273968	0	1.998914589	0.915506948	34.25616967	0.040285	0.261091	12.3902

[1] miRNAs with normalized reads of < 10 in any one biological replicate are highlighted with grey shading, those with average normalized reads <10 in any one epididymal segment are highlighted with red shading

[2] All listed miRNAs had normalized reads of ≥ 10 in at least one epididymal segment

Table S2: Fold changes of epididymosome miRNA abundance between epididymal segments

miRNA Family (mmu)	Mature miRNA (mmu)	Average Normalised Reads			Log_2 Fold Change			False Discovery Rate (FDR)		
		Caput	Corpus	Cauda	Caput to Corpus	Corpus to Cauda	Caput to Cauda	Caput to Corpus	Corpus to Cauda	Caput to Cauda
let-7	*let-7a-1-3p*	47.4805	23.46847	50.31396	-1.508375205	0.950646318	-0.661765947	0.014166523	0.12314076	0.150975918
	let-7a-5p	4139.229	4962.573	4629.333	-0.164378084	-0.296368502	-0.575651985	0.591437534	0.312099824	0.021438366
	let-7b-3p	48.04911	16.95654	15.92811	-1.878439278	-0.335521948	-2.333828388	0.008661879	0.604548877	0.000580677
	let-7b-5p	1906.419	1566.208	1110.252	-0.751910365	-0.687409286	-1.554599352	0.044272876	0.060276259	0.000197038
	let-7c-2-3p	47.4805	23.46847	50.25744	-1.508375205	0.949058379	-0.663353885	0.014166523	0.12314076	0.150532668
	let-7c-5p	11498.27	9239.25	9133.079	-0.773823446	-0.202224807	-1.09140857	0.021481039	0.458474602	0.000285426
	let-7d-3p	113.4757	114.8893	116.1866	-0.454786755	-0.164983682	-0.737693317	0.388881341	0.785403765	0.09965146
	let-7d-5p	496.4964	752.7344	842.9378	0.155047971	-0.058924812	-0.018527843	0.706317176	0.891188938	0.952907649
	let-7e-5p	667.3349	545.0861	421.3005	-0.715976174	-0.595701943	-1.43131895	0.045105277	0.152404728	0.000771869
	let-7f-2-3p	17.36592	10.63037	9.03151	-0.982607849	-0.463300008	-1.558057301	0.061786123	0.452293973	0.00449756
	let-7f-5p	8883.147	11414.54	10081.79	-0.022042008	-0.395119073	-0.539990442	0.973345036	0.304732052	0.080541491
	let-7g-5p	1761.826	2496.859	2128.955	0.061037037	-0.475985148	-0.52773182	0.900566621	0.179014194	0.069754652
	let-7i-3p	12.79419	39.60262	27.22138	1.162441039	-0.752226128	0.288657606	0.045306939	0.14584525	0.487700092
	let-7i-5p	1385.335	2954.182	3698.336	0.65270461	0.101693227	0.635433547	0.06171389	0.789242365	0.037893554
	let-7j	281.6331	422.2923	521.0224	0.156894088	0.113451431	0.150640612	0.731113122	0.789122832	0.632938028
miR-1	*miR-1a-3p*	3.474834	3.689925	15.28195	-0.335194744	1.745703568	1.326282841	0.653830481	0.032359771	0.029027658
miR-7	*miR-7a-1-3p*	5.526085	10.83823	9.39475	0.424774406	-0.308153894	-0.001310781	0.459654774	0.603706959	0.997294102
miR-9	*miR-9-5p*	249.3823	38.42579	31.86304	-3.133899589	-0.617636358	-3.868572057	0.000289521	0.366291628	1.61282E-05
miR-10	*miR-10a-3p*	73.70297	62.508	69.90896	-0.678839673	-0.023058615	-0.820574789	0.094084267	0.951136451	0.027413165
	miR-10a-5p	245070.3	194731.9	148528.1	-0.842854375	-0.59884048	-1.545652387	0.145291328	0.220360126	0.00176624
	miR-10b-3p	60.39329	56.36332	43.96898	-0.591534129	-0.565610378	-1.28577844	0.317076002	0.366302246	0.016215297
	miR-10b-5p	371442.9	321396.2	188243.8	-0.712566139	-0.966535165	-1.790043486	0.244549848	0.056458594	0.000881205
miR-15	*miR-15a-5p*	83.20522	106.2308	163.2985	0.0711655	0.413311064	0.374918225	0.926092522	0.503062908	0.437776501
	miR-15b-3p	6.383291	9.95293	13.09022	0.095980933	0.249690955	0.221020629	0.915179221	0.697472209	0.647836386
	miR-15b-5p	56.8596	45.65567	61.52917	-0.764451138	0.161899762	-0.715664025	0.113378831	0.81043738	0.142447164
miR-16	*miR-16-5p*	1352.726	1820.211	3221.846	-0.007556332	0.621980276	0.505309685	0.987711499	0.056458594	0.055562791
miR-17	*miR-17-5p*	88.56668	106.9613	46.62079	-0.09531665	-1.417261206	-1.628186301	0.888003869	0.013093356	0.001691302
miR-18	*miR-18a-5p*	12.13374	62.85187	144.3698	1.700798329	1.117296408	2.705171208	0.036541734	0.14842597	0.001140525
miR-19	*miR-19a-3p*	18.47318	22.05437	112.8188	-0.057443237	2.008758377	1.841051632	0.945294712	0.00894678	0.0038263
	miR-19b-3p	211.5512	268.1963	698.9313	-0.059607915	1.108454267	0.93292704	0.926092522	0.053276006	0.043010246
miR-20	*miR-20a-5p*	61.55565	75.88934	48.84869	-0.01461527	-0.834744243	-0.960153839	0.985189775	0.127957863	0.037656139
miR-21	*miR-21a-3p*	56.3571	69.80334	57.86797	-0.082138923	-0.454212622	-0.654260027	0.900566621	0.358797791	0.093330702
miR-21	*miR-21a-5p*	12595.86	13447.57	20383.62	-0.357410709	0.412241526	-0.065865728	0.290474972	0.172867286	0.777542663
miR-22	*miR-22-3p*	18765.31	16161.97	22068.16	-0.622279416	0.227446877	-0.504587406	0.07510271	0.524496478	0.079176334
	miR-22-5p	22.47535	24.51141	46.13519	-0.145043843	0.627074905	0.363476097	0.819017069	0.282404763	0.418736566
miR-23	*miR-23a-3p*	335.9256	372.9157	774.8535	-0.30255342	0.831863083	0.414097679	0.430965685	0.030948687	0.143674503
	miR-23b-3p	562.8797	449.0512	988.0447	-0.781896354	0.91592786	0.023077738	0.077464655	0.042105121	0.948018748
miR-24	*miR-24-2-5p*	88.39438	98.14637	80.69239	-0.292009175	-0.618148774	-1.029109883	0.516540828	0.332923093	0.046627711
	miR-24-3p	348.3557	559.8674	630.6345	0.239664703	-0.085173581	0.04059099	0.535141838	0.839102283	0.899195909
miR-25	*miR-25-3p*	365.5301	722.513	1185.763	0.509011847	0.500700124	0.895823279	0.181505673	0.172867286	0.007537938
miR-26	*miR-26a-5p*	16351.81	19625.22	25643.85	-0.207953882	0.191873072	-0.125581835	0.512432721	0.470553774	0.541780763
	miR-26b-5p	1641.763	2479.106	2300.919	0.187417959	-0.305610854	-0.235149249	0.557054292	0.332923093	0.328949826
miR-27	*miR-27a-3p*	667.9182	767.9612	1152.257	-0.227065684	0.342478386	0.002552665	0.534362004	0.385570223	0.993218613
	miR-27b-3p	11584.4	12891.04	12985.36	-0.3520761	-0.218094751	-0.67123299	0.337542645	0.556358485	0.032352614
	miR-27b-5p	9.642597	12.84295	21.87287	-0.030269773	0.515678103	0.366678003	0.973345036	0.358797791	0.383603916
miR-28	*miR-28a-3p*	16.9381	23.88104	58.53734	0.002956412	1.137926711	1.02766925	0.997149345	0.034292081	0.020823409
	miR-28a-5p	57.61372	63.80862	92.32823	-0.254165336	0.309465544	-0.059910008	0.554492665	0.507309903	0.875877556
miR-29	*miR-29a-3p*	2049.666	3551.092	3715.223	0.369557891	-0.140706808	0.111834682	0.220441875	0.653268487	0.648779616
	miR-29a-5p	7.52252	7.433223	14.23918	-0.099043099	0.690240936	0.490115385	0.926092522	0.396797428	0.415580919
	miR-29b-3p	107.8041	148.5112	164.5063	0.171717456	-0.07444376	-0.018086938	0.756746674	0.895155059	0.968699746
	miR-29c-3p	261.9548	270.8644	298.0356	-0.320270169	-0.062051379	-0.502950265	0.430965685	0.895715681	0.152057678
miR-30	*miR-30a-3p*	163.3863	275.2666	601.0163	0.260102687	0.955632793	1.094999167	0.591437534	0.028146073	0.004463692
	miR-30a-5p	16068.65	34445.44	29557.26	0.621368551	-0.423546287	0.09459827	0.061632688	0.12314076	0.661107655
	miR-30b-5p	1023.367	1127.524	1327.407	-0.24566339	0.029620398	-0.330794555	0.516540828	0.946786958	0.322979252
	miR-30c-2-3p	19.94729	14.0822	21.50901	-0.942087826	0.383251671	-0.673403571	0.054671733	0.452855601	0.085764084
	miR-30c-5p	1953.964	1869	2816.565	-0.493162305	0.388352811	-0.214341059	0.160667415	0.310118848	0.456714271
	miR-30d-3p	30.73222	48.75805	43.82655	0.244402722	-0.332729376	-0.202683778	0.606786873	0.479976343	0.589235172
	miR-30d-5p	6981.096	9672.583	5954.318	-0.019091743	-0.911245343	-1.033667022	0.973345036	0.012964842	0.001797627
	miR-30e-3p	95.43769	108.1591	156.6103	-0.318041251	0.388119971	-0.047566763	0.470108729	0.387428707	0.896718685
	miR-30e-5p	3980.498	5316.009	4083.933	0.005039754	-0.588557834	-0.698231065	0.992284552	0.064920004	0.014443821
miR-31	*miR-31-3p*	3.048758	3.620677	23.13141	-1.149203865	3.273254706	2.084201017	0.219141356	0.002631256	0.003426581
	miR-31-5p	62.09992	20.74554	84.7239	-1.992230903	1.710284838	-0.399373598	0.002875447	0.00833668	0.332205161
miR-32	*miR-32-5p*	59.48924	97.72888	43.58729	0.397277481	-1.389713966	-1.10305878	0.452698792	0.026336602	0.029227486
miR-34	*miR-34a-5p*	54.75035	116.0908	117.5013	0.609862358	-0.225690874	0.263787487	0.24966743	0.72525135	0.601659418
	miR-34b-3p	21.10993	44.38301	195.1343	0.541854628	1.863268018	2.289009081	0.302810449	0.010412735	0.000904348
	miR-34b-5p	26.89205	24.93541	81.79152	-0.249204703	1.523747314	1.163824441	0.77436333	0.042105121	0.05057581
	miR-34c-3p	5.975808	12.17613	29.2718	0.517167919	0.663677942	1.062912391	0.359110676	0.48677938	0.152057678
	miR-34c-5p	550.2979	350.2299	1468.74	-0.915479192	1.855761913	0.813106768	0.094960173	0.00505084	0.068086783
miR-92	*miR-92a-3p*	151.2656	760.8631	165.9486	1.727848625	-2.274466839	-0.654443318	0.017126919	0.002920134	0.218603951
	miR-92b-3p	66.74959	21.58488	15.8389	-2.227358713	-0.559334878	-2.908508387	0.006328707	0.443495331	0.000220496
miR-93	*miR-93-5p*	414.8469	712.5817	371.3051	0.351517246	-1.127071577	-0.8948212	0.316546313	0.013093356	0.014663191
miR-96	*miR-96-5p*	152.5689	132.1778	137.0504	-0.711354016	-0.141847147	-0.974556485	0.309491606	0.869026521	0.102194083
miR-98	*miR-98-5p*	470.6453	659.1539	716.7859	0.019836183	-0.14742398	-0.238574434	0.975350039	0.755462435	0.493599636
miR-99	*miR-99a-3p*	24.71766	35.25727	35.48045	0.132108535	-0.173165235	-0.156048965	0.817362186	0.716618011	0.666187741
	miR-99a-5p	816.4637	1210.232	1450.953	0.097136021	0.018512865	0.015182773	0.900566621	0.963918147	0.972206355
	miR-99b-3p	23.47391	33.97495	21.49544	0.02641814	-0.786251748	-0.881328331	0.975350039	0.137278702	0.045180059
	miR-99b-5p	6972.371	6311.945	3309.46	-0.655015937	-1.152096455	-1.87922092	0.28868607	0.053276006	0.001372873
miR-100	*miR-100-5p*	1758.091	1522.739	1374.513	-0.720146085	-0.384038413	-1.1832474	0.28634416	0.567229172	0.033023219
miR-101	*miR-101a-3p*	1703.346	3911.329	3637.249	0.884081374	-0.284849193	0.47651418	0.04153974	0.48677938	0.147836454
	miR-101a-5p	5.643407	12.44724	14.4569	0.75376423	0.05913014	0.708397854	0.290474972	0.937218235	0.208297608
	miR-101b-3p	229.5357	537.3874	767.6716	0.903210403	0.280729577	1.065849387	0.036797683	0.524496478	0.009283949
	miR-101c	12.78008	59.78356	70.54364	1.926260943	-0.166012843	1.656804969	0.012072011	0.859790641	0.030124037
miR-103	*miR-103-3p*	723.5192	1558.45	1298.898	0.713049758	-0.544653827	0.054153243	0.045720764	0.190815187	0.877121454

miR-106	*miR-106b-3p*	11.8283	30.83669	41.03646	0.944293935	0.192470031	1.013869427	0.047719835	0.697051137	0.018998331
	miR-106b-5p	32.84491	68.65744	74.57822	0.738182612	-0.072534845	0.552993959	0.151092722	0.895715681	0.191771684
miR-107	*miR-107-3p*	189.6545	748.2991	505.8928	1.545036987	-0.862977014	0.563208229	0.002875447	0.058930576	0.135633805
miR-125	*miR-125a-5p*	3711.136	2944.162	1320.922	-0.843354484	-1.376087266	-2.319772809	0.047374064	0.009139276	6.21562E-05
	miR-125b-1-3p	22.87513	16.48787	28.37082	-0.878230493	0.491729598	-0.507993854	0.07295277	0.378336743	0.220751386
	miR-125b-2-3p	233.8079	280.7885	348.8163	-0.170516243	0.120611533	-0.168263285	0.73971136	0.78219141	0.601659418
	miR-125b-5p	2217.938	2384.297	1947.454	-0.364289731	-0.511248711	-0.980403358	0.285170322	0.177333515	0.004858766
miR-126	*miR-126a-3p*	374.0348	478.5326	635.6292	-0.020005009	0.185566744	0.0595939	0.985189775	0.800905139	0.917607616
	miR-126a-5p	1416.884	1903	2427.074	0.029820072	0.147918247	0.067932699	0.9622678	0.697051137	0.81905162
miR-127	*miR-127-3p*	1832.275	1776.347	3156.863	-0.403116507	0.641614695	0.121184768	0.356045024	0.113922292	0.691225054
	miR-127-5p	7.06052	11.85146	22.25129	0.283982569	0.814050684	0.99139239	0.653435069	0.142634786	0.032896282
miR-128	*miR-128-3p*	22.97636	24.04057	69.29681	-0.432093899	1.434273476	0.893034384	0.408744658	0.013093356	0.038574509
miR-130	*miR-130a-3p*	377.3828	656.1665	730.2551	0.353004642	-0.023826013	0.206373582	0.297716668	0.946786958	0.443795012
	miR-130b-3p	9.085641	8.423184	11.92362	-0.568310543	0.308494412	-0.379771507	0.285170322	0.587046454	0.379298287
miR-132	*miR-132-3p*	9.881275	19.80556	19.22878	0.539373132	-0.212677951	0.202374601	0.318147223	0.694659898	0.632530414
miR-133	*miR-133a-3p*	118.1007	127.2711	2437.686	-0.24250349	3.84209768	3.480555903	0.583584605	9.4164E-05	4.00293E-05
	miR-133a-5p	0.068913	0.292088	13.12576	0	4.412748276	5.917709366	0	0.00106635	5.30134E-05
	miR-133b-3p	7.437436	8.194677	277.1107	-0.140845699	4.638154778	4.388418901	0.855334899	9.4164E-05	1.49743E-05
miR-134	*miR-134-5p*	6.393365	10.05337	15.14469	0.159475039	0.548501756	0.601043297	0.868407987	0.422325664	0.245806966
miR-135	*miR-135a-2-3p*	23.21106	7.038828	5.572719	-2.18842977	-0.691640535	-3.016051918	0.006348111	0.4136917	0.000403896
	miR-135a-5p	275.0092	151.8239	100.7899	-1.195374744	-0.8180819	-2.132720462	0.012072011	0.093578006	0.000136541
	miR-135b-5p	7.379176	19.78865	2.223633	0.927092446	-3.153324416	-2.364122082	0.191837409	0.002703825	0.00449756
miR-136	*miR-136-3p*	117.9455	177.0343	257.1905	0.199016373	0.391394977	0.474622882	0.686432064	0.392636686	0.177773898
	miR-136-5p	77.52813	159.7694	246.8568	0.795379956	0.522524505	1.211951952	0.216916794	0.429294515	0.032896282
miR-137	*miR-137-3p*	0.167308	0.345936	30.11075	0	5.420975021	6.362450118	0	0.000379742	3.53206E-05
miR-138	*miR-138-5p*	87.23307	21.9489	11.45327	-2.24928504	-1.084955473	-3.450255886	0.008645822	0.172867286	0.000136541
miR-139	*miR-139-5p*	5.539646	14.86581	62.93709	0.889430978	1.896749758	2.672776417	0.100076548	0.006502718	0.000220496
miR-140	*miR-140-3p*	185.8509	262.7274	301.8849	0.04471272	0.019972233	-0.055084449	0.929715171	0.951585042	0.866514879
	miR-140-5p	9.97818	8.671298	18.78739	-0.306784317	0.73528201	0.332840642	0.725738828	0.399004793	0.611957743
miR-141	*miR-141-3p*	17881.81	65693.72	40435.27	1.364139772	-0.889724893	0.375471019	0.009630229	0.019402306	0.186155062
	miR-141-5p	54.3428	85.69912	64.50423	0.319860123	-0.679865938	-0.47250665	0.524195061	0.230002767	0.285192754
miR-142	*miR-142a-3p*	21.49146	50.51027	94.24045	0.878492655	0.655962454	1.420749214	0.119850613	0.358797791	0.022574041
	miR-142a-5p	249.9898	631.9118	539.4102	0.791344163	-0.611951245	0.04580252	0.156046197	0.48677938	0.951420475
miR-143	*miR-143-3p*	62956.09	99957.76	315271.4	0.184409663	1.477323433	1.549599651	0.66276728	0.000248056	4.18184E-05
	miR-143-5p	11.35576	8.293334	31.59593	-0.660473011	1.548077959	0.769334307	0.317076002	0.060093864	0.202315757
miR-144	*miR-144-3p*	4.537457	8.286416	13.14576	0.964924818	0.596542419	1.503907062	0.354425211	0.547885002	0.074041827
miR-145	*miR-145a-3p*	195.9119	244.7847	1342.89	-0.124870039	2.102194285	1.867778584	0.879289594	0.006469162	0.003205877
	miR-145a-5p	432.5407	423.9325	1850.243	-0.431378412	1.684701793	1.135289563	0.284111989	0.03691876	0.06444401
	miR-145b	3.415079	3.405091	12.27909	-0.177397106	1.431971951	1.093441226	0.858892058	0.095511715	0.081498889
miR-146	*miR-146a-5p*	246.7639	486.2349	198.3022	0.647750723	-1.357294736	-0.836420951	0.505781669	0.125575504	0.233349697
	miR-146b-5p	4425.801	771.2271	292.749	-2.946426198	-1.533199773	-4.559731661	0.007675979	0.09932902	4.18184E-05
miR-148	*miR-148a-3p*	43756.86	33009.5	13581.39	-0.846831509	-1.480638667	-2.442544571	0.010170527	9.4164E-05	3.41801E-07
	miR-148a-5p	240.9846	115.9316	55.51084	-1.525687999	-1.222023657	-2.866470174	0.009371704	0.027508366	2.87377E-05
	miR-148b-3p	708.0497	546.0564	617.3286	-0.857449503	0.027334661	-0.948840772	0.061030138	0.947802093	0.014948869
	miR-148b-5p	15.04882	17.57213	16.7535	-0.16847745	-0.277938865	-0.562198213	0.771981454	0.575386455	0.152057678
miR-149	*miR-149-5p*	91.46385	28.33534	51.60283	-2.034800444	0.65848456	-1.491388269	0.003635076	0.233389691	0.003109428
miR-150	*miR-150-5p*	38.80415	51.64504	64.06865	-0.039403503	-0.028073415	-0.185970274	0.948958325	0.958575357	0.713095054
miR-151	*miR-151-3p*	777.6656	754.0996	810.4877	-0.512166128	-0.060265199	-0.687323284	0.321724744	0.903419035	0.069821741
	miR-151-5p	694.1105	648.3303	704.7819	-0.583067871	-0.084294253	-0.772604041	0.219141356	0.865938467	0.033112664
miR-152	*miR-152-3p*	64.41694	201.5415	300.6728	1.169076759	0.389701864	1.442909573	0.026374801	0.422325664	0.002702948
	miR-152-5p	40.97624	37.26235	57.91944	-0.559121997	0.393593122	-0.286328941	0.214688025	0.449030635	0.463920629
miR-153	*miR-153-3p*	9.004047	14.62848	5.441267	0.327224891	-1.635972495	-1.433563397	0.561313156	0.016626021	0.011028843
miR-155	*miR-155-5p*	2.299638	3.420827	12.98107	0.132892021	1.698990524	1.74358285	0.900566621	0.022269424	0.00452839
miR-181	*miR-181a-1-3p*	24.01619	32.59831	44.43472	-0.02190806	0.243558872	0.105983675	0.976628397	0.596489856	0.772239831
	miR-181a-5p	6010.318	5803.209	5580.362	-0.540000976	-0.208184453	-0.869485377	0.121261929	0.524496478	0.004325586
	miR-181b-5p	257.0349	269.9029	287.757	-0.348410722	-0.133413116	-0.6046154	0.383810349	0.799132993	0.113078502
	miR-181c-3p	227.1023	189.4173	159.8382	-0.692443462	-0.445927137	-1.256280392	0.083369004	0.302342064	0.002134078
	miR-181c-5p	8487.392	10718.85	11327.98	-0.09678751	-0.109460113	-0.324222262	0.801268528	0.72525135	0.169495583
	miR-181d-5p	467.1343	597.4812	634.0425	-0.088228694	-0.129707002	-0.335829103	0.858892058	0.741851554	0.24957439
miR-182	*miR-182-5p*	13075.37	6768.854	3866.686	-1.436719614	-0.997674306	-2.540252154	0.003635076	0.003803587	7.9722E-07
miR-183	*miR-183-3p*	16.09919	15.16323	8.200586	-0.54013566	-1.124161379	-1.782951893	0.27852011	0.053359177	0.001691302
	miR-183-5p	1892.453	1122.098	684.7448	-1.220274803	-0.951926692	-2.266737454	0.032860997	0.060888936	0.000134976
miR-184	*miR-184-3p*	3.802994	51.326	95.23449	3.30591811	0.335153723	3.491704658	0.002875447	0.71719014	0.000904348
miR-185	*miR-185-5p*	8.817988	25.77594	10.64446	1.062116383	-1.650994947	-0.718503024	0.12220354	0.054341622	0.285169753
miR-186	*miR-186-5p*	586.7567	1211.291	775.1018	0.556401259	-0.81386806	-0.379091509	0.126837895	0.036217923	0.198646673
miR-187	*miR-187-3p*	101.4671	176.4676	67.02443	0.331538111	-1.620882305	-1.408896082	0.396071696	0.002703825	0.001797627
miR-190	*miR-190a-5p*	11.91518	134.8068	89.45522	3.327196263	-0.941204787	2.289663694	0.002875447	0.246580727	0.006109794
miR-191	*miR-191-5p*	18876.23	9767.802	6189.365	-1.425105545	-0.809327234	-2.327443899	0.03946427	0.189469016	0.000429921
miR-192	*miR-192-5p*	1125.937	428.9003	717.1246	-1.887226849	0.535118379	-1.461464712	0.003635076	0.230085792	0.000841377
miR-193	*miR-193a-3p*	6.40249	9.210664	25.99525	0.397732245	1.340577404	1.647745643	0.666552668	0.091777666	0.016366765
	miR-193b-3p	5.742751	12.34382	34.03359	0.645607473	1.384422504	1.926569166	0.285170322	0.022269424	0.00110455
miR-194	*miR-194-5p*	49.50083	32.41565	50.6804	-1.059177576	0.429541391	-0.745099622	0.028540075	0.396797428	0.060654992
miR-195	*miR-195a-5p*	267.7593	432.821	635.0468	0.163015777	0.397571999	0.446488784	0.801268528	0.400179552	0.215089864
miR-196	*miR-196a-5p*	5.352488	82.43324	328.4753	3.516618101	1.59726828	4.996968169	0.000367545	0.028614462	1.50977E-05
	miR-196b-5p	7.090534	93.88694	1045.887	3.360682576	3.159676778	6.409709598	0.000266352	9.4164E-05	2.74047E-07
miR-199	*miR-199a-3p*	1375.303	3026.257	4773.963	0.730824843	0.489479602	1.098434802	0.047719835	0.160556599	0.001880512
	miR-199a-5p	336.5354	636.8304	536.7103	0.506253434	-0.454165353	-0.064209272	0.128166585	0.200212537	0.825306144
	miR-199b-3p	1360.119	3006.432	4742.96	0.737211706	0.490026978	1.105297705	0.047374064	0.160434895	0.001797627
	miR-199b-5p	53.8121	104.9248	185.0064	0.627680345	0.681707013	1.198914348	0.244549848	0.186816077	0.01150529
miR-200	*miR-200a-3p*	2153.815	4627.942	3498.639	0.679255503	-0.59297571	-0.03083209	0.045398785	0.064532605	0.911619436
	miR-200a-5p	43.32879	49.72854	54.95542	-0.265557161	-0.032039974	-0.417318449	0.561313156	0.946786958	0.264425455
	miR-200b-3p	3667.632	2177.383	2424.789	-1.166151403	-0.102906988	-1.373524241	0.012345269	0.839102283	0.001968322
	miR-200b-5p	24.92871	16.02568	24.81381	-1.098511315	0.380477882	-0.83668065	0.044117679	0.488472291	0.054834853
	miR-200c-3p	1263.115	1835.269	1441.26	0.068053919	-0.531016304	-0.574031523	0.900566621	0.12314076	0.042256765
miR-203	*miR-203-3p*	393.3638	401.6329	337.6223	-0.409973215	-0.614776337	-1.145706002	0.243533606	0.340295248	0.030117232
miR-204	*miR-204-5p*	11488.45	5777.05	366.3336	-1.526630963	-4.200025292	-5.829837451	0.019371399	9.4164E-05	1.31718E-06

miR-205	*miR-205-5p*	1495.877	1498.252	1372.105	-0.461477441	-0.357467721	-0.930506177	0.24966743	0.425029009	0.015307205
miR-208	*miR-208b-3p*	0.120002	0.399644	31.24887	0	5.975927058	7.21623199	0	0.000148216	2.70082E-05
miR-210	*miR-210-3p*	435.8675	856.2051	337.609	0.562010754	-1.536213786	-1.09706974	0.12220354	0.002703825	0.005716353
	miR-210-5p	15.27444	17.95189	7.109624	-0.293777663	-1.447829585	-1.849515928	0.603506967	0.053276006	0.005904828
miR-211	*miR-211-5p*	24.30312	15.71972	2.076014	-1.028772343	-3.326969235	-4.479308099	0.061030138	0.00153238	3.53206E-05
miR-214	*miR-214-3p*	61.25059	95.30477	72.89317	0.289747894	-0.613746372	-0.443307793	0.557054292	0.243803579	0.28334743
	miR-214-5p	17.55836	32.13787	22.38688	0.529257898	-0.751675189	-0.340956318	0.294544114	0.218340245	0.482850926
miR-218	*miR-218-5p*	40.91138	110.7421	77.05944	1.151678638	-0.731959729	0.309325591	0.034867786	0.173617173	0.476965176
miR-221	*miR-221-3p*	305.339	1052.386	2516.147	1.325556422	1.018267338	2.227844631	0.006328707	0.016117252	3.53206E-05
	miR-221-5p	14.44815	9.987044	17.22426	-0.99321221	0.549216418	-0.580861592	0.171628117	0.477516541	0.291565233
miR-222	*miR-222-3p*	149.9543	230.237	700.1075	0.177753425	1.307862481	1.368472535	0.686432064	0.012826827	0.002651882
miR-223	*miR-223-3p*	28.2866	67.57227	109.5743	0.75704868	0.439511614	1.077416675	0.0991395	0.47012944	0.036096126
miR-296	*miR-296-5p*	10.0462	5.249775	4.385772	-1.24585081	-0.521452953	-1.874106309	0.043063978	0.54806622	0.010456713
miR-298	*miR-298-5p*	13.74125	5.257168	4.61636	-1.670896253	-0.445065862	-2.270712381	0.022039895	0.558020798	0.001349533
miR-299	*miR-299a-3p*	5.688252	8.070134	15.22633	0.141517736	0.787226052	0.832171133	0.877403127	0.229911929	0.105226215
	miR-299a-5p	3.34713	3.213583	11.79629	-0.551962948	1.63513237	0.966766561	0.452698792	0.042076192	0.082158619
miR-300	*miR-300-3p*	53.4806	77.10407	103.3384	0.127028537	0.198534826	0.209137294	0.838607451	0.738840222	0.644868814
miR-301	*miR-301a-3p*	131.7526	173.1029	174.6123	0.090989421	-0.189451163	-0.21532672	0.899643469	0.708762026	0.589235172
miR-320	*miR-320-3p*	111.0604	182.6395	106.6319	0.237604374	-0.911343769	-0.794254878	0.594715135	0.048145922	0.035792835
miR-322	*miR-322-3p*	21.55978	35.57597	12.82612	0.147948312	-1.603270912	-1.561708561	0.858892058	0.042076192	0.019849379
	miR-322-5p	135.2818	83.88256	67.34246	-1.057814954	-0.486866214	-1.663245652	0.03946427	0.366291628	0.001404461
miR-324	*miR-324-3p*	8.64388	19.55111	6.98348	0.811096557	-1.627040102	-0.941435486	0.162368445	0.020029453	0.080541491
	miR-324-5p	17.40278	22.67343	18.79468	-0.012916647	-0.497042785	-0.632894438	0.985189775	0.378336743	0.148859287
miR-326	*miR-326-3p*	8.598849	11.36884	25.27767	0.120564161	0.833204064	0.845471889	0.888003869	0.229549524	0.119068774
miR-328	*miR-328-3p*	34.83724	44.72594	51.90303	-0.085151085	0.080788085	-0.127642907	0.909856473	0.891384799	0.756872197
miR-329	*miR-329-3p*	2.459132	4.004864	13.0595	0.259370515	1.49113012	1.620252298	0.755514193	0.029742826	0.004923797
	miR-329-5p	4.069354	7.123116	10.96208	0.280980524	0.59518641	0.762592499	0.713975653	0.377852859	0.138513137
miR-330	*miR-330-5p*	34.70345	45.01699	14.46536	-0.016746943	-1.827207225	-1.960098975	0.983183649	0.002763775	0.000475923
miR-331	*miR-331-3p*	35.63768	65.62305	31.38441	0.513139328	-1.533363228	-1.139234588	0.309491606	0.053276006	0.07706087
miR-335	*miR-335-3p*	16.48648	45.20008	142.2305	1.201042874	1.393838555	2.478179639	0.047374064	0.029742826	0.000403896
	miR-335-5p	17.96152	56.55038	72.50959	1.177611523	0.125415723	1.188627647	0.095841392	0.895155059	0.092667914
miR-337	*miR-337-3p*	2.0359	3.789752	10.45144	0.595071077	1.092770172	1.568269818	0.349100303	0.115258236	0.009932795
	miR-337-5p	6.142498	6.388023	13.28974	-0.337821991	0.946416846	0.519077203	0.606786873	0.129611394	0.26018304
miR-338	*miR-338-3p*	20.40091	19.65597	59.20854	-0.455099065	1.424740689	0.839508636	0.480709571	0.037893668	0.102326045
	miR-338-5p	1.184517	1.68342	23.70654	-0.054461445	3.53185379	3.399549702	0.973345036	0.002335512	0.000310161
miR-339	*miR-339-3p*	18.72693	13.41215	11.92355	-0.863736226	-0.371934759	-1.350995092	0.061786123	0.501560764	0.006025689
	miR-339-5p	34.89429	36.37358	54.80411	-0.337109552	0.364434131	-0.091386983	0.459292952	0.470553774	0.81905162
miR-340	*miR-340-3p*	17.97891	11.09257	14.42094	-1.079175279	0.099101779	-1.102369082	0.047719835	0.884476115	0.026074453
	miR-340-5p	950.5084	855.0117	1068.895	-0.454970155	0.150773065	-0.431352328	0.272740614	0.738318252	0.191771684
miR-341	*miR-341-3p*	11.13503	14.88832	28.55441	0.158936549	0.739047837	0.781765918	0.826856043	0.169676321	0.072358501
miR-342	*miR-342-3p*	25.6759	42.27716	101.8948	0.138626056	1.171456567	1.199474342	0.888003869	0.052854874	0.019320302
miR-345	*miR-345-3p*	23.30342	33.55413	40.67538	0.108649678	0.11397022	0.100162234	0.877403127	0.839102283	0.806479154
	miR-345-5p	99.65213	203.053	226.3865	0.597113934	-0.035807487	0.440816655	0.186291761	0.937218235	0.204293606
miR-350	*miR-350-3p*	7.974353	14.87415	70.68275	0.118157244	2.340877185	2.357763591	0.903501695	0.00267053	0.000515264
miR-351	*miR-351-5p*	241.3481	183.9482	86.12002	-0.872218234	-1.31614941	-2.30614797	0.047050548	0.020029453	0.000137075
miR-361	*miR-361-3p*	18.05997	31.8408	18.77439	0.325643835	-0.958585649	-0.7524181	0.504734688	0.086413463	0.095673866
	miR-361-5p	78.26481	77.35979	83.46044	-0.502696835	-0.100328231	-0.725096842	0.342218011	0.859790641	0.078022274
miR-362	*miR-362-3p*	11.68877	19.14716	54.34345	0.450515051	1.259763277	1.607753542	0.407185213	0.044768784	0.00490564
miR-365	*miR-365-3p*	11.84072	15.40615	45.29328	-0.051543509	1.393524758	1.231218461	0.948958325	0.020029453	0.01150529
miR-369	*miR-369-5p*	4.097413	4.527325	10.3842	-0.229934988	0.843303168	0.481134003	0.733509258	0.172867286	0.277427257
miR-374	*miR-374b-5p*	26.25672	61.83408	180.6559	0.63085473	1.422368216	1.932375417	0.330504631	0.035351667	0.002611952
miR-375	*miR-375-3p*	1340.823	856.669	80.07612	-1.256979147	-3.57354789	-4.958740079	0.026217346	0.000139501	2.0214E-06
miR-376	*miR-376b-3p*	3.79663	5.272904	16.13274	0.156096338	1.341818538	1.372202442	0.830843854	0.022269424	0.004858766
miR-378	*miR-378a-3p*	455.1536	402.6514	1015.812	-0.540822131	1.065270011	0.404892646	0.118662164	0.020029453	0.20252865
	miR-378a-5p	4.53654	4.565158	17.14738	-0.303557843	1.585484645	1.131012355	0.686432064	0.030008884	0.035471674
	miR-378c	131.8762	102.2791	248.1714	-0.760630251	1.007881729	0.128419852	0.06943281	0.041729753	0.720365695
	miR-378d	9.805047	11.25369	68.71744	-0.180158585	2.216728216	1.920504463	0.756453508	0.003567184	0.002134078
miR-379	*miR-379-5p*	5.996797	10.2386	26.20113	0.471971917	1.105702224	1.468569289	0.3707792	0.053276006	0.004858766
miR-381	*miR-381-3p*	39.8308	64.48808	83.00732	0.299632746	0.253570001	0.436097921	0.634264047	0.67832875	0.350796521
miR-409	*miR-409-3p*	14.5097	17.01011	36.52041	-0.212894513	0.924093867	0.598118443	0.706863981	0.053276006	0.104974429
	miR-409-5p	5.151606	7.219142	20.43665	0.069888213	0.858443535	0.804501458	0.941676611	0.363008735	0.245806966
miR-410	*miR-410-3p*	51.42353	106.4239	169.9145	0.669822453	0.582863438	1.134350829	0.28634416	0.312982083	0.023898962
miR-411	*miR-411-5p*	592.5973	684.9646	1134.632	-0.133989896	0.521453214	0.266927312	0.758698352	0.179014194	0.369506921
miR-421	*miR-421-3p*	46.22756	77.2776	68.91632	0.272348914	-0.408235316	-0.254594033	0.603506967	0.489848696	0.595467525
miR-423	*miR-423-3p*	124.2404	91.74414	72.61324	-0.8794495	-0.478113063	-1.476331956	0.051202694	0.347569735	0.002164568
	miR-423-5p	71.82507	65.04885	39.5295	-0.617516225	-0.887413068	-1.624064293	0.151092722	0.056458594	0.000771869
miR-425	*miR-425-3p*	12.6221	12.75286	9.568881	-0.443641878	-0.660046193	-1.226305003	0.473261495	0.289525895	0.019553916
	miR-425-5p	337.3207	445.7431	357.9582	-0.068211439	-0.500837645	-0.686532652	0.90111617	0.229549524	0.044816576
miR-429	*miR-429-3p*	3063.889	4342.476	3871.603	0.074867063	-0.382844606	-0.418543965	0.889843693	0.3118748	0.153096266
miR-431	*miR-431-5p*	7.065724	6.264592	16.94792	-0.40994236	1.177008528	0.658382542	0.49149634	0.065597092	0.165991442
miR-434	*miR-434-3p*	241.2076	283.2168	1045.757	-0.160505977	1.772783169	1.495259263	0.803532177	0.00280922	0.002134078
	miR-434-5p	77.83643	110.3063	117.308	0.080265813	-0.358508021	-0.397565463	0.900566621	0.622753475	0.491936155
miR-449	*miR-449a-5p*	16.21762	22.34128	44.48461	-0.037939109	0.890791803	0.743393111	0.968716112	0.093578006	0.081490726
miR-450	*miR-450a-5p*	20.63958	29.45788	18.71121	-0.008992214	-0.891879914	-1.00518866	0.993060774	0.229549524	0.093330702
	miR-450b-5p	10.69722	6.135078	5.096641	-1.223914074	-0.525241283	-1.828823394	0.100076548	0.558083602	0.014948869
miR-451	*miR-451a*	39.04081	197.3896	196.2578	1.714413468	-0.214491591	1.384194813	0.048197555	0.845363294	0.103968606
miR-455	*miR-455-3p*	16.32762	17.01068	10.55638	-0.42552138	-0.639702955	-1.183298917	0.653435069	0.48677938	0.09918245
	miR-455-5p	11.66082	20.23929	23.83927	0.573984534	0.04649926	0.52925103	0.383810349	0.946786958	0.363470272
miR-463	*miR-463-5p*	1.909212	12.03655	20.34676	2.473874163	0.495895339	2.808977124	0.018575293	0.552720424	0.002386355
miR-465	*miR-465a-3p*	6.847488	24.34552	37.80896	1.299828388	0.46825026	1.631309759	0.045306939	0.458474602	0.006109794
	miR-465a-5p	3.429146	15.46594	29.99413	1.734268957	0.730860998	2.325198563	0.013085056	0.185874673	0.000392737
	miR-465b-3p	6.847488	24.34552	37.80896	1.299828388	0.46825026	1.631309759	0.045306939	0.458474602	0.006109794
	miR-465b-5p	3.197165	7.771708	107.371	0.885337062	3.617168242	4.325277467	0.292773543	0.000622811	3.53206E-05
	miR-465c-3p	6.808056	24.34552	37.59984	1.309072486	0.458815149	1.63080616	0.045306939	0.471695842	0.006460002
	miR-465c-5p	20.50765	131.1992	175.9528	2.202483176	0.246554982	2.332598068	0.004351938	0.642167943	0.000306201
miR-466	*miR-466a-3p*	12.56781	4.973701	3.328173	-1.817022345	-0.594280388	-2.501253114	0.009657144	0.401310736	0.000429921

	miR-466b-3p	13.5434	4.489491	3.497758	-1.93371957	-0.482266381	-2.519548252	0.008185607	0.489848696	0.000398166
	miR-466c-3p	13.5434	5.135244	3.497758	-1.844198327	-0.582754316	-2.519548252	0.009657144	0.422325664	0.000475923
	miR-466e-3p	13.58283	5.135244	3.497758	-1.847097872	-0.582754316	-2.522438416	0.009657144	0.422325664	0.000475923
	miR-466i-5p	9.264145	22.28997	25.24016	1.508103202	0.225121581	1.605700178	0.285170322	0.869785614	0.120094271
	miR-466p-3p	13.5434	5.135244	3.497758	-1.844198327	-0.582754316	-2.519548252	0.009657144	0.422325664	0.000475923
miR-467	*miR-467a-3p*	14.7378	6.088762	7.316219	-1.487084931	-0.099089912	-1.712918895	0.034867786	0.913008489	0.018704597
	miR-467a-5p	73.44441	36.44021	15.81497	-1.435964842	-1.387357977	-2.943570929	0.009371704	0.015211197	2.70082E-05
	miR-467b-5p	73.44441	36.44021	15.81497	-1.435964842	-1.387357977	-2.943570929	0.009371704	0.015211197	2.70082E-05
	miR-467c-5p	20.57138	7.27051	4.001056	-1.765498557	-0.996999665	-2.90014136	0.017126919	0.238749534	0.000675158
	miR-467d-3p	14.7378	6.088762	7.316219	-1.487084931	-0.099089912	-1.712918895	0.034867786	0.913008489	0.018704597
	miR-467d-5p	34.51165	13.34098	6.483751	-1.810642068	-1.156352526	-3.083887559	0.006328707	0.056458594	4.00293E-05
	miR-467e-5p	26.39508	13.22106	8.655821	-1.282070735	-0.831401094	-2.227521608	0.021049077	0.160556599	0.000403896
miR-470	*miR-470-3p*	0.255752	1.414461	11.49748	2.284826023	2.122520774	4.277442459	0.062644401	0.077448255	0.003472757
	miR-470-5p	53.06473	205.9001	193.9103	1.547716002	-0.239754861	1.170118826	0.061030138	0.755481378	0.06444401
miR-471	*miR-471-5p*	0.36495	2.675247	18.00846	2.253294708	2.53954592	4.693378658	0.038649592	0.005824226	0.000136519
miR-484	*miR-484*	54.45917	55.32394	34.47672	-0.429851176	-0.898031985	-1.449497308	0.325064704	0.082122693	0.00308914
miR-486	*miR-486a-3p*	0.109198	1.091237	13.5933	0	4.149945349	5.611207669	0	0.002335512	0.000237361
	miR-486a-5p	230.5439	302.5128	1748.681	-0.076577994	2.480641214	2.272404386	0.945294712	0.002756201	0.001124116
	miR-486b-5p	230.0478	302.5357	1743.355	-0.074340599	2.476964307	2.270927622	0.945933528	0.002756201	0.001124116
miR-496	*miR-496a-3p*	2.424274	3.290281	17.03047	-0.074385839	2.110034752	1.919874812	0.943510704	0.009139276	0.002651882
miR-497	*miR-497a-5p*	189.2301	142.9085	204.6781	-0.621782036	0.292461869	-0.448459249	0.321724744	0.682166459	0.396405263
miR-500	*miR-500-3p*	12.17856	14.73376	31.94023	-0.092944825	0.910269552	0.693612758	0.911061184	0.096980488	0.106068672
miR-501	*miR-501-3p*	45.3801	55.24287	105.7755	-0.180524463	0.687033706	0.383126717	0.846375559	0.304732052	0.454616759
miR-511	*miR-511-3p*	5.164833	16.75047	17.95116	1.356932079	-0.115226183	1.134414281	0.028632265	0.869785614	0.042256765
miR-532	*miR-532-3p*	9.189834	9.27749	12.6254	-0.452782659	0.179227631	-0.384215011	0.361351454	0.806857341	0.456714271
	miR-532-5p	77.42859	136.9847	212.333	0.352093638	0.471284171	0.705191557	0.383810349	0.246580727	0.041622111
miR-541	*miR-541-5p*	103.8572	85.95655	178.9424	-0.703908137	0.856088946	0.03296185	0.126404766	0.05998791	0.931257916
miR-542	*miR-542-3p*	9.462756	11.41515	6.568334	-0.272195573	-0.865142378	-1.230978023	0.686432064	0.189469016	0.02759367
miR-574	*miR-574-3p*	38.36298	72.90026	88.53324	0.483945979	0.053815386	0.414648816	0.318147223	0.917461252	0.300753111
	miR-574-5p	19.39668	37.78556	39.48864	0.508238838	-0.213349896	0.175508901	0.27852011	0.694659898	0.673633275
miR-582	*miR-582-3p*	56.77628	21.44155	20.45072	-1.824331806	-0.282879396	-2.221653932	0.006328707	0.681600739	0.000904348
	miR-582-5p	19.77716	7.565217	15.52575	-1.675861112	0.769955262	-1.026996441	0.015659323	0.24105919	0.041642004
miR-598	*miR-598-3p*	9.506166	24.44258	7.506788	0.897489888	-1.865213497	-1.092978494	0.076749117	0.002920134	0.021438366
miR-615	*miR-615-3p*	15.71335	32.67875	23.50485	0.582755695	-0.674403377	-0.214498727	0.262377366	0.218725957	0.632034927
miR-652	*miR-652-3p*	193.9849	259.0896	238.4083	-0.062096782	-0.310530786	-0.494074746	0.914908366	0.452200482	0.121079973
miR-664	*miR-664-3p*	12.29089	20.11503	26.14588	0.180528998	0.202362212	0.266221178	0.855334899	0.798463915	0.644868814
miR-669	*miR-669a-3p*	39.9037	18.30356	9.469822	-1.407561188	-1.354493359	-2.879644016	0.015795739	0.12314076	0.001075503
	miR-669a-5p	21.39108	11.4209	12.66436	-1.343608522	-0.081246675	-1.550248775	0.026217346	0.895715681	0.004050994
	miR-669c-5p	17.92877	5.97948	3.768364	-1.9660273	-0.926583099	-3.014913906	0.004351938	0.152006916	6.25317E-05
	miR-669o-3p	39.9037	18.30356	9.469822	-1.407561188	-1.354493359	-2.879644016	0.015795739	0.12314076	0.001075503
	miR-669p-5p	21.39108	11.4209	12.66436	-1.343608522	-0.081246675	-1.550248775	0.026217346	0.895715681	0.004050994
miR-671	*miR-671-3p*	20.59709	4.749273	3.576813	-2.583352273	-0.497238834	-3.194716488	0.002875447	0.449030635	3.53206E-05
miR-672	*miR-672-5p*	190.1177	193.948	98.25673	-0.461716345	-1.146386603	-1.729433618	0.299429752	0.026518159	0.000675158
miR-674	*miR-674-3p*	13.40435	7.032883	10.59045	-1.306043104	0.359138039	-1.051945376	0.022890264	0.578160026	0.041091095
miR-676	*miR-676-3p*	58.24567	101.94	157.1327	0.286260781	0.506970593	0.675809731	0.557594977	0.246580727	0.064601064
	miR-676-5p	7.102043	15.28699	24.72594	0.588624198	0.603016056	1.088954615	0.309125217	0.299558275	0.02759367
miR-677	*miR-677-3p*	4.696257	22.0242	5.730978	1.541156522	-2.015022139	-0.601528301	0.072137913	0.061018313	0.49505303
	miR-677-5p	46.81577	176.1328	77.26969	1.26593939	-1.132778131	0.01816173	0.047719835	0.060888936	0.973644958
miR-690	*miR-690*	441.1346	1003.088	525.7527	0.742493658	-1.07929763	-0.459411963	0.146734161	0.044768784	0.263404357
miR-708	*miR-708-3p*	3.880439	14.52196	24.19529	1.57052167	0.528277652	1.979237828	0.019371399	0.363008735	0.001249482
	miR-708-5p	4.94373	22.29021	39.26868	1.844678892	0.485548082	2.214858929	0.009657144	0.48677938	0.002254706
miR-709	*miR-709*	6.03368	26.29295	17.37678	1.976377219	-0.611136278	1.222437935	0.061030138	0.524496478	0.130227391
miR-714	*miR-714*	16.06179	12.58215	5.796385	-0.446428644	-0.743301228	-1.361958677	0.858892058	0.694780936	0.322787191
miR-741	*miR-741-3p*	17.05662	63.11928	65.61463	1.517137997	-0.060977916	1.339556209	0.123093173	0.946786958	0.081192996
miR-743	*miR-743a-3p*	1.88957	11.73079	15.36947	2.143905428	0.052088269	2.060268104	0.009651841	0.946786958	0.010491823
	miR-743b-3p	18.559	91.66415	95.84605	1.856965167	-0.293384597	1.445721742	0.007537817	0.67137707	0.020462855
miR-744	*miR-744-5p*	300.4944	150.435	101.2629	-1.451898673	-0.733699606	-2.308880385	0.008847953	0.169205948	0.000136541
miR-871	*miR-871-3p*	58.08885	179.5167	158.7277	1.213763644	-0.387875383	0.699207449	0.100224923	0.561048457	0.199814983
	miR-871-5p	5.172954	20.29122	41.63701	1.514501595	0.792564943	2.182288039	0.022039895	0.199806152	0.001124116
miR-872	*miR-872-3p*	55.47269	46.73851	73.83139	-0.619841923	0.463171149	-0.268175957	0.177924508	0.34203607	0.466114549
	miR-872-5p	222.8319	294.2147	308.9897	0.025959968	-0.22355546	-0.313853015	0.973345036	0.682166459	0.4486828
miR-874	*miR-874-3p*	18.28017	16.1942	17.26974	-0.633095571	-0.131113899	-0.884359229	0.285170322	0.838575725	0.056049358
miR-878	*miR-878-5p*	3.809844	27.09462	37.26621	2.324886533	0.204924039	2.403132768	0.004033908	0.765644344	0.001049403
miR-879	*miR-879-5p*	14.30882	1.583871	2.141639	-3.195582403	-0.161119956	-3.440234771	0.02762738	0.923059751	0.005112921
miR-880	*miR-880-3p*	1.187446	9.807819	12.0315	2.562649876	-0.042424915	2.373333359	0.003635076	0.950520016	0.004447004
miR-881	*miR-881-3p*	28.4658	187.8362	307.9376	2.200011534	0.55907113	2.637080011	0.003635076	0.261070917	7.7977E-05
miR-883	*miR-883a-3p*	3.079402	13.05834	14.30019	1.714945758	-0.138760652	1.462216102	0.014166523	0.859548313	0.022970715
miR-1195	*miR-1195*	18.23235	84.9543	55.94025	1.93964596	-0.69214038	1.124411054	0.045306939	0.439915273	0.125814887
miR-1198	*miR-1198-5p*	22.82497	34.37024	29.41514	0.092214444	-0.42992904	-0.456172807	0.900566621	0.404240253	0.24957439
miR-1247	*miR-1247-5p*	39.78441	41.62806	40.13181	-0.409350269	-0.370935352	-0.906923761	0.452698792	0.610624407	0.120856706
miR-1249	*miR-1249-3p*	14.83017	11.73707	16.81759	-0.763341854	0.387915335	-0.485310427	0.285170322	0.575386455	0.358772682
miR-1251	*miR-1251-5p*	20.18441	22.39947	13.97019	-0.214109421	-0.906140633	-1.229249855	0.723069318	0.179014194	0.031999213
miR-1291	*miR-1291*	2.982379	3.520793	11.90794	0.094951238	0.772954541	0.737960979	0.924445576	0.507309903	0.381642308
miR-1839	*miR-1839-3p*	27.41816	70.60384	19.65141	0.877036472	-2.03094992	-1.273325907	0.07510271	0.001648535	0.007151157
	miR-1839-5p	272.129	467.7826	406.783	0.343801979	-0.455574609	-0.230254394	0.38148337	0.332923093	0.529649737
miR-1843	*miR-1843a-5p*	83.71062	76.9753	55.07959	-0.527140007	-0.685779396	-1.330798186	0.186291761	0.128693969	0.002386355
	miR-1843b-3p	10.54681	8.429882	6.914574	-0.820696636	-0.427174414	-1.366274772	0.158787468	0.56826293	0.02790121
	miR-1843b-5p	105.7617	115.2725	90.61447	-0.298867832	-0.551988652	-0.970073296	0.447660494	0.176177661	0.007963809
miR-1957	*miR-1957a*	12.79337	12.91304	208.8254	0.138286979	3.550629652	3.548340273	0.926092522	0.010470985	0.002611952
miR-1981	*miR-1981-3p*	8.707576	11.76056	7.583326	-0.031303248	-0.780337739	-0.95382706	0.977221174	0.289179405	0.096355528
	miR-1981-5p	6.645359	18.4758	18.97706	0.984673227	-0.112054016	0.748065832	0.063730372	0.839102283	0.077266314
miR-3068	*miR-3068-3p*	102.8751	61.79112	25.14172	-1.195635576	-1.374619383	-2.68839653	0.045306939	0.040934463	0.000220496
miR-3470	*miR-3470a*	203.5008	760.5848	318.7918	1.742111314	-1.456473717	0.168083968	0.010622059	0.015211197	0.71182401
	miR-3470b	258.7032	1059.888	312.2792	1.832952514	-1.945498156	-0.235414852	0.012072011	0.004198643	0.632938028
miR-3473	*miR-3473a*	23.20783	114.5563	68.32337	1.929432738	-0.903018086	0.907021526	0.006328707	0.09932902	0.057191798
	miR-3473b	42.26793	219.3123	161.3538	2.050141124	-0.537384577	1.381876015	0.024829995	0.48677938	0.042537925

	miR-3473e	35.84598	198.1117	150.2287	2.066152859	-0.440317669	1.496756692	0.028180986	0.578160026	0.037893554
	miR-3473f	4.635255	33.71874	22.71387	2.360265643	-0.221326702	1.947355624	0.146734161	0.895155059	0.124901375
miR-3535	*miR-3535*	28.98144	349.291	66.84868	2.740884499	-2.231954215	0.374767821	0.009651841	0.013093356	0.597324188
miR-5099	*miR-5099*	143.0932	98.4456	161.5296	-0.826398245	0.395656329	-0.552132718	0.100224923	0.542800189	0.256825235
miR-5100	*miR-5100*	11.93236	8.339649	40.67933	0.075924667	1.521306572	1.475885424	0.973345036	0.32041671	0.199262978
miR-5119	*miR-5119*	1.957666	1.360474	26.07558	-0.091209467	2.758751252	2.557904693	0.973345036	0.232503943	0.120856706
miR-5121	*miR-5121*	37.10512	192.5418	444.534	2.007342541	0.880560582	2.775082265	0.003635076	0.156392554	0.000256703
miR-5126	*miR-5126*	12.46791	10.74857	6.082526	-0.261478224	-0.654504419	-1.055419228	0.900566621	0.637978879	0.317586175
miR-5128	*miR-5128*	4.607789	18.58744	13.75491	1.657146721	-0.474383907	1.052286568	0.059245804	0.55389649	0.118316853
miR-5621	*miR-5621-5p*	3.393188	26.97842	8.131531	1.776804752	-1.23645036	0.341302224	0.397269715	0.564379148	0.85478239
miR-6238	*miR-6238*	6.439843	68.59899	108.9833	2.872209478	-0.085780454	2.655930352	0.002875447	0.940022491	0.013255487
miR-6240	*miR-6240*	4120.893	3236.247	1528.887	-0.352840672	-1.209176477	-1.637448131	0.877236949	0.449030635	0.176684376
miR-6412	*miR-6412*	0.770444	4.151088	14.67231	2.531250418	1.112858085	3.439711994	0.061030138	0.452200482	0.022574041
miR-6538	*miR-6538*	26.17419	29.68917	11.64147	0.231428507	-1.071497142	-0.961845849	0.925309054	0.524496478	0.467775709
miR-6937	*miR-6937-5p*	17.28693	11.72446	28.12969	-1.112950805	1.582142177	0.207510628	0.516540828	0.399004793	0.875877556
miR-7033	*miR-7033-5p*	24.64218	389.7688	61.82609	3.446815876	-2.696377083	0.613102857	0.008185607	0.024798604	0.530653892
miR-7087	*miR-7087-3p*	0.040285	0.261091	12.3902	0	4.554384766	5.600440643	0	0.084301339	0.022574041

Table S3: Comparison of miRNAs identified in mouse epididymal spermatozoa, epithelial cells and epididymosomes

miRNA Family	Mature miRNA	Total			Spermatozoa[1]			Epithelial Cells[2]			Epididymosomes		
		Spermatozoa	Epithelial Cells	Epididymosomes	Caput	Corpus	Cauda	Caput	Corpus	Cauda	Caput	Corpus	Cauda
**miRNAs represented in mouse epididymal sperm, epithelial cell, and epididymosome datasets (all regions)**													
let-7	*let-7a-5p*	+	+	+	+	+	+	+	+	+	+	+	+
	let-7b-5p	+	+	+	+	+	+	+	+	+	+	+	+
	let-7c-5p	+	+	+	+	+	+	+	+	+	+	+	+
	let-7d-3p	+	+	+	+	+	+	+	+	+	+	+	+
	let-7d-5p	+	+	+	+	+	+	+	+	+	+	+	+
	let-7f-5p	+	+	+	+	+	+	+	+	+	+	+	+
	let-7g-5p	+	+	+	+	+	+	+	+	+	+	+	+
	let-7i-5p	+	+	+	+	+	+	+	+	+	+	+	+
miR-10	*miR-10a-5p*	+	+	+	+	+	+	+	+	+	+	+	+
	miR-10b-5p	+	+	+	+	+	+	+	+	+	+	+	+
miR-15	*miR-15a-5p*	+	+	+	+	+	+	+	+	+	+	+	+
	miR-15b-5p	+	+	+	+	+	+	+	+	+	+	+	+
miR-16	*miR-16-5p*	+	+	+	+	+	+	+	+	+	+	+	+
miR-17	*miR-17-5p*	+	+	+	+	+	+	+	+	+	+	+	+
miR-19	*miR-19b-3p*	+	+	+	+	+	+	+	+	+	+	+	+
miR-20	*miR-20a-5p*	+	+	+	+	+	+	+	+	+	+	+	+
miR-22	*miR-22-3p*	+	+	+	+	+	+	+	+	+	+	+	+
miR-23	*miR-23a-3p*	+	+	+	+	+	+	+	+	+	+	+	+
	miR-23b-3p	+	+	+	+	+	+	+	+	+	+	+	+
miR-25	*miR-25-3p*	+	+	+	+	+	+	+	+	+	+	+	+
miR-26	*miR-26a-5p*	+	+	+	+	+	+	+	+	+	+	+	+
	miR-26b-5p	+	+	+	+	+	+	+	+	+	+	+	+
miR-27	*miR-27b-3p*	+	+	+	+	+	+	+	+	+	+	+	+
miR-29	*miR-29a-3p*	+	+	+	+	+	+	+	+	+	+	+	+
	miR-29b-3p	+	+	+	+	+	+	+	+	+	+	+	+
miR-29	*miR-29c-3p*	+	+	+	+	+	+	+	+	+	+	+	+
miR-30	*miR-30a-3p*	+	+	+	+	+	+	+	+	+	+	+	+
	miR-30a-5p	+	+	+	+	+	+	+	+	+	+	+	+
	miR-30b-5p	+	+	+	+	+	+	+	+	+	+	+	+
	miR-30c-5p	+	+	+	+	+	+	+	+	+	+	+	+
	miR-30d-3p	+	+	+	+	+	+	+	+	+	+	+	+
	miR-30d-5p	+	+	+	+	+	+	+	+	+	+	+	+
	miR-30e-3p	+	+	+	+	+	+	+	+	+	+	+	+
	miR-30e-5p	+	+	+	+	+	+	+	+	+	+	+	+
miR-34	*miR-34b-5p*	+	+	+	+	+	+	+	+	+	+	+	+
	miR-34c-5p	+	+	+	+	+	+	+	+	+	+	+	+
miR-92	*miR-92a-3p*	+	+	+	+	+	+	+	+	+	+	+	+
miR-93	*miR-93-5p*	+	+	+	+	+	+	+	+	+	+	+	+
miR-98	*miR-98-5p*	+	+	+	+	+	+	+	+	+	+	+	+
miR-99	*miR-99a-5p*	+	+	+	+	+	+	+	+	+	+	+	+
	miR-99b-5p	+	+	+	+	+	+	+	+	+	+	+	+
miR-100	*miR-100-5p*	+	+	+	+	+	+	+	+	+	+	+	+
miR-101	*miR-101a-3p*	+	+	+	+	+	+	+	+	+	+	+	+
	miR-101b-3p	+	+	+	+	+	+	+	+	+	+	+	+
miR-103	*miR-103-3p*	+	+	+	+	+	+	+	+	+	+	+	+
miR-125	*miR-125a-5p*	+	+	+	+	+	+	+	+	+	+	+	+
	miR-125b-5p	+	+	+	+	+	+	+	+	+	+	+	+
miR-130	*miR-130a-3p*	+	+	+	+	+	+	+	+	+	+	+	+
miR-135	*miR-135a-5p*	+	+	+	+	+	+	+	+	+	+	+	+
miR-141	*miR-141-3p*	+	+	+	+	+	+	+	+	+	+	+	+
miR-143	*miR-143-3p*	+	+	+	+	+	+	+	+	+	+	+	+
miR-146	*miR-146a-5p*	+	+	+	+	+	+	+	+	+	+	+	+
	miR-146b-5p	+	+	+	+	+	+	+	+	+	+	+	+
miR-148	*miR-148a-3p*	+	+	+	+	+	+	+	+	+	+	+	+
	miR-148a-5p	+	+	+	+	+	+	+	+	+	+	+	+
	miR-148b-3p	+	+	+	+	+	+	+	+	+	+	+	+
miR-151	*miR-151-3p*	+	+	+	+	+	+	+	+	+	+	+	+
	miR-151-5p	+	+	+	+	+	+	+	+	+	+	+	+
miR-181	*miR-181a-5p*	+	+	+	+	+	+	+	+	+	+	+	+
	miR-181c-3p	+	+	+	+	+	+	+	+	+	+	+	+
	miR-181c-5p	+	+	+	+	+	+	+	+	+	+	+	+
	miR-181d-5p	+	+	+	+	+	+	+	+	+	+	+	+
miR-182	*miR-182-5p*	+	+	+	+	+	+	+	+	+	+	+	+
miR-183	*miR-183-5p*	+	+	+	+	+	+	+	+	+	+	+	+
miR-186	*miR-186-5p*	+	+	+	+	+	+	+	+	+	+	+	+
miR-191	*miR-191-5p*	+	+	+	+	+	+	+	+	+	+	+	+
miR-192	*miR-192-5p*	+	+	+	+	+	+	+	+	+	+	+	+
miR-200	*miR-200a-3p*	+	+	+	+	+	+	+	+	+	+	+	+
	miR-200a-5p	+	+	+	+	+	+	+	+	+	+	+	+
	miR-200b-3p	+	+	+	+	+	+	+	+	+	+	+	+
	miR-200c-3p	+	+	+	+	+	+	+	+	+	+	+	+
miR-204	*miR-204-5p*	+	+	+	+	+	+	+	+	+	+	+	+
miR-205	*miR-205-5p*	+	+	+	+	+	+	+	+	+	+	+	+
miR-210	*miR-210-3p*	+	+	+	+	+	+	+	+	+	+	+	+
miR-221	*miR-221-3p*	+	+	+	+	+	+	+	+	+	+	+	+
miR-222	*miR-222-3p*	+	+	+	+	+	+	+	+	+	+	+	+
miR-301	*miR-301a-3p*	+	+	+	+	+	+	+	+	+	+	+	+
miR-320	*miR-320-3p*	+	+	+	+	+	+	+	+	+	+	+	+
miR-324	*miR-324-5p*	+	+	+	+	+	+	+	+	+	+	+	+
miR-328	*miR-328-3p*	+	+	+	+	+	+	+	+	+	+	+	+
miR-340	*miR-340-5p*	+	+	+	+	+	+	+	+	+	+	+	+
miR-342	*miR-342-3p*	+	+	+	+	+	+	+	+	+	+	+	+
miR-361	*miR-361-5p*	+	+	+	+	+	+	+	+	+	+	+	+
miR-375	*miR-375-3p*	+	+	+	+	+	+	+	+	+	+	+	+
miR-410	*miR-410-3p*	+	+	+	+	+	+	+	+	+	+	+	+
miR-423	*miR-423-3p*	+	+	+	+	+	+	+	+	+	+	+	+
	miR-423-5p	+	+	+	+	+	+	+	+	+	+	+	+
miR-425	*miR-425-5p*	+	+	+	+	+	+	+	+	+	+	+	+
miR-429	*miR-429-3p*	+	+	+	+	+	+	+	+	+	+	+	+
miR-434	*miR-434-3p*	+	+	+	+	+	+	+	+	+	+	+	+
miR-467	*miR-467a-5p*	+	+	+	+	+	+	+	+	+	+	+	+
miR-470	*miR-470-5p*	+	+	+	+	+	+	+	+	+	+	+	+
miR-484	*miR-484*	+	+	+	+	+	+	+	+	+	+	+	+
miR-501	*miR-501-3p*	+	+	+	+	+	+	+	+	+	+	+	+
miR-532	*miR-532-5p*	+	+	+	+	+	+	+	+	+	+	+	+
miR-672	*miR-672-5p*	+	+	+	+	+	+	+	+	+	+	+	+
miR-744	*miR-744-5p*	+	+	+	+	+	+	+	+	+	+	+	+
miR-871	*miR-871-3p*	+	+	+	+	+	+	+	+	+	+	+	+
miR-872	*miR-872-5p*	+	+	+	+	+	+	+	+	+	+	+	+
miR-1839	*miR-1839-5p*	+	+	+	+	+	+	+	+	+	+	+	+
miRNA Family	Mature miRNA	Total			Spermatozoa[1]			Epithelial Cells[2]			Epididymosomes		
		Spermatozoa	Epithelial Cells	Epididymosomes	Caput	Corpus	Cauda	Caput	Corpus	Cauda	Caput	Corpus	Cauda
**miRNAs represented in mouse epididymal sperm, epithelial cell, and epididymosome datasets**													
let-7	*let-7a-1-3p*	+	+	+	+	-	+	+	-	+	+	+	+
	let-7b-3p	+	+	+	+	-	-	+	-	+	+	+	+
	let-7c-2-3p	+	+	+	+	-	+	+	-	+	+	+	+
	let-7e-5p	+	+	+	+	+	-	+	+	+	+	+	+
miR-9	*miR-9-5p*	+	+	+	+	+	+	+	-	-	+	+	+
miR-10	*miR-10a-3p*	+	+	+	+	-	-	+	-	-	+	+	+
miR-19	*miR-19a-3p*	+	+	+	+	+	+	+	-	+	+	+	+
miR-22	*miR-22-5p*	+	+	+	+	-	-	+	-	+	+	+	+
miR-24	*miR-24-2-5p*	+	+	+	+	+	-	+	+	+	+	+	+
	miR-24-3p	+	+	+	+	+	-	+	+	+	+	+	+
miR-27	*miR-27a-3p*	+	+	+	+	+	-	+	+	+	+	+	+
miR-29	*miR-29a-5p*	+	+	+	+	-	-	+	-	+	-	-	+
miR-30	*miR-30c-2-3p*	+	+	+	+	+	+	+	-	+	+	+	+
miR-31	*miR-31-5p*	+	+	+	+	+	+	+	-	+	+	+	+
miR-34	*miR-34a-5p*	+	+	+	+	+	-	+	+	+	+	+	+
miR-34	*miR-34b-3p*	+	+	+	+	+	+	+	-	+	+	+	+
miR-92	*miR-92b-3p*	+	+	+	+	+	-	+	-	+	+	+	+
miR-96	*miR-96-5p*	+	+	+	+	+	-	+	+	+	+	+	+
miR-99	*miR-99b-3p*	+	+	+	+	-	-	+	-	-	+	+	+
miR-106	*miR-106b-5p*	+	+	+	+	+	-	+	+	+	+	+	+
miR-107	*miR-107-3p*	+	+	+	+	-	+	+	-	+	+	+	+

miR-125	*miR-125b-1-3p*	+	+	+	+	+	-	+	-	-	+	+	+
	miR-125b-2-3p	+	+	+	+	-	-	+	-	+	+	+	+
miR-127	*miR-127-3p*	+	+	+	+	+	-	+	+	+	+	+	+
miR-128	*miR-128-3p*	+	+	+	+	+	+	+	-	+	+	+	+
miR-132	*miR-132-3p*	+	+	+	+	-	+	+	-	+	-	+	+
miR-133	*miR-133a-3p*	+	+	+	+	+	-	+	+	+	+	+	+
miR-138	*miR-138-5p*	+	+	+	+	+	+	+	-	-	+	+	+
miR-140	*miR-140-3p*	+	+	+	-	-	+	+	-	+	+	+	+
	miR-140-5p	+	+	+	+	-	+	+	+	+	-	-	+
miR-141	*miR-141-5p*	+	+	+	+	-	-	+	-	+	+	+	+
miR-144	*miR-144-3p*	+	+	+	+	+	-	-	-	+	-	-	+
miR-148	*miR-148b-5p*	+	+	+	+	-	+	+	-	-	+	+	+
miR-149	*miR-149-5p*	+	+	+	+	-	-	+	-	+	+	+	+
miR-150	*miR-150-5p*	+	+	+	+	+	-	+	+	+	+	+	+
miR-152	*miR-152-3p*	+	+	+	+	+	-	+	+	+	+	+	+
	miR-152-5p	+	+	+	+	-	-	+	-	+	+	+	+
miR-153	*miR-153-3p*	+	+	+	+	-	+	+	-	-	-	+	-
miR-181	*miR-181a-1-3p*	+	+	+	+	+	-	+	-	+	+	+	+
	miR-181b-5p	+	+	+	+	+	-	+	+	+	+	+	+
miR-184	*miR-184-3p*	+	+	+	+	+	+	-	+	+	-	+	+
miR-187	*miR-187-3p*	+	+	+	+	+	-	+	+	+	+	+	+
miR-194	*miR-194-5p*	+	+	+	+	-	+	+	-	+	+	+	+
miR-196	*miR-196a-5p*	+	+	+	+	+	-	+	+	+	-	+	+
	miR-196b-5p	+	+	+	+	+	-	+	+	+	-	+	+
miR-199	*miR-199a-3p*	+	+	+	+	+	-	+	+	+	+	+	+
	miR-199a-5p	+	+	+	+	+	-	+	+	+	+	+	+
	miR-199b-3p	+	+	+	+	+	-	+	+	+	+	+	+
	miR-199b-5p	+	+	+	+	+	-	+	-	+	+	+	+
miR-200	*miR-200b-5p*	+	+	+	+	-	-	+	-	+	+	+	+
miR-203	*miR-203-3p*	+	+	+	+	+	-	+	+	+	+	+	+
miR-214	*miR-214-3p*	+	+	+	+	+	-	+	+	+	+	+	+
miR-218	*miR-218-5p*	+	+	+	+	+	-	+	+	+	+	+	+
miR-221	*miR-221-5p*	+	+	+	+	-	-	+	-	-	+	-	+
miR-322	*miR-322-5p*	+	+	+	+	+	-	+	+	+	+	+	+
miR-326	*miR-326-3p*	+	+	+	+	-	-	+	-	+	-	+	+
miR-338	*miR-338-3p*	+	+	+	+	-	-	+	-	+	+	+	+
miR-339	*miR-339-5p*	+	+	+	+	-	-	+	-	+	+	+	+
miR-350	*miR-350-3p*	+	+	+	-	-	+	+	-	-	-	+	+
miR-351	*miR-351-5p*	+	+	+	+	+	-	+	+	+	+	+	+
miR-362	*miR-362-3p*	+	+	+	+	-	-	+	-	-	+	+	+
miR-365	*miR-365-3p*	+	+	+	+	+	-	-	-	+	+	+	+
miR-379	*miR-379-5p*	+	+	+	+	-	-	-	-	+	-	+	+
miR-381	*miR-381-3p*	+	+	+	+	-	-	-	-	+	+	+	+
miR-411	*miR-411-5p*	+	+	+	+	+	-	+	+	+	+	+	+
miR-421	*miR-421-3p*	+	+	+	+	+	-	+	+	+	+	+	+
miR-449	*miR-449a-5p*	+	+	+	+	+	+	+	-	+	+	+	+
miR-450	*miR-450a-5p*	+	+	+	+	-	-	+	-	+	+	+	+
miR-455	*miR-455-5p*	+	+	+	+	-	-	+	-	-	+	+	+
miR-465	*miR-465a-5p*	+	+	+	+	+	+	-	-	+	-	+	+
	miR-465c-5p	+	+	+	+	+	+	-	+	+	+	+	+
miR-467	*miR-467d-5p*	+	+	+	+	-	-	+	-	-	+	+	-
miR-541	*miR-541-5p*	+	+	+	+	-	-	-	-	+	+	+	+
miR-574	*miR-574-3p*	+	+	+	+	+	-	+	-	+	+	+	+
miR-582	*miR-582-3p*	+	+	+	+	-	-	+	-	-	+	+	+
miR-652	*miR-652-3p*	+	+	+	+	+	-	+	+	+	+	+	+
miR-669	*miR-669a-5p*	+	+	+	+	-	+	+	-	-	+	+	+
	miR-669c-5p	+	+	+	+	-	+	+	-	-	+	-	-
	miR-669p-5p	+	+	+	+	-	+	+	-	-	+	+	+
miR-671	*miR-671-3p*	+	+	+	+	-	-	+	-	-	+	-	-
miR-676	*miR-676-3p*	+	+	+	+	-	+	+	-	+	+	+	+
miR-741	*miR-741-3p*	+	+	+	+	+	+	-	+	+	+	+	+
miR-743	*miR-743b-3p*	+	+	+	+	+	+	-	+	+	+	+	+
miR-871	*miR-871-5p*	+	+	+	+	+	+	-	-	+	-	+	+
miR-872	*miR-872-3p*	+	+	+	+	-	+	+	+	+	+	+	+
miR-881	*miR-881-3p*	+	+	+	+	+	+	-	+	+	+	+	+
miR-1198	*miR-1198-5p*	+	+	+	+	+	-	+	-	+	+	+	+
miR-1249	*miR-1249-3p*	+	+	+	+	-	-	+	-	-	+	+	+
miR-1251	*miR-1251-5p*	+	+	+	+	-	-	+	-	-	+	+	+
miR-1843	*miR-1843b-5p*	+	+	+	+	-	-	+	-	-	+	+	+
		**Total**			**Spermatozoa[1]**			**Epithelial Cells[2]**			**Epididymosomes**		
**miRNA Family**	**Mature miRNA**	Spermatozoa	Epithelial Cells	Epididymosomes	Caput	Corpus	Cauda	Caput	Corpus	Cauda	Caput	Corpus	Cauda

miRNAs represented in mouse epididymal sperm and epithelial cell (but not epididymosome) datasets

miR-9	*miR-9-3p*	+	+	-	+	-	-	+	-	-	-	-	-
miR-21	*miR-21-3p*	+	+	-	+	+	-	+	-	+	-	-	-
	miR-21-5p	+	+	-	+	+	+	+	+	+	-	-	-
miR-28	*miR-28-3p*	+	+	-	+	-	+	+	+	+	-	-	-
	miR-28-5p	+	+	-	+	+	+	+	+	+	-	-	-
miR-126	*miR-126-3p*	+	+	-	+	+	-	+	+	+	-	-	-
	miR-126-5p	+	+	-	+	+	+	+	+	+	-	-	-
miR-142	*miR-142-5p*	+	+	-	+	+	+	+	+	+	-	-	-
miR-145	*miR-145-3p*	+	+	-	+	-	-	+	+	+	-	-	-
	miR-145-5p	+	+	-	+	+	-	+	+	+	-	-	-
miR-190	*miR-190-5p*	+	+	-	-	+	-	-	+	+	-	-	-
miR-193	*miR-193-3p*	+	+	-	+	-	-	+	+	+	-	-	-
miR-195	*miR-195-5p*	+	+	-	+	+	+	+	+	+	-	-	-
miR-374	*miR-374-5p*	+	+	-	+	+	-	+	+	+	-	-	-
miR-378	*miR-378-3p*	+	+	-	+	+	+	+	+	+	-	-	-
	miR-378-5p	+	+	-	+	-	-	+	-	-	-	-	-
miR-451	*miR-451*	+	+	-	+	+	-	+	-	+	-	-	-
miR-486	*miR-486-5p*	+	+	-	+	+	+	+	+	+	-	-	-
miR-497	*miR-497-5p*	+	+	-	+	+	+	+	+	+	-	-	-
miR-720	*miR-720*	+	+	-	+	-	+	+	-	-	-	-	-
miR-1843	*miR-1843-5p*	+	+	-	+	+	+	+	+	+	-	-	-
miR-3096	*miR-3096-5p*	+	+	-	+	-	-	+	-	+	-	-	-
miR-3107	*miR-3107-5p*	+	+	-	+	+	+	+	+	+	-	-	-
		**Total**			**Spermatozoa[1]**			**Epithelial Cells[2]**			**Epididymosomes**		
**miRNA Family**	**Mature miRNA**	Spermatozoa	Epithelial Cells	Epididymosomes	Caput	Corpus	Cauda	Caput	Corpus	Cauda	Caput	Corpus	Cauda

miRNAs represented in mouse epididymal sperm and epididymosome (but not epithelial cell) datasets

miR-1	*miR-1a-3p*	+	-	+	+	-	-	-	-	-	-	-	+
miR-7	*miR-7a-1-3p*	+	-	+	+	-	+	-	-	-	-	+	-
miR-18	*miR-18a-5p*	+	-	+	+	+	+	-	-	-	+	+	+
miR-27	*miR-27b-5p*	+	-	+	+	-	-	-	-	-	-	+	+
miR-34	*miR-34c-3p*	+	-	+	+	+	+	-	-	-	-	+	+
miR-106	*miR-106b-3p*	+	-	+	+	-	+	-	-	-	+	+	+
miR-130	*miR-130b-3p*	+	-	+	-	-	+	-	-	-	-	-	+
miR-135	*miR-135a-2-3p*	+	-	+	+	-	-	-	-	-	+	-	-
	miR-135b-5p	+	-	+	+	-	-	-	-	-	-	+	-
miR-185	*miR-185-5p*	+	-	+	+	-	-	-	-	-	-	+	+
miR-193	*miR-193b-3p*	+	-	+	-	-	+	-	-	-	-	+	+
miR-210	*miR-210-5p*	+	-	+	+	-	-	-	-	-	+	+	-
miR-214	*miR-214-5p*	+	-	+	+	-	-	-	-	-	+	+	+
miR-223	*miR-223-3p*	+	-	+	+	-	-	-	-	-	+	+	+
miR-296	*miR-296-5p*	+	-	+	+	-	+	-	-	-	+	-	-
miR-331	*miR-331-3p*	+	-	+	-	-	+	-	-	-	+	+	+
miR-335	*miR-335-3p*	+	-	+	+	-	-	-	-	-	+	+	+
miR-339	*miR-339-3p*	+	-	+	+	-	-	-	-	-	+	+	+
miR-340	*miR-340-3p*	+	-	+	+	-	+	-	-	-	+	+	+
miR-463	*miR-463-5p*	+	-	+	-	-	+	-	-	-	-	+	+
miR-465	*miR-465a-3p*	+	-	+	-	-	+	-	-	-	-	+	+
	miR-465b-3p	+	-	+	-	-	+	-	-	-	-	+	+
	miR-465b-5p	+	-	+	-	-	+	-	-	-	-	-	+
	miR-465c-3p	+	-	+	-	-	+	-	-	-	-	+	+
miR-466	*miR-466a-3p*	+	-	+	-	-	+	-	-	-	+	-	-
	miR-466b-3p	+	-	+	+	-	-	-	-	-	+	-	-
	miR-466c-3p	+	-	+	+	-	-	-	-	-	+	-	-
	miR-466e-3p	+	-	+	-	-	+	-	-	-	+	-	-
	miR-466p-3p	+	-	+	+	-	-	-	-	-	+	-	-
miR-467	*miR-467b-5p*	+	-	+	-	-	+	-	-	-	+	+	+

	miR-467c-5p	+	-	+	+	-	+	-	-	-	+	-	-
	miR-467d-3p	+	-	+	+	-	+	-	-	-	+	-	-
	miR-467e-5p	+	-	+	+	-	+	-	-	-	+	+	-
miR-470	miR-470-3p	+	-	+	-	-	+	-	-	-	-	-	+
miR-471	miR-471-5p	+	-	+	-	-	+	-	-	-	-	-	+
miR-500	miR-500-3p	+	-	+	+	-	-	-	-	-	+	+	+
miR-511	miR-511-3p	+	-	+	+	-	-	-	-	-	-	+	+
miR-532	miR-532-3p	+	-	+	+	-	-	-	-	-	-	-	+
miR-574	miR-574-5p	+	-	+	+	-	-	-	-	-	+	+	+
miR-598	miR-598-3p	+	-	+	+	-	-	-	-	-	-	+	-
miR-615	miR-615-3p	+	-	+	+	-	-	-	-	-	+	+	+
miR-669	miR-669a-3p	+	-	+	+	-	+	-	-	-	+	+	-
	miR-669o-3p	+	-	+	+	-	+	-	-	-	+	+	-
miR-674	miR-674-3p	+	-	+	+	-	-	-	-	-	+	-	+
miR-676	miR-676-5p	+	-	+	-	-	+	-	-	-	-	+	+
miR-743	miR-743a-3p	+	-	+	+	-	+	-	-	-	-	+	+
miR-874	miR-874-3p	+	-	+	+	-	-	-	-	-	+	+	+
miR-878	miR-878-5p	+	-	+	+	-	+	-	-	-	-	+	+
miR-880	miR-880-3p	+	-	+	-	-	+	-	-	-	-	-	+
miR-883	miR-883a-3p	+	-	+	-	-	+	-	-	-	-	+	+
miR-3068	miR-3068-3p	+	-	+	+	-	-	-	-	-	+	+	+
miR-3473	miR-3473b	+	-	+	+	+	-	-	-	-	+	+	+

miRNA Family	Mature miRNA	Total			Spermatozoa[1]			Epithelial Cells[2]			Epididymosomes		
		Spermatozoa	Epithelial Cells	Epididymosomes	Caput	Corpus	Cauda	Caput	Corpus	Cauda	Caput	Corpus	Cauda

miRNAs represented in mouse epididymal epithalial cell and epididymosome (but not sperm) datasets

miR-139	miR-139-5p	-	+	+	-	-	-	-	-	+	-	+	+
miR-300	miR-300-3p	-	+	+	-	-	-	-	-	+	+	+	+
miR-409	miR-409-5p	-	+	+	-	-	-	-	-	+	-	-	+
miR-582	miR-582-5p	-	+	+	-	-	-	+	-	-	+	-	+

miRNA Family	Mature miRNA	Total			Spermatozoa[1]			Epithelial Cells[2]			Epididymosomes		
		Spermatozoa	Epithelial Cells	Epididymosomes	Caput	Corpus	Cauda	Caput	Corpus	Cauda	Caput	Corpus	Cauda

miRNAs represented only in mouse epididymal sperm datasets

let-7	let-7e-3p	+	-	-	+	-	-	-	-	-	-	-	-
	let-7f-1-3p	+	-	-	+	-	-	-	-	-	-	-	-
miR-16	miR-16-2-3p	+	-	-	-	-	+	-	-	-	-	-	-
miR-17	miR-17-3p	+	-	-	-	-	+	-	-	-	-	-	-
miR-27	miR-27a-5p	+	-	-	+	-	-	-	-	-	-	-	-
miR-92	miR-92a-1-5p	+	-	-	+	-	-	-	-	-	-	-	-
miR-124	miR-124-3p	+	-	-	-	-	+	-	-	-	-	-	-
miR-130	miR-130b-5p	+	-	-	-	-	+	-	-	-	-	-	-
miR-142	miR-142-3p	+	-	-	+	+	+	-	-	-	-	-	-
miR-144	miR-144-5p	+	-	-	+	-	-	-	-	-	-	-	-
miR-295	miR-295-3p	+	-	-	-	-	+	-	-	-	-	-	-
miR-301	miR-301b-3p	+	-	-	-	-	+	-	-	-	-	-	-
miR-378	miR-378b	+	-	-	+	-	-	-	-	-	-	-	-
miR-471	miR-471-3p	+	-	-	-	-	+	-	-	-	-	-	-
miR-499	miR-499-5p	+	-	-	-	-	+	-	-	-	-	-	-
miR-669	miR-669b-5p	+	-	-	+	-	-	-	-	-	-	-	-
	miR-669d-5p	+	-	-	+	-	+	-	-	-	-	-	-
	miR-669f-5p	+	-	-	+	-	-	-	-	-	-	-	-
	miR-669l-5p	+	-	-	+	-	-	-	-	-	-	-	-
	miR-669o-5p	+	-	-	+	-	+	-	-	-	-	-	-
miR-741	miR-741-5p	+	-	-	-	-	+	-	-	-	-	-	-
miR-743	miR-743a-5p	+	-	-	-	-	+	-	-	-	-	-	-
	miR-743b-5p	+	-	-	-	-	+	-	-	-	-	-	-
miR-744	miR-744-3p	+	-	-	+	-	-	-	-	-	-	-	-
miR-878	miR-878-3p	+	-	-	-	-	+	-	-	-	-	-	-
miR-883	miR-883b-3p	+	-	-	-	-	+	-	-	-	-	-	-
miR-1843	miR-1843-3p	+	-	-	+	-	-	-	-	-	-	-	-
miR-3068	miR-3068-5p	+	-	-	+	-	-	-	-	-	-	-	-
miR-3096	miR-3096b-3p	+	-	-	+	-	-	-	-	-	-	-	-
miR-3471	miR-3471	+	-	-	-	-	+	-	-	-	-	-	-

miRNA Family	Mature miRNA	Total			Spermatozoa[1]			Epithelial Cells[2]			Epididymosomes		
		Spermatozoa	Epithelial Cells	Epididymosomes	Caput	Corpus	Cauda	Caput	Corpus	Cauda	Caput	Corpus	Cauda

miRNAs represented only in mouse epididymal epididymosome datasets

let-7	let-7f-2-3p	-	-	+	-	-	-	-	-	-	+	+	-
	let-7i-3p	-	-	+	-	-	-	-	-	-	+	+	+
	let-7j	-	-	+	-	-	-	-	-	-	+	+	+
miR-10	miR-10b-3p	-	-	+	-	-	-	-	-	-	+	+	+
miR-15	miR-15b-3p	-	-	+	-	-	-	-	-	-	-	-	+
miR-21	miR-21a-3p	-	-	+	-	-	-	-	-	-	+	+	+
	miR-21a-5p	-	-	+	-	-	-	-	-	-	+	+	+
miR-28	miR-28a-3p	-	-	+	-	-	-	-	-	-	+	+	+
	miR-28a-5p	-	-	+	-	-	-	-	-	-	+	+	+
miR-31	miR-31-3p	-	-	+	-	-	-	-	-	-	-	-	+
miR-32	miR-32-5p	-	-	+	-	-	-	-	-	-	+	+	+
miR-99	miR-99a-3p	-	-	+	-	-	-	-	-	-	+	+	+
miR-101	miR-101a-5p	-	-	+	-	-	-	-	-	-	-	+	+
	miR-101c	-	-	+	-	-	-	-	-	-	+	+	+
miR-126	miR-126a-3p	-	-	+	-	-	-	-	-	-	+	+	+
	miR-126a-5p	-	-	+	-	-	-	-	-	-	+	+	+
miR-127	miR-127-5p	-	-	+	-	-	-	-	-	-	-	+	+
miR-133	miR-133a-5p	-	-	+	-	-	-	-	-	-	-	-	+
	miR-133b-3p	-	-	+	-	-	-	-	-	-	-	-	+
miR-134	miR-134-5p	-	-	+	-	-	-	-	-	-	-	+	+
miR-136	miR-136-3p	-	-	+	-	-	-	-	-	-	+	+	+
	miR-136-5p	-	-	+	-	-	-	-	-	-	+	+	+
miR-137	miR-137-3p	-	-	+	-	-	-	-	-	-	-	-	+
miR-142	miR-142a-3p	-	-	+	-	-	-	-	-	-	+	+	+
	miR-142a-5p	-	-	+	-	-	-	-	-	-	+	+	+
miR-143	miR-143-5p	-	-	+	-	-	-	-	-	-	+	-	+
miR-145	miR-145a-3p	-	-	+	-	-	-	-	-	-	+	+	+
	miR-145a-5p	-	-	+	-	-	-	-	-	-	+	+	+
	miR-145b	-	-	+	-	-	-	-	-	-	-	-	+
miR-155	miR-155-5p	-	-	+	-	-	-	-	-	-	-	-	+
miR-183	miR-183-3p	-	-	+	-	-	-	-	-	-	+	+	-
miR-190	miR-190a-5p	-	-	+	-	-	-	-	-	-	+	+	+
miR-193	miR-193a-3p	-	-	+	-	-	-	-	-	-	-	-	+
miR-195	miR-195a-5p	-	-	+	-	-	-	-	-	-	+	+	+
miR-208	miR-208b-3p	-	-	+	-	-	-	-	-	-	-	-	+
miR-211	miR-211-5p	-	-	+	-	-	-	-	-	-	+	+	-
miR-298	miR-298-5p	-	-	+	-	-	-	-	-	-	+	-	-
miR-299	miR-299a-3p	-	-	+	-	-	-	-	-	-	-	-	+
	miR-299a-5p	-	-	+	-	-	-	-	-	-	-	-	+
miR-322	miR-322-3p	-	-	+	-	-	-	-	-	-	+	+	+
miR-324	miR-324-3p	-	-	+	-	-	-	-	-	-	-	+	-
miR-329	miR-329-3p	-	-	+	-	-	-	-	-	-	-	-	+
	miR-329-5p	-	-	+	-	-	-	-	-	-	-	-	+
miR-330	miR-330-5p	-	-	+	-	-	-	-	-	-	+	+	+
miR-335	miR-335-5p	-	-	+	-	-	-	-	-	-	+	+	+
miR-337	miR-337-3p	-	-	+	-	-	-	-	-	-	-	-	+
	miR-337-5p	-	-	+	-	-	-	-	-	-	-	-	+
miR-338	miR-338-5p	-	-	+	-	-	-	-	-	-	-	-	+
miR-341	miR-341-3p	-	-	+	-	-	-	-	-	-	+	+	+
miR-345	miR-345-3p	-	-	+	-	-	-	-	-	-	+	+	+
	miR-345-5p	-	-	+	-	-	-	-	-	-	+	+	+
miR-361	miR-361-3p	-	-	+	-	-	-	-	-	-	+	+	+
miR-369	miR-369-5p	-	-	+	-	-	-	-	-	-	-	-	+
miR-374	miR-374b-5p	-	-	+	-	-	-	-	-	-	+	+	+
miR-376	miR-376b-3p	-	-	+	-	-	-	-	-	-	-	-	+
miR-378	miR-378a-3p	-	-	+	-	-	-	-	-	-	+	+	+
	miR-378a-5p	-	-	+	-	-	-	-	-	-	-	-	+
	miR-378c	-	-	+	-	-	-	-	-	-	+	+	+
	miR-378d	-	-	+	-	-	-	-	-	-	-	+	+
miR-409	miR-409-3p	-	-	+	-	-	-	-	-	-	+	+	+
miR-425	miR-425-3p	-	-	+	-	-	-	-	-	-	+	+	-
miR-431	miR-431-5p	-	-	+	-	-	-	-	-	-	-	-	+

miR-434	*miR-434-5p*	-	-	+	-	-	-	-	-	-	+	+	+
miR-450	*miR-450b-5p*	-	-	+	-	-	-	-	-	-	+	-	-
miR-451	*miR-451a*	-	-	+	-	-	-	-	-	-	+	+	+
miR-455	*miR-455-3p*	-	-	+	-	-	-	-	-	-	+	+	+
miR-466	*miR-466i-5p*	-	-	+	-	-	-	-	-	-	-	+	+
miR-467	*miR-467a-3p*	-	-	+	-	-	-	-	-	-	+	-	-
miR-486	*miR-486a-3p*	-	-	+	-	-	-	-	-	-	-	-	+
	miR-486a-5p	-	-	+	-	-	-	-	-	-	+	+	+
	miR-486b-5p	-	-	+	-	-	-	-	-	-	+	+	+
miR-496	*miR-496a-3p*	-	-	+	-	-	-	-	-	-	-	-	+
miR-497	*miR-497a-5p*	-	-	+	-	-	-	-	-	-	+	+	+
miR-542	*miR-542-3p*	-	-	+	-	-	-	-	-	-	-	+	-
miR-664	*miR-664-3p*	-	-	+	-	-	-	-	-	-	+	+	+
miR-677	*miR-677-3p*	-	-	+	-	-	-	-	-	-	-	+	-
	miR-677-5p	-	-	+	-	-	-	-	-	-	+	+	+
miR-690	*miR-690*	-	-	+	-	-	-	-	-	-	+	+	+
miR-708	*miR-708-3p*	-	-	+	-	-	-	-	-	-	-	+	+
	miR-708-5p	-	-	+	-	-	-	-	-	-	-	+	+
miR-709	*miR-709*	-	-	+	-	-	-	-	-	-	-	+	+
miR-714	*miR-714*	-	-	+	-	-	-	-	-	-	+	+	-
miR-879	*miR-879-5p*	-	-	+	-	-	-	-	-	-	+	-	-
miR-1195	*miR-1195*	-	-	+	-	-	-	-	-	-	+	+	+
miR-1247	*miR-1247-5p*	-	-	+	-	-	-	-	-	-	+	+	+
miR-1291	*miR-1291*	-	-	+	-	-	-	-	-	-	-	-	+
miR-1839	*miR-1839-3p*	-	-	+	-	-	-	-	-	-	+	+	+
miR-1843	*miR-1843a-5p*	-	-	+	-	-	-	-	-	-	+	+	+
	miR-1843b-3p	-	-	+	-	-	-	-	-	-	+	-	-
miR-1957	*miR-1957a*	-	-	+	-	-	-	-	-	-	+	+	+
miR-1981	*miR-1981-3p*	-	-	+	-	-	-	-	-	-	-	+	-
	miR-1981-5p	-	-	+	-	-	-	-	-	-	-	+	+
miR-3470	*miR-3470a*	-	-	+	-	-	-	-	-	-	+	+	+
	miR-3470b	-	-	+	-	-	-	-	-	-	+	+	+
miR-3473	*miR-3473a*	-	-	+	-	-	-	-	-	-	+	+	+
	miR-3473e	-	-	+	-	-	-	-	-	-	+	+	+
	miR-3473f	-	-	+	-	-	-	-	-	-	-	+	+
miR-3535	*miR-3535*	-	-	+	-	-	-	-	-	-	+	+	+
miR-5099	*miR-5099*	-	-	+	-	-	-	-	-	-	+	+	+
miR-5100	*miR-5100*	-	-	+	-	-	-	-	-	-	+	-	+
miR-5119	*miR-5119*	-	-	+	-	-	-	-	-	-	-	-	+
miR-5121	*miR-5121*	-	-	+	-	-	-	-	-	-	+	+	+
miR-5126	*miR-5126*	-	-	+	-	-	-	-	-	-	+	+	-
miR-5128	*miR-5128*	-	-	+	-	-	-	-	-	-	-	+	+
miR-5621	*miR-5621-5p*	-	-	+	-	-	-	-	-	-	-	+	-
miR-6238	*miR-6238*	-	-	+	-	-	-	-	-	-	-	+	+
miR-6240	*miR-6240*	-	-	+	-	-	-	-	-	-	+	+	+
miR-6412	*miR-6412*	-	-	+	-	-	-	-	-	-	-	-	+
miR-6538	*miR-6538*	-	-	+	-	-	-	-	-	-	+	+	+
miR-6937	*miR-6937-5p*	-	-	+	-	-	-	-	-	-	+	+	+
miR-7033	*miR-7033-5p*	-	-	+	-	-	-	-	-	-	+	+	+
miR-7087	*miR-7087-3p*	-	-	+	-	-	-	-	-	-	-	-	+

[1] Nixon, B., Stanger, S. J., Mihalas, B. P., Reilly, J. N., Anderson, A. L., Dun, M. D., Tyagi, S., Holt, J. E., and McLaughlin, E. A. (2015) Next Generation Sequencing Analysis Reveals Segmental Patterns of microRNA Expression in Mouse Epididymal Epithelial Cells. PLoS ONE 10, e0135605

[2] Nixon, B., Stanger, S. J., Mihalas, B. P., Reilly, J. N., Anderson, A. L., Tyagi, S., Holt, J. E., and McLaughlin, E. A. (2015) The MicroRNA Signature of Mouse Spermatozoa Is Substantially Modified During Epididymal Maturation. Biol Reprod 93, 91

Table S4: Comparison of mouse epididymal miRNA profile reported by Nixon et al., 2015a,b and Reily et al., 2016 versus Sharma et al., 2016

miRNA (mmu)	Epithelial Cells				Spermatozoa				Epididymosomes	
	Caput		Cauda		Caput		Cauda		Cauda	
	Nixon[1]	Sharma[2]	Nixon[1]	Sharma[2]	Nixon[3]	Sharma[2]	Nixon[3]	Sharma[2]	Reilly	Sharma[2]
let-7a	+	-	+	-	+	-	+	-	+	-
let-7a-1	+	+	+	+	+	+	+	+	+	+
let-7b	+	+	+	+	+	+	+	+	+	+
let-7c	+	-	+	-	+	-	+	-	+	-
let-7c-2	+	+	+	+	+	+	+	+	+	+
let-7d	+	+	+	+	+	+	+	+	+	+
let-7e	+	+	+	+	+	+	-	-	+	+
let-7f	+	-	+	-	+	-	+	-	+	-
let-7f-2	-	-	-	-	-	-	-	-	+	+
let-7f-1	-	-	-	-	+	+	-	-	-	-
let-7g	+	+	+	+	+	+	+	+	+	+
let-7j	-	-	-	-	-	-	-	-	+	+
let-7i	+	+	+	+	+	+	+	+	+	+
miR-1a	-	-	-	-	+	-	-	-	+	-
miR-7a-1	-	-	-	-	+	-	+	-	+	-
miR-9	+	-	-	-	+	-	+	-	+	-
miR-10a	+	+	+	+	+	+	+	+	+	+
miR-10b	+	+	+	+	+	+	+	+	+	+
miR-15a	+	+	+	+	+	+	+	+	+	+
miR-15b	+	+	+	+	+	+	+	+	+	+
miR-16	+	-	+	-	+	-	+	-	+	-
miR-16-2	-	-	-	-	-	-	+	+	-	-
miR-17	+	+	+	+	+	+	+	+	+	+
miR-18a	-	-	-	-	+	-	+	-	+	-
miR-19a	+	+	+	+	+	+	+	+	+	+
miR-19b	+	-	+	-	+	-	+	-	+	-
miR-20a	+	+	+	+	+	+	+	+	+	+
miR-21	+	-	+	-	+	-	+	-	-	-
miR-21a	-	-	-	-	-	-	-	-	+	+
miR-22	+	+	+	+	+	+	+	+	+	+
miR-23a	+	+	+	+	+	+	+	+	+	+
miR-23b	+	+	+	+	+	+	+	+	+	+
miR-24	+	-	+	-	+	-	-	-	+	-
miR-24-2	+	+	+	+	+	+	-	-	+	+
miR-25	+	+	+	+	+	+	+	+	+	+
miR-26a	+	-	+	-	+	-	+	-	+	-
miR-26b	+	+	+	+	+	+	+	+	+	+
miR-27a	+	+	+	+	+	+	-	-	+	+
miR-27b	+	+	+	+	+	+	+	+	+	+
miR-28	+	-	+	-	+	-	+	-	-	-
miR-28a	-	-	-	-	-	-	-	-	+	-
miR-29a	+	+	+	+	+	+	+	+	+	+
miR-29b	+	-	+	-	+	-	+	-	+	-
miR-29c	+	+	+	+	+	+	+	+	+	+
miR-30a	+	+	+	+	+	+	+	+	+	+
miR-30b	+	+	+	+	+	+	+	+	+	+
miR-30c	+	-	+	-	+	-	+	-	+	-
miR-30c-2	+	+	+	+	+	+	+	+	+	+
miR-30d	+	+	+	+	+	+	+	+	+	+
miR-30e	+	+	+	+	+	+	+	+	+	+
miR-31	+	+	+	+	+	+	+	+	+	+
miR-32	-	-	-	-	-	-	-	-	+	+
miR-34a	+	+	+	+	+	+	-	-	+	+
miR-34b	+	+	+	+	+	+	+	+	+	+
miR-34c	+	+	+	+	+	+	+	+	+	+
miR-92a	+	-	+	-	+	-	+	-	+	-
miR-92a-1	-	-	-	-	+	-	-	-	-	-
miR-92b	+	+	+	-	+	-	-	-	+	-
miR-93	+	+	+	+	+	+	+	+	+	+
miR-96	+	+	+	+	+	+	-	-	+	+
miR-98	+	+	+	+	+	+	+	+	+	+
miR-99a	+	+	+	+	+	+	+	+	+	+
miR-99b	+	+	+	+	+	+	+	-	+	+
miR-100	+	+	+	+	+	+	+	+	+	+
miR-101a	+	+	+	+	+	+	+	+	+	+
miR-101b	+	+	+	+	+	+	+	+	+	+
miR-101c	-	-	-	-	-	-	-	-	+	+
miR-103	+	-	+	-	+	-	+	-	+	-
miR-106b	+	+	+	+	+	+	+	+	+	+
miR-107	+	+	+	+	+	+	+	+	+	+
miR-124	-	-	-	-	-	-	+	-	-	-
miR-125a	+	+	+	+	+	+	+	+	+	+
miR-125b	+	-	+	-	+	-	+	-	+	-
miR-125b-1	+	+	-	-	+	+	-	-	+	+
miR-125b-2	+	+	+	+	+	+	-	-	+	+
miR-126	+	-	+	-	+	-	+	-	-	-
miR-126a	-	-	-	-	-	-	-	-	+	+
miR-127	+	+	+	+	+	+	-	-	+	+
miR-128	+	-	+	-	+	-	+	-	+	-
miR-130a	+	+	+	+	+	+	+	+	+	+
miR-130b	-	-	-	-	-	-	+	+	+	+
miR-132	+	+	+	+	+	-	+	+	+	+
miR-133a	+	-	+	-	+	-	-	-	+	-
miR-133b	-	-	-	-	-	-	-	-	+	+
miR-134	-	-	-	-	-	-	-	-	+	+
miR-135a	+	-	+	-	+	-	+	-	+	-
miR-135a-2	-	-	-	-	+	+	-	-	+	+
miR-135b	-	-	-	-	+	+	-	-	+	+
miR-136	-	-	-	-	-	-	-	-	+	+
miR-137	-	-	-	-	-	-	-	-	+	+
miR-138	+	-	-	-	+	-	+	-	+	-
miR-139	-	-	+	+	-	-	-	-	+	+
miR-140	+	+	+	+	+	+	+	+	+	+
miR-141	+	+	+	+	+	+	+	+	+	+
miR-142	+	+	+	+	+	+	+	+	-	-
miR-142a	-	-	-	-	-	-	-	-	+	-
miR-143	+	+	+	+	+	+	+	+	+	+
miR-144	-	-	+	+	+	+	-	-	+	+
miR-145	+	-	+	-	+	-	-	-	-	-
miR-145a	-	-	-	-	-	-	-	-	+	+
miR-145b	-	-	-	-	-	-	-	-	+	+
miR-146a	+	-	+	-	+	-	+	-	+	-
miR-146b	+	+	+	+	+	+	+	+	+	+
miR-148a	+	+	+	+	+	+	+	-	+	+
miR-148b	+	+	+	+	+	-	+	+	+	+
miR-149	+	+	+	+	+	+	-	-	+	+
miR-150	+	+	+	+	+	+	-	-	+	+
miR-151	+	-	+	-	+	-	+	-	+	-
miR-152	+	+	+	+	+	+	-	-	+	+
miR-153	+	+	-	-	+	-	+	-	+	-
miR-155	-	-	-	-	-	-	-	-	+	+
miR-181a	+	-	+	-	+	-	+	-	+	-
miR-181a-1	+	+	+	+	+	+	-	-	+	+
miR-181b	+	-	+	-	+	-	-	-	+	-
miR-181c	+	+	+	+	+	+	+	+	+	+
miR-181d	+	+	+	+	+	+	+	+	+	+
miR-182	+	+	+	+	+	+	+	+	+	+
miR-183	+	+	+	+	+	+	+	+	+	+
miR-184	-	-	+	+	+	-	+	+	+	+
miR-185	-	-	-	-	+	+	-	-	+	+
miR-186	+	+	+	+	+	+	+	+	+	+
miR-187	+	+	+	+	+	+	-	-	+	+
miR-190	-	-	+	-	-	-	-	-	-	-
miR-190a	-	-	-	-	-	-	-	-	+	+
miR-191	+	+	+	+	+	+	+	+	+	+
miR-192	+	+	+	+	+	+	+	+	+	+
miR-193	+	-	+	-	+	-	-	-	-	-
miR-193a	-	-	-	-	-	-	-	-	+	+
miR-193b	-	-	-	-	-	-	+	+	+	+
miR-194	+	-	+	-	+	-	+	-	+	-
miR-195	+	-	+	-	+	-	+	-	-	-
miR-195a	-	-	-	-	-	-	-	-	+	+
miR-196a	+	-	+	-	+	-	-	-	+	-
miR-196b	+	+	+	+	+	+	-	-	+	+
miR-199a	+	-	+	-	+	-	-	-	+	-
miR-199b	+	+	+	+	+	+	-	-	+	+
miR-200a	+	+	+	+	+	+	+	+	+	+
miR-200b	+	+	+	+	+	+	+	+	+	+
miR-200c	+	+	+	+	+	+	+	+	+	+
miR-203	+	+	+	+	+	+	-	-	+	+
miR-204	+	+	+	+	+	+	+	+	+	+
miR-205	+	+	+	+	+	+	+	+	+	+
miR-208b	-	-	-	-	-	-	-	-	+	-
miR-210	+	+	+	+	+	+	+	+	+	+
miR-211	-	-	-	-	-	-	-	-	+	-
miR-214	+	+	+	+	+	+	-	-	+	+
miR-218	+	-	+	-	+	-	-	-	+	-
miR-221	+	+	+	+	+	+	+	+	+	+
miR-222	+	+	+	+	+	+	+	+	+	+
miR-223	-	-	-	-	+	+	-	-	+	+
miR-295	-	-	-	-	-	-	+	-	-	-
miR-296	-	-	-	-	+	+	+	+	+	+
miR-298	-	-	-	-	-	-	-	-	+	-
miR-299a	-	-	-	-	-	-	-	-	+	+
miR-300	-	-	+	+	-	-	-	-	+	-
miR-301a	+	-	+	-	+	-	+	-	+	-

Conservation[4]	Epithelial Cells		Spermatozoa		Epididymosomes
	Caput	Cauda	Caput	Cauda	Cauda
**Number**	121/170	120/168	121/213	97/156	184/279
**Percentage**	71.18	71.43	56.81	62.18	65.95

[4] *Conservation presented is calculated as the percentage of miRNAs identified in Sharma et al 2016 that were also identified in the studies of Nixon and colleagues eg. of the 170 miRNAs identified in caput epididymal epithelial cells in Nixon et al 2015 (a), 121 were identified in Sharma et al 2016 (71.18%)*

miR-301b	-	-	-	-	-	-	+	-	-	-
miR-320	+	+	+	+	+	+	+	+	+	+
miR-322	+	+	+	+	+	+	-	-	+	+
miR-324	+	+	+	+	+	+	+	-	+	+
miR-326	+	+	+	+	+	-	-	-	+	+
miR-328	+	+	+	+	+	+	+	+	+	+
miR-329	-	-	-	-	-	-	-	-	+	+
miR-330	-	-	-	-	-	-	-	-	+	+
miR-331	-	-	-	-	-	-	+	-	+	+
miR-335	-	-	-	-	+	-	-	-	+	+
miR-337	-	-	-	-	-	-	-	-	+	+
miR-338	+	+	+	+	+	-	-	-	+	+
miR-339	+	+	+	+	+	+	-	-	+	+
miR-340	+	+	+	+	+	+	+	+	+	+
miR-341	-	-	-	-	-	-	-	-	+	-
miR-342	+	-	+	-	+	-	+	-	+	-
miR-345	-	-	-	-	-	-	-	-	+	+
miR-350	+	+	-	-	-	-	+	+	+	+
miR-351	+	+	+	+	+	+	-	-	+	+
miR-361	+	-	+	-	+	-	+	-	+	-
miR-362	+	+	-	-	+	+	-	-	+	+
miR-365	-	-	+	-	+	-	-	-	+	-
miR-369	-	-	-	-	-	-	-	-	+	+
miR-374	+	-	+	-	+	-	-	-	-	-
miR-374b	-	-	-	-	-	-	-	-	+	+
miR-375	+	+	+	+	+	+	+	+	+	+
miR-376b	-	-	-	-	-	-	-	-	+	+
miR-378	+	-	+	-	+	-	+	-	-	-
miR-378a	-	-	-	-	-	-	-	-	+	-
miR-378b	-	-	-	-	+	+	-	-	-	-
miR-378c	-	-	-	-	-	-	-	-	+	-
miR-378d	-	-	-	-	-	-	-	-	+	-
miR-379	-	-	+	+	+	+	-	-	+	+
miR-381	-	-	+	-	+	-	-	-	+	-
miR-409	-	-	+	+	-	-	-	-	+	+
miR-410	+	-	+	+	+	-	+	-	+	+
miR-411	+	+	+	+	+	-	-	-	+	+
miR-421	+	-	+	-	+	-	-	-	+	-
miR-423	+	+	+	+	+	+	+	+	+	+
miR-425	+	+	+	+	+	+	+	+	+	+
miR-429	+	+	+	+	+	+	+	+	+	+
miR-431	-	-	-	-	-	-	-	-	+	-
miR-434	+	+	+	+	+	+	+	-	+	+
miR-449a	+	+	+	+	+	+	+	+	+	+
miR-450a	+	-	+	-	+	-	-	-	+	-
miR-450b	-	-	-	-	-	-	-	-	+	-
miR-451	+	-	+	-	+	-	-	-	-	-
miR-451a	-	-	-	-	-	-	-	-	+	+
miR-455	+	+	-	-	+	-	-	-	+	+
miR-463	-	-	-	-	-	-	+	+	+	+
miR-465a	-	-	+	-	+	-	+	-	+	-
miR-465b	-	-	-	-	-	-	+	-	+	-
miR-465c	-	-	+	-	+	-	+	-	+	-
miR-466a	-	-	-	-	-	-	+	-	+	-
miR-466b	-	-	-	-	+	-	-	-	+	-
miR-466c	-	-	-	-	+	-	-	-	+	-
miR-466e	-	-	-	-	-	-	+	-	+	-
miR-466i	-	-	-	-	-	-	-	-	+	+
miR-466p	-	-	-	-	+	-	-	-	+	-
miR-467a	+	-	+	-	+	-	+	-	+	-
miR-467b	-	-	-	-	-	-	+	-	+	-
miR-467c	-	-	-	-	+	-	+	-	+	-
miR-467d	+	+	-	-	+	-	+	+	+	+
miR-467e	-	-	-	-	+	-	+	-	+	-
miR-470	+	+	+	+	+	-	+	+	+	+
miR-471	-	-	-	-	-	-	+	+	+	+
miR-484	+	+	+	+	+	+	+	+	+	+
miR-486	+	+	+	+	+	-	+	-	-	-
miR-486a	-	-	-	-	-	-	-	-	+	-
miR-486b	-	-	-	-	-	-	-	-	+	-
miR-496a	-	-	-	-	-	-	-	-	+	+
miR-497a	-	-	-	-	-	-	-	-	+	-
miR-497	+	+	+	+	+	+	+	+	-	-
miR-499	-	-	-	-	-	-	+	+	-	-
miR-500	-	-	-	-	+	-	-	-	+	+
miR-501	+	+	+	+	+	+	+	+	+	+
miR-511	-	-	-	-	+	+	-	-	+	+
miR-532	+	+	+	+	+	+	+	+	+	+
miR-541	-	-	+	+	+	+	-	-	+	+
miR-542	-	-	-	-	-	-	-	-	+	+
miR-574	+	+	+	+	+	+	-	-	+	+
miR-582	+	+	-	-	+	-	-	-	+	+
miR-598	-	-	-	-	+	-	-	-	+	-
miR-615	-	-	-	-	+	-	-	-	+	+
miR-652	+	-	+	-	+	-	-	-	+	-
miR-664	-	-	-	-	-	-	-	-	+	+
miR-669a	+	-	-	-	+	-	+	-	+	-
miR-669b	-	-	-	-	+	-	-	-	-	-
miR-669c	+	-	-	-	+	-	+	-	+	-
miR-669d	-	-	-	-	+	-	+	-	-	-
miR-669f	-	-	-	-	+	-	-	-	-	-
miR-669l	-	-	-	-	+	-	-	-	-	-
miR-669o	-	-	-	-	+	-	+	-	+	-
miR-669p	+	-	-	-	+	-	+	-	+	-
miR-671	+	+	-	-	+	+	-	-	+	+
miR-672	+	+	+	+	+	+	+	+	+	+
miR-674	-	-	-	-	+	+	-	-	+	+
miR-676	+	+	+	+	+	+	+	+	+	+
miR-677	-	-	-	-	-	-	-	-	+	+
miR-690	-	-	-	-	-	-	-	-	+	+
miR-708	-	-	-	-	-	-	-	-	+	+
miR-709	-	-	-	-	-	-	-	-	+	+
miR-714	-	-	-	-	-	-	-	-	+	-
miR-720	+	-	-	-	+	-	+	-	-	-
miR-741	-	-	+	+	+	+	+	+	+	+
miR-743a	-	-	-	-	+	-	+	-	+	-
miR-743b	-	-	+	+	+	-	+	+	+	+
miR-744	+	+	+	+	+	+	+	+	+	+
miR-871	+	+	+	+	+	+	+	+	+	+
miR-872	+	+	+	+	+	+	+	+	+	+
miR-874	-	-	-	-	+	-	-	-	+	+
miR-878	-	-	-	-	+	-	+	+	+	+
miR-879	-	-	-	-	-	-	-	-	+	-
miR-880	-	-	-	-	-	-	+	+	+	+
miR-881	-	-	+	+	+	+	+	+	+	+
miR-883a	-	-	-	-	-	-	+	+	+	+
miR-883b	-	-	-	-	-	-	+	+	-	-
miR-1195	-	-	-	-	-	-	-	-	+	-
miR-1198	+	-	+	-	+	-	-	-	+	-
miR-1247	-	-	-	-	-	-	-	-	+	+
miR-1249	+	+	-	-	+	+	-	-	+	+
miR-1251	+	+	-	-	+	-	-	-	+	-
miR-1291	-	-	-	-	-	-	-	-	+	-
miR-1839	+	+	+	+	+	+	+	+	+	+
miR-1843	+	-	+	-	+	-	+	-	-	-
miR-1843a	-	-	-	-	-	-	-	-	+	+
miR-1843b	+	+	-	-	+	+	-	-	+	+
miR-1957a	-	-	-	-	-	-	-	-	+	-
miR-1981	-	-	-	-	-	-	-	-	+	+
miR-3068	-	-	-	-	+	+	-	-	+	+
miR-3096	+	-	+	-	+	-	-	-	-	-
miR-3096b	-	-	-	-	+	-	-	-	-	-
miR-3107	+	+	+	+	+	-	+	-	-	-
miR-3470a	-	-	-	-	-	-	-	-	+	-
miR-3470b	-	-	-	-	-	-	-	-	+	+
miR-3471	-	-	-	-	-	-	+	-	-	-
miR-3473a	-	-	-	-	-	-	-	-	+	-
miR-3473b	-	-	-	-	+	-	-	-	+	-
miR-3473e	-	-	-	-	-	-	-	-	+	+
miR-3473f	-	-	-	-	-	-	-	-	+	-
miR-3535	-	-	-	-	-	-	-	-	+	+
miR-5099	-	-	-	-	-	-	-	-	+	-
miR-5100	-	-	-	-	-	-	-	-	+	-
miR-5119	-	-	-	-	-	-	-	-	+	-
miR-5121	-	-	-	-	-	-	-	-	+	+
miR-5126	-	-	-	-	-	-	-	-	+	-
miR-5128	-	-	-	-	-	-	-	-	+	-
miR-5621	-	-	-	-	-	-	-	-	+	-
miR-6238	-	-	-	-	-	-	-	-	+	-
miR-6240	-	-	-	-	-	-	-	-	+	-
miR-6412	-	-	-	-	-	-	-	-	+	-
miR-6538	-	-	-	-	-	-	-	-	+	-
miR-6937	-	-	-	-	-	-	-	-	+	-
miR-7033	-	-	-	-	-	-	-	-	+	-
miR-7087	-	-	-	-	-	-	-	-	+	-

1. Nixon, B., Stanger, S. J., Mihalas, B. P., Reilly, J. N., Anderson, A. L., Tyagi, S., Holt, J. E. and McLaughlin, E. A. (2015). The microRNA signature of mouse spermatozoa is substantially modified during epididymal maturation. Biol Reprod 93, 91.
2. Sharma, U., Conine, C. C., Shea, J. M., Boskovic, A., Derr, A. G., Bing, X. Y., Belleannee, C., Kucukural, A., Serra, R. W., Sun, F. et al. (2016). Biogenesis and function of tRNA fragments during sperm maturation and fertilization in mammals. Science (New York, N.Y.) 351, 391-396.
3. Nixon, B., Stanger, S. J., Mihalas, B. P., Reilly, J. N., Anderson, A. L., Dun, M. D., Tyagi, S., Holt, J. E. and McLaughlin, E. A. (2015). Next Generation Sequencing Analysis Reveals Segmental Patterns of microRNA Expression in Mouse Epididymal Epithelial Cells. PLoS ONE 10, e0135605.

Table S5: Comparison of miRNAs identified in the epididymosomes of mice, humans and bulls

miRNA Family	Mature miRNA	Total Epididymosomes			Mouse		Bovine	
		Mouse	Bovine[1]	Human[2]	Caput	Cauda	Caput	Cauda
**miRNAs represented in mouse, bovine and human epididymosome datasets**								
let-7	*let-7a*	+	+	+	+	+	+	+
	let-7b	+	+	+	+	+	+	+
	let-7c	+	+	+	+	+	+	+
	let-7d	+	+	+	+	+	+	+
	let-7e	+	+	+	+	+	+	+
	let-7f	+	+	+	+	+	+	+
	let-7g	+	+	+	+	+	+	+
	let-7i	+	+	+	+	+	+	+
miR-10	*miR-10a*	+	+	+	+	+	+	+
	miR-10b	+	+	+	+	+	+	+
miR-15	*miR-15a*	+	+	+	+	+	+	-
	miR-15b	+	+	+	+	+	+	+
miR-16	*miR-16*	+	+	+	+	+	+	+
miR-17	*miR-17*	+	+	+	+	+	+	+
miR-18	*miR-18a*	+	+	+	+	+	+	+
miR-19	*miR-19b*	+	+	+	+	+	+	+
miR-20	*miR-20a*	+	+	+	+	+	+	+
miR-23	*miR-23a*	+	+	+	+	+	+	+
	miR-23b	+	+	+	+	+	+	+
miR-24	*miR-24*	+	+	+	+	+	+	+
miR-25	*miR-25*	+	+	+	+	+	+	+
miR-26	*miR-26a*	+	+	+	+	+	+	+
	miR-26b	+	+	+	+	+	+	-
miR-27	*miR-27b*	+	+	+	+	+	+	+
miR-29	*miR-29a*	+	+	+	+	+	+	+
	miR-29c	+	+	+	+	+	+	-
miR-30	*miR-30a*	+	+	+	+	+	+	+
	miR-30b	+	+	+	+	+	+	+
	miR-30c	+	+	+	+	+	+	+
	miR-30d	+	+	+	+	+	+	+
	miR-30e	+	+	+	+	+	+	+
miR-31	*miR-31*	+	+	+	+	+	+	+
miR-34	*miR-34a*	+	+	+	+	+	+	+
	miR-34c	+	+	+	+	+	+	+
miR-93	*miR-93*	+	+	+	+	+	+	+
miR-99	*miR-99a*	+	+	+	+	+	+	+
	miR-99b	+	+	+	+	+	+	+
miR-103	*miR-103*	+	+	+	+	+	+	+
miR-107	*miR-107*	+	+	+	+	+	+	+
miR-125	*miR-125b*	+	+	+	+	+	+	+
miR-132	*miR-132*	+	+	+	-	+	+	-
miR-140	*miR-140*	+	+	+	+	+	+	+
miR-148	*miR-148a*	+	+	+	+	+	+	+
miR-151	*miR-151*	+	+	+	+	+	+	+
miR-181	*miR-181a*	+	+	+	+	+	+	+
	miR-181b	+	+	+	+	+	+	+
miR-191	*miR-191*	+	+	+	+	+	+	+
miR-192	*miR-192*	+	+	+	+	+	+	+
miR-200	*miR-200a*	+	+	+	+	+	+	+
	miR-200b	+	+	+	+	+	+	+
	miR-200c	+	+	+	+	+	+	+
miR-205	*miR-205*	+	+	+	+	+	+	+
miR-210	*miR-210*	+	+	+	+	+	+	+
miR-221	*miR-221*	+	+	+	+	+	+	+
miR-222	*miR-222*	+	+	+	+	+	+	+
miR-331	*miR-331*	+	+	+	+	+	+	+
miR-342	*miR-342*	+	+	+	+	+	+	+
miR-345	*miR-345*	+	+	+	+	+	+	+
miR-361	*miR-361*	+	+	+	+	+	+	+
miR-423	*miR-423*	+	+	+	+	+	+	+
miR-425	*miR-425*	+	+	+	+	+	+	+
miR-455	*miR-455*	+	+	+	+	+	+	-
miR-532	*miR-532*	+	+	+	+	+	+	+
**miRNA Family**	**Mature miRNA**	**Total Epididymosomes**			**Mouse**		**Bovine**	
		**Mouse**	**Bovine[1]**	**Human[2]**	**Caput**	**Cauda**	**Caput**	**Cauda**
**miRNAs represented in mouse and human epididymosome datasets**								
miR-19	*miR-19a*	+	-	+	+	+	-	-
miR-22	*miR-22*	+	-	+	+	+	-	-
miR-27	*miR-27a*	+	-	+	+	+	-	-
miR-29	*miR-29b*	+	-	+	+	+	-	-
miR-92	*miR-92a*	+	-	+	+	+	-	-
	miR-92b	+	-	+	+	+	-	-
miR-98	*miR-98*	+	-	+	+	+	-	-
miR-100	*miR-100*	+	-	+	+	+	-	-
miR-106	*miR-106b*	+	-	+	+	+	-	-
miR-125	*miR-125a*	+	-	+	+	+	-	-
miR-130	*miR-130a*	+	-	+	+	+	-	-
	miR-130b	+	-	+	-	+	-	-
miR-134	*miR-134*	+	-	+	-	+	-	-
miR-135	*miR-135a*	+	-	+	+	+	-	-
miR-141	*miR-141*	+	-	+	+	+	-	-
miR-146	*miR-146a*	+	-	+	+	+	-	-
	miR-146b	+	-	+	+	+	-	-
miR-148	*miR-148b*	+	-	+	+	+	-	-
miR-149	*miR-149*	+	-	+	+	+	-	-
miR-150	*miR-150*	+	-	+	+	+	-	-
miR-152	*miR-152*	+	-	+	+	+	-	-
miR-155	*miR-155*	+	-	+	-	+	-	-
miR-182	*miR-182*	+	-	+	+	+	-	-
miR-183	*miR-183*	+	-	+	+	+	-	-
miR-185	*miR-185*	+	-	+	-	+	-	-
miR-187	*miR-187*	+	-	+	+	+	-	-
miR-193	*miR-193a*	+	-	+	-	+	-	-
	miR-193b	+	-	+	-	+	-	-
miR-194	*miR-194*	+	-	+	+	+	-	-
miR-196	*miR-196b*	+	-	+	-	+	-	-
miR-203	*miR-203*	+	-	+	+	+	-	-
miR-324	*miR-324*	+	-	+	+	+	-	-
miR-330	*miR-330*	+	-	+	+	+	-	-
miR-335	*miR-335*	+	-	+	+	+	-	-
miR-339	*miR-339*	+	-	+	+	+	-	-
miR-362	*miR-362*	+	-	+	+	+	-	-
miR-365	*miR-365*	+	-	+	+	+	-	-
miR-374	*miR-374b*	+	-	+	+	+	-	-
miR-375	*miR-375*	+	-	+	+	+	-	-
miR-378	*miR-378c*	+	-	+	+	+	-	-
miR-421	*miR-421*	+	-	+	+	+	-	-
miR-449	*miR-449a*	+	-	+	+	+	-	-
miR-500	*miR-500*	+	-	+	+	+	-	-
miR-501	*miR-501*	+	-	+	+	+	-	-
miR-574	*miR-574*	+	-	+	+	+	-	-
miR-652	*miR-652*	+	-	+	+	+	-	-
miR-664	*miR-664*	+	-	+	+	+	-	-
miR-671	*miR-671*	+	-	+	+	-	-	-
miR-744	*miR-744*	+	-	+	+	+	-	-
miR-874	*miR-874*	+	-	+	+	+	-	-
**miRNA Family**	**Mature miRNA**	**Total Epididymosomes**			**Mouse**		**Bovine**	
		**Mouse**	**Bovine[1]**	**Human[2]**	**Caput**	**Cauda**	**Caput**	**Cauda**
**miRNAs represented in mouse and bovine epididymosome datasets**								
miR-34	*miR-34b*	+	+	-	+	+	+	-
miR-128	*miR-128*	+	+	-	+	+	+	-
miR-138	*miR-138*	+	+	-	+	+	+	-
miR-199	*miR-199a*	+	+	-	+	+	+	-
miR-204	*miR-204*	+	+	-	+	+	+	+
miR-214	*miR-214*	+	+	-	+	+	+	-
miR-320	*miR-320*	+	+	-	+	+	+	+
**miRNA Family**	**Mature miRNA**	**Total Epididymosomes**			**Mouse**		**Bovine**	
		**Mouse**	**Bovine[1]**	**Human[2]**	**Caput**	**Cauda**	**Caput**	**Cauda**
**miRNAs represented in bovine and human epididymosome datasets**								
miR-21	*miR-21*	-	+	+	-	-	+	+
miR-20	*miR-20b*	-	+	+	-	-	+	-

Summary

Total number of epididymosome miRNAs identified in each species

Mouse	Bovine	Human
264	80	246

Total number of epididymosome miRNAs conserved between species

Human / Mouse	Bovine / Mouse	Human / bovine	Mouse / human / bovine
113	70	70	63

Comparison of % of epididymosome miRNAs identified in the present study that are also able to be detected among those previously reported in the bull and human

Percentage of bovine epididymosome miRNAs represented in mouse epididymosome dataset = 27%
Percentage of human epididymosome miRNAs represented in mouse epididymosome dataset = 43%
Percentage of bovine epididymosome miRNAs represented in human epididymosome dataset = 28%

Comparison of % of epididymosome miRNAs previously reported in the bull and human that were also identified in the present study

Percentage of bovine epididymosome miRNAs represented in mouse epididymosome dataset = 88%
Percentage of human epididymosome miRNAs represented in mouse epididymosome dataset = 46%
Percentage of human epididymosome miRNAs represented in bovine epididymosome dataset = 28%

miR-122	*miR-122*	-	+	+	-	-	-	+
miR-126	*miR-126*	-	+	+	-	-	+	+
miR-195	*miR-195*	-	+	+	-	-	+	+
miR-660	*miR-660*	-	+	+	-	-	+	+
miR-497	*miR-497*	-	+	+	-	-	+	-
**miRNA Family**	**Mature miRNA**	**Total Epididymosomes**			**Mouse**		**Bovine**	
		**Mouse**	**Bovine[1]**	**Human[2]**	**Caput**	**Cauda**	**Caput**	**Cauda**
**miRNAs represented only in mouse epididymosome datasets**								
let-7	*let-7j*	+	-	-	+	+	-	-
miR-1	*miR-1a*	+	-	-	-	+	-	-
miR-9	*miR-9*	+	-	-	+	+	-	-
miR-21	*miR-21a*	+	-	-	+	+	-	-
miR-28	*miR-28a*	+	-	-	+	+	-	-
miR-32	*miR-32*	+	-	-	+	+	-	-
miR-96	*miR-96*	+	-	-	+	+	-	-
miR-101	*miR-101a*	+	-	-	+	+	-	-
	miR-101b	+	-	-	+	+	-	-
	miR-101c	+	-	-	+	+	-	-
miR-126	*miR-126a*	+	-	-	+	+	-	-
miR-127	*miR-127*	+	-	-	+	+	-	-
miR-133	*miR-133a*	+	-	-	+	+	-	-
	miR-133b	+	-	-	-	+	-	-
miR-136	*miR-136*	+	-	-	+	+	-	-
miR-137	*miR-137*	+	-	-	-	+	-	-
miR-139	*miR-139*	+	-	-	-	+	-	-
miR-142	*miR-142a*	+	-	-	+	+	-	-
miR-143	*miR-143*	+	-	-	+	+	-	-
miR-144	*miR-144*	+	-	-	-	+	-	-
miR-145	*miR-145a*	+	-	-	+	+	-	-
	miR-145b	+	-	-	-	+	-	-
miR-181	*miR-181c*	+	-	-	+	+	-	-
	miR-181d	+	-	-	+	+	-	-
miR-184	*miR-184*	+	-	-	-	+	-	-
miR-186	*miR-186*	+	-	-	+	+	-	-
miR-190	*miR-190a*	+	-	-	+	+	-	-
miR-195	*miR-195a*	+	-	-	+	+	-	-
miR-196	*miR-196a*	+	-	-	-	+	-	-
miR-199	*miR-199b*	+	-	-	+	+	-	-
miR-208	*miR-208b*	+	-	-	-	+	-	-
miR-211	*miR-211*	+	-	-	+	-	-	-
miR-218	*miR-218*	+	-	-	+	+	-	-
miR-223	*miR-223*	+	-	-	+	+	-	-
miR-296	*miR-296*	+	-	-	+	-	-	-
miR-298	*miR-298*	+	-	-	+	-	-	-
miR-299	*miR-299a*	+	-	-	-	+	-	-
miR-300	*miR-300*	+	-	-	+	+	-	-
miR-301	*miR-301a*	+	-	-	+	+	-	-
miR-322	*miR-322*	+	-	-	+	+	-	-
miR-326	*miR-326*	+	-	-	-	+	-	-
miR-328	*miR-328*	+	-	-	+	+	-	-
miR-329	*miR-329*	+	-	-	-	+	-	-
miR-337	*miR-337*	+	-	-	-	+	-	-
miR-338	*miR-338*	+	-	-	+	+	-	-
miR-340	*miR-340*	+	-	-	+	+	-	-
miR-341	*miR-341*	+	-	-	+	+	-	-
miR-350	*miR-350*	+	-	-	-	+	-	-
miR-351	*miR-351*	+	-	-	+	+	-	-
miR-369	*miR-369*	+	-	-	-	+	-	-
miR-376	*miR-376b*	+	-	-	-	+	-	-
miR-378	*miR-378a*	+	-	-	+	+	-	-
	miR-378d	+	-	-	-	+	-	-
miR-379	*miR-379*	+	-	-	-	+	-	-
miR-381	*miR-381*	+	-	-	+	+	-	-
miR-409	*miR-409*	+	-	-	+	+	-	-
miR-410	*miR-410*	+	-	-	+	+	-	-
miR-411	*miR-411*	+	-	-	+	+	-	-
miR-429	*miR-429*	+	-	-	+	+	-	-
miR-431	*miR-431*	+	-	-	-	+	-	-
miR-434	*miR-434*	+	-	-	+	+	-	-
miR-450	*miR-450a*	+	-	-	+	+	-	-
	miR-450b	+	-	-	+	-	-	-
miR-451	*miR-451a*	+	-	-	+	+	-	-
miR-463	*miR-463*	+	-	-	-	+	-	-
miR-465	*miR-465a*	+	-	-	-	+	-	-
	miR-465b	+	-	-	-	+	-	-
	miR-465c	+	-	-	+	+	-	-
miR-466	*miR-466a*	+	-	-	+	-	-	-
	miR-466b	+	-	-	+	-	-	-
	miR-466c	+	-	-	+	-	-	-
	miR-466e	+	-	-	+	-	-	-
	miR-466i	+	-	-	-	+	-	-
	miR-466p	+	-	-	+	-	-	-
miR-467	*miR-467a*	+	-	-	+	+	-	-
	miR-467b	+	-	-	+	+	-	-
	miR-467c	+	-	-	+	-	-	-
	miR-467d	+	-	-	+	-	-	-
	miR-467e	+	-	-	+	-	-	-
miR-470	*miR-470*	+	-	-	+	+	-	-
miR-471	*miR-471*	+	-	-	-	+	-	-
miR-484	*miR-484*	+	-	-	+	+	-	-
miR-486	*miR-486a*	+	-	-	+	+	-	-
	miR-486b	+	-	-	+	+	-	-
miR-496	*miR-496a*	+	-	-	-	+	-	-
miR-497	*miR-497a*	+	-	-	+	+	-	-
miR-511	*miR-511*	+	-	-	-	+	-	-
miR-541	*miR-541*	+	-	-	+	+	-	-
miR-582	*miR-582*	+	-	-	+	+	-	-
miR-615	*miR-615*	+	-	-	+	+	-	-
miR-669	*miR-669a*	+	-	-	+	+	-	-
	miR-669c	+	-	-	+	-	-	-
	miR-669o	+	-	-	+	-	-	-
	miR-669p	+	-	-	+	+	-	-
miR-672	*miR-672*	+	-	-	+	+	-	-
miR-674	*miR-674*	+	-	-	+	+	-	-
miR-676	*miR-676*	+	-	-	+	+	-	-
miR-677	*miR-677*	+	-	-	+	+	-	-
miR-690	*miR-690*	+	-	-	+	+	-	-
miR-708	*miR-708*	+	-	-	-	+	-	-
miR-709	*miR-709*	+	-	-	-	+	-	-
miR-714	*miR-714*	+	-	-	+	-	-	-
miR-741	*miR-741*	+	-	-	+	+	-	-
miR-743	*miR-743a*	+	-	-	-	+	-	-
	miR-743b	+	-	-	+	+	-	-
miR-871	*miR-871*	+	-	-	+	+	-	-
miR-872	*miR-872*	+	-	-	+	+	-	-
miR-878	*miR-878*	+	-	-	-	+	-	-
miR-879	*miR-879*	+	-	-	+	-	-	-
miR-880	*miR-880*	+	-	-	-	+	-	-
miR-881	*miR-881*	+	-	-	+	+	-	-
miR-883	*miR-883a*	+	-	-	-	+	-	-
miR-1195	*miR-1195*	+	-	-	+	+	-	-
miR-1198	*miR-1198*	+	-	-	+	+	-	-
miR-1247	*miR-1247*	+	-	-	+	+	-	-
miR-1249	*miR-1249*	+	-	-	+	+	-	-
miR-1251	*miR-1251*	+	-	-	+	+	-	-
miR-1291	*miR-1291*	+	-	-	-	+	-	-
miR-1839	*miR-1839*	+	-	-	+	+	-	-
miR-1843	*miR-1843a*	+	-	-	+	+	-	-
	miR-1843b	+	-	-	+	+	-	-
miR-1957	*miR-1957a*	+	-	-	+	+	-	-
miR-1981	*miR-1981*	+	-	-	-	+	-	-
miR-3068	*miR-3068*	+	-	-	+	+	-	-
miR-3470	*miR-3470a*	+	-	-	+	+	-	-
	miR-3470b	+	-	-	+	+	-	-
miR-3473	*miR-3473a*	+	-	-	+	+	-	-

	miR-3473b	+	-	-	+	+	-	-
	miR-3473e	+	-	-	+	+	-	-
	miR-3473f	+	-	-	-	+	-	-
miR-3535	miR-3535	+	-	-	+	+	-	-
miR-5099	miR-5099	+	-	-	+	+	-	-
miR-5100	miR-5100	+	-	-	+	+	-	-
miR-5119	miR-5119	+	-	-	-	+	-	-
miR-5121	miR-5121	+	-	-	+	+	-	-
miR-5126	miR-5126	+	-	-	+	-	-	-
miR-5128	miR-5128	+	-	-	-	+	-	-
miR-6238	miR-6238	+	-	-	-	+	-	-
miR-6240	miR-6240	+	-	-	+	+	-	-
miR-6412	miR-6412	+	-	-	-	+	-	-
miR-6538	miR-6538	+	-	-	+	+	-	-
miR-6937	miR-6937	+	-	-	+	+	-	-
miR-7033	miR-7033	+	-	-	+	+	-	-
miR-7087	miR-7087	+	-	-	-	+	-	-
**miRNA Family**	**Mature miRNA**	**Total Epididymosomes**			**Mouse**		**Bovine**	
		**Mouse**	**Bovine[1]**	**Human[2]**	**Caput**	**Cauda**	**Caput**	**Cauda**
**miRNAs represented only in bovine epididymosome datasets**								
miR-92	miR-92	-	+	-	-	-	+	+
miR-145	miR-145	-	+	-	-	-	+	+
miR-106	miR-106	-	+	-	-	-	+	+
**miRNA Family**	**Mature miRNA**	**Total Epididymosomes**			**Mouse**		**Bovine**	
		**Mouse**	**Bovine[1]**	**Human[2]**	**Caput**	**Cauda**	**Caput**	**Cauda**
**miRNAs represented only in human epididymosome datasets**								
miR-18	miR-18b	-	-	+	-	-	-	-
miR-28	miR-28	-	-	+	-	-	-	-
miR-106	miR-106a	-	-	+	-	-	-	-
miR-197	miR-197	-	-	+	-	-	-	-
miR-198	miR-198	-	-	+	-	-	-	-
miR-202	miR-202	-	-	+	-	-	-	-
miR-297	miR-297	-	-	+	-	-	-	-
miR-320	miR-320a	-	-	+	-	-	-	-
	miR-320b	-	-	+	-	-	-	-
	miR-320c	-	-	+	-	-	-	-
	miR-320d	-	-	+	-	-	-	-
	miR-320e	-	-	+	-	-	-	-
miR-346	miR-346	-	-	+	-	-	-	-
miR-363	miR-363	-	-	+	-	-	-	-
miR-378	miR-378	-	-	+	-	-	-	-
miR-382	miR-382	-	-	+	-	-	-	-
miR-432	miR-432	-	-	+	-	-	-	-
miR-449	miR-449b	-	-	+	-	-	-	-
miR-483	miR-483	-	-	+	-	-	-	-
miR-487	miR-487b	-	-	+	-	-	-	-
miR-491	miR-491	-	-	+	-	-	-	-
miR-494	miR-494	-	-	+	-	-	-	-
miR-502	miR-502	-	-	+	-	-	-	-
miR-505	miR-505	-	-	+	-	-	-	-
miR-508	miR-508	-	-	+	-	-	-	-
miR-509	miR-509	-	-	+	-	-	-	-
miR-510	miR-510	-	-	+	-	-	-	-
miR-513	miR-513a	-	-	+	-	-	-	-
miR-514	miR-514b	-	-	+	-	-	-	-
miR-550	miR-550	-	-	+	-	-	-	-
miR-551	miR-551b	-	-	+	-	-	-	-
miR-572	miR-572	-	-	+	-	-	-	-
miR-575	miR-575	-	-	+	-	-	-	-
miR-595	miR-595	-	-	+	-	-	-	-
miR-602	miR-602	-	-	+	-	-	-	-
miR-625	miR-625	-	-	+	-	-	-	-
miR-628	miR-628	-	-	+	-	-	-	-
miR-629	miR-629	-	-	+	-	-	-	-
miR-638	miR-638	-	-	+	-	-	-	-
miR-639	miR-639	-	-	+	-	-	-	-
miR-658	miR-658	-	-	+	-	-	-	-
miR-659	miR-659	-	-	+	-	-	-	-
miR-663	miR-663	-	-	+	-	-	-	-
miR-665	miR-665	-	-	+	-	-	-	-
miR-675	miR-675	-	-	+	-	-	-	-
miR-720	miR-720	-	-	+	-	-	-	-
miR-762	miR-762	-	-	+	-	-	-	-
miR-769	miR-769	-	-	+	-	-	-	-
miR-877	miR-877	-	-	+	-	-	-	-
miR-885	miR-885	-	-	+	-	-	-	-
miR-886	miR-886	-	-	+	-	-	-	-
miR-888	miR-888	-	-	+	-	-	-	-
miR-890	miR-890	-	-	+	-	-	-	-
miR-891	miR-891a	-	-	+	-	-	-	-
miR-891	miR-891b	-	-	+	-	-	-	-
miR-892	miR-892a	-	-	+	-	-	-	-
	miR-892b	-	-	+	-	-	-	-
miR-933	miR-933	-	-	+	-	-	-	-
miR-939	miR-939	-	-	+	-	-	-	-
miR-940	miR-940	-	-	+	-	-	-	-
miR-1180	miR-1180	-	-	+	-	-	-	-
miR-1202	miR-1202	-	-	+	-	-	-	-
miR-1207	miR-1207	-	-	+	-	-	-	-
miR-1224	miR-1224	-	-	+	-	-	-	-
miR-1225	miR-1225	-	-	+	-	-	-	-
miR-1226	miR-1226	-	-	+	-	-	-	-
miR-1228	miR-1228	-	-	+	-	-	-	-
miR-1231	miR-1231	-	-	+	-	-	-	-
miR-1246	miR-1246	-	-	+	-	-	-	-
miR-1254	miR-1254	-	-	+	-	-	-	-
miR-1260	miR-1260	-	-	+	-	-	-	-
	miR-1260b	-	-	+	-	-	-	-
miR-1268	miR-1268	-	-	+	-	-	-	-
miR-1274	miR-1274b	-	-	+	-	-	-	-
miR-1275	miR-1275	-	-	+	-	-	-	-
miR-1280	miR-1280	-	-	+	-	-	-	-
miR-1281	miR-1281	-	-	+	-	-	-	-
miR-1292	miR-1292	-	-	+	-	-	-	-
miR-1299	miR-1299	-	-	+	-	-	-	-
miR-1301	miR-1301	-	-	+	-	-	-	-
miR-1307	miR-1307	-	-	+	-	-	-	-
miR-1308	miR-1308	-	-	+	-	-	-	-
miR-1469	miR-1469	-	-	+	-	-	-	-
miR-1825	miR-1825	-	-	+	-	-	-	-
miR-1826	miR-1826	-	-	+	-	-	-	-
miR-1908	miR-1908	-	-	+	-	-	-	-
miR-1909	miR-1909	-	-	+	-	-	-	-
miR-1910	miR-1910	-	-	+	-	-	-	-
miR-1914	miR-1914	-	-	+	-	-	-	-
miR-1915	miR-1915	-	-	+	-	-	-	-
miR-1975	miR-1975	-	-	+	-	-	-	-
miR-1979	miR-1979	-	-	+	-	-	-	-
miR-2110	miR-2110	-	-	+	-	-	-	-
miR-2277	miR-2277	-	-	+	-	-	-	-
miR-2861	miR-2861	-	-	+	-	-	-	-
miR-3065	miR-3065	-	-	+	-	-	-	-
miR-3124	miR-3124	-	-	+	-	-	-	-
miR-3128	miR-3128	-	-	+	-	-	-	-
miR-3131	miR-3131	-	-	+	-	-	-	-
miR-3141	miR-3141	-	-	+	-	-	-	-
miR-3147	miR-3147	-	-	+	-	-	-	-
miR-3148	miR-3148	-	-	+	-	-	-	-
miR-3149	miR-3149	-	-	+	-	-	-	-
miR-3162	miR-3162	-	-	+	-	-	-	-
miR-3175	miR-3175	-	-	+	-	-	-	-
miR-3178	miR-3178	-	-	+	-	-	-	-
miR-3180	miR-3180	-	-	+	-	-	-	-
miR-3185	miR-3185	-	-	+	-	-	-	-
miR-3187	miR-3187	-	-	+	-	-	-	-

miR-3188	*miR-3188*	-	-	+	-	-	-	-
miR-3191	*miR-3191*	-	-	+	-	-	-	-
miR-3194	*miR-3194*	-	-	+	-	-	-	-
miR-3195	*miR-3195*	-	-	+	-	-	-	-
miR-3196	*miR-3196*	-	-	+	-	-	-	-
miR-3197	*miR-3197*	-	-	+	-	-	-	-
miR-3201	*miR-3201*	-	-	+	-	-	-	-
miR-4253	*miR-4253*	-	-	+	-	-	-	-
miR-4259	*miR-4259*	-	-	+	-	-	-	-
miR-4270	*miR-4270*	-	-	+	-	-	-	-
miR-4271	*miR-4271*	-	-	+	-	-	-	-
miR-4281	*miR-4281*	-	-	+	-	-	-	-
miR-4286	*miR-4286*	-	-	+	-	-	-	-
miR-4298	*miR-4298*	-	-	+	-	-	-	-
miR-4299	*miR-4299*	-	-	+	-	-	-	-
miR-4306	*miR-4306*	-	-	+	-	-	-	-
miR-4322	*miR-4322*	-	-	+	-	-	-	-

1. Belleannee, C., Calvo, E., Caballero, J., and Sullivan, R. (2013) Epididymosomes convey different repertoires of microRNAs throughout the bovine epididymis. Biol Reprod 89, 30
1. Belleannee, C., Legare, C., Calvo, E., Thimon, V., and Sullivan, R. (2013) microRNA signature is altered in both human epididymis and seminal microvesicles following vasectomy. Hum Reprod 28, 1455-1467
